# Highly-sensitive label-free deep profiling of N-glycans released from biomedically-relevant samples

**DOI:** 10.1038/s41467-023-37365-4

**Published:** 2023-03-23

**Authors:** Anne-Lise Marie, Somak Ray, Alexander R. Ivanov

**Affiliations:** Barnett Institute of Chemical and Biological Analysis, Department of Chemistry and Chemical Biology, Northeastern University, 360 Huntington Ave., Boston, MA 02115 USA

**Keywords:** Mass spectrometry, Glycomics, Glycobiology

## Abstract

Alterations of protein glycosylation can serve as sensitive and specific disease biomarkers. Labeling procedures for improved separation and detectability of oligosaccharides have several drawbacks, including incomplete derivatization, side-products, noticeable desialylation/defucosylation, sample loss, and interference with downstream analyses. Here, we develop a label-free workflow based on high sensitivity capillary zone electrophoresis-mass spectrometry (CZE-MS) for profiling of native underivatized released N-glycans. Our workflow provides a >45-fold increase in signal intensity compared to the conventional CZE-MS approaches used for N-glycan analysis. Qualitative and quantitative N-glycan profiling of purified human serum IgG, bovine serum fetuin, bovine pancreas ribonuclease B, blood-derived extracellular vesicle isolates, and total plasma results in the detection of >250, >400, >150, >310, and >520 N-glycans, respectively, using injected amounts equivalent to <25 ng of model protein and nL-levels of plasma-derived samples. Compared to reported results for biological samples of similar amounts and complexity, the number of identified N-glycans is increased up to ~15-fold, enabling highly sensitive analysis of sample amounts as low as sub-0.2 nL of plasma volume equivalents. Furthermore, highly sialylated N-glycans are identified and structurally characterized, and untreated sialic acid-linkage isomers are resolved in a single CZE-MS analysis.

## Abstract (web summary)

Glycans can serve as disease biomarkers. Here, the authors use label-free capillary electrophoresis-mass spectrometry for N-glycan profiling of minute sample amounts, resolving and characterizing previously undetected highly sialylated glycans and linkage isomers in a single analysis.

## Introduction

Protein glycosylation plays a fundamental role in a wide variety of biological and physiological processes, including protein folding, cellular communication, and protein clearance^1,2^. Glycome alterations may be associated with various pathologies, including viral/bacterial infection, cardiovascular diseases, and cancer^3–5^. These findings inspired a significant interest in the discovery of novel diagnostic, prognostic, and treatment-monitoring disease biomarkers based on changes in glycosylation profiles in biofluids such as serum and plasma^6–8^. Recent studies have identified tumor-related N-glycan patterns in the blood of patients diagnosed with breast, prostate, ovarian, liver, or lung cancer^8,9^. Alterations of sialylation and fucosylation levels were reported in human pathologies and evaluated as potential disease biomarkers^10–12^. Liquid biopsies can target diverse classes of biomarkers and appear as an attractive alternative to biopsies of solid tissues. To date, a dozen of glycosylated proteins has been approved as disease biomarkers by the Food and Drug Administration and are currently used in clinical practice^13^. The use of released carbohydrates as serological biomarkers could result in the detection of cancers or other pathologies at earlier stages, compared to the currently used protein abundance-based biomarkers due to better sensitivity and specificity, and a few promising potential glycobiomarkers are currently investigated in clinical trials^13^.

To address numerous biological or clinical needs, advanced glycoprofiling techniques for the characterization of glycans in whole serum, plasma, or other blood-derived specimens (e.g., immunoglobulin G (IgG), fetuin, and extracellular vesicle (EV) isolates) must be developed. The high abundance of IgG in human serum (ca. 10-15 mg/mL)^14^ and its prominent role in the immune system make its glycome an ideal system to examine for changes in various pathological states^15–17^. Several studies reported altered IgG glycosylation patterns in patients with gastric^3^, ovarian^18,19^, and prostate^20^ cancers. Glycosylation profiles of IgG-based biotherapeutics are of high importance in biotechnology and biopharma research to ensure the potency, safety, and clinical efficacy of such biotherapeutic proteins, as well as their structural reproducibility and integrity in manufacturing and storage^21,22^. Fetuins are a group of glycoproteins belonging to the cystatin superfamily and are also secreted at high abundance into the bloodstream (ca. 0.2–0.8 mg/mL in human serum)^23^. Fetuins are multifunctional proteins that play important roles in diabetes, kidney, neurodegenerative, and oncology diseases^23,24^. Bovine serum fetuin (BSF), a highly sialylated glycoprotein, is widely used as a model protein for method development in glycoproteomics^25^. EVs are phospholipid bilayer membrane-enclosed submicron-size bodies secreted by various cells in biofluids^26,27^. EVs are highly heterogeneous and complex; they vary in size, composition (including, proteins, lipids, RNAs), and origin. Glycan profiling of EVs for the discovery of novel diagnostic disease biomarkers has gained increasing attention in both research and clinical contexts.

In glycomic research, N-glycans are traditionally analyzed by high-performance liquid chromatography (HPLC) using different modes, including reversed phase, hydrophilic interaction, and anion exchange. Most methods for the analysis of oligosaccharides from biological sources require a derivatization step to facilitate the separation of the glycans and improve their fluorometric, UV, or MS detection. A wide variety of derivatization strategies with a large number of labeling reagents were developed^28,29^. Released glycans are often derivatized with fluorophore compounds such as 8-aminopyrene-1,3,6-trisulfonic acid (APTS), 2-aminobenzamide (2-AB), or 2-aminobenzoic acid (2-AA)^30^. While these various labeling strategies are quite efficient in increasing the signal intensity levels of the detected glycans and may provide more accurate quantitation for specific glycan species, side reactions leading to the formation of multiple products may occur^31,32^, and some glycans may remain underivatized^33–35^. Moreover, reductive amination-based labeling strategies use acidic conditions, high temperatures, and/or high incubation times, and may significantly increase the risk of desialylation and defucosylation^36,37^. The additional steps associated with glycan derivatization can also eliminate common glycan modifications, e.g., O-acetylation of sialic acids^38,39^. Moreover, labeling procedures often require numerous cleaning steps in order to remove the excess of derivatizing reagent, which may lead to significant sample loss. Glycan labeling may also introduce a bias in the detection sensitivity of the glycans because of the purity of the labeling reagents^30^, the artifacts of derivatization (e.g., dehydration artifacts caused by a loss of water) generated during the labeling reaction^39^, and ionization suppression and mass spectrometry (MS) signal interference by the labeling reagent in MS-based glycan profiling techniques^40^. Therefore, the analysis of minimally altered during the sample preparation underivatized glycans may be preferred. MS-based analysis of non-labeled glycans is challenging, particularly in the commonly used positive ionization mode, because of the low hydrophobicity and low ionization efficiency of neutral and acidic native glycans. Recently, Szabo et al. reported an LC-MS-based method for the analysis of non-labeled glycans^39,41^. Glycan profiling of several model glycoproteins, including rhEPO and mAbs, was conducted and resulted in the assignment of ~40 N-glycans in each biological specimen.

With its high selectivity and resolving power, capillary electrophoresis (CE) is a powerful separation technique for glycan analysis. Recently, Taverna and co-workers developed an in-capillary APTS-labeling approach for fully automated mapping of N-glycans released from IgG and mAb^42^. In another recent work, CE was applied to the study of the deglycosylation kinetics of IgG^43^. The interfacing of CE with MS, using soft ionization techniques like electrospray ionization (ESI), enables unambiguous structural identification through both MS and tandem MS^44,45^. These last few years, novel glycomic strategies based on capillary zone electrophoresis (CZE) coupled to MS were developed^35,38,46–49^. Bunz et al. developed high-efficiency separation CZE-MS methods for the analysis of APTS-labeled and non-labeled glycans, which were applied to the profiling of biopharmaceuticals^46^. Chen and co-workers reported a CZE-MS method using a junction-at-the-tip interface for the simultaneous profiling of underivatized neutral and negatively charged sialylated glycans, which resulted in the detection of >70 N-glycans in rhEPO^38^.

In this study, we developed a high-sensitivity CZE-MS method for N-glycan profiling of proteins and proteomes derived from complex biological sources *without glycan derivatization*. For method development, high-purity model proteins isolated from human and bovine sera, and bovine pancreas were used as prototypes of biomedical samples. Then, the method was applied to glycan profiling of EVs and total plasma we isolated from human blood. CZE-MS analyses of native non-labeled N-glycans released with PNGase F were performed using a commercial sheathless CE-MS interface. A significant sensitivity improvement (on average, at least 45-fold increase in peak intensities) was achieved using dopant-enriched nitrogen (DEN)-gas combined with optimized ionization, desolvation, and CZE-MS conditions, as directly compared to the conventional mode of instrument operation^35^. Tandem MS in negative ESI mode provided highly informative MS^2^ spectra with extensive cross-ring fragmentation, which enabled unequivocal structural characterization, and differentiation of positional as well as linkage isomers. To benchmark, the reported numbers of N-glycans for high-purity model proteins and biomedically relevant specimens, a systematic comparison with the literature data was conducted. The method we developed resulted in up to 15-fold increased numbers of identified N-glycans, compared to the LC-MS and CE-MS methods reported for N-glycan profiling of scarce amounts of similar complexity blood and tissue isolates^16,38,50–54^, and allowed us to analyze and profile sample amounts equivalent to ∼100–200 pL of human plasma, which resulted in the detection of 60 to 210 N-glycans, among which not yet reported hexafucosylated and pentasialylated glycans were identified. The developed technique has the potential to provide important structural and quantitative information about glycome alterations caused by biological phenomena and pathologies using nL to pL-volume samples derived from physiological fluids and other biological systems.

## Results

### Development of a high sensitivity and high resolution CZE-MS method for label-free N-glycan profiling

#### Optimization of the detection sensitivity

**Fig. 1:**
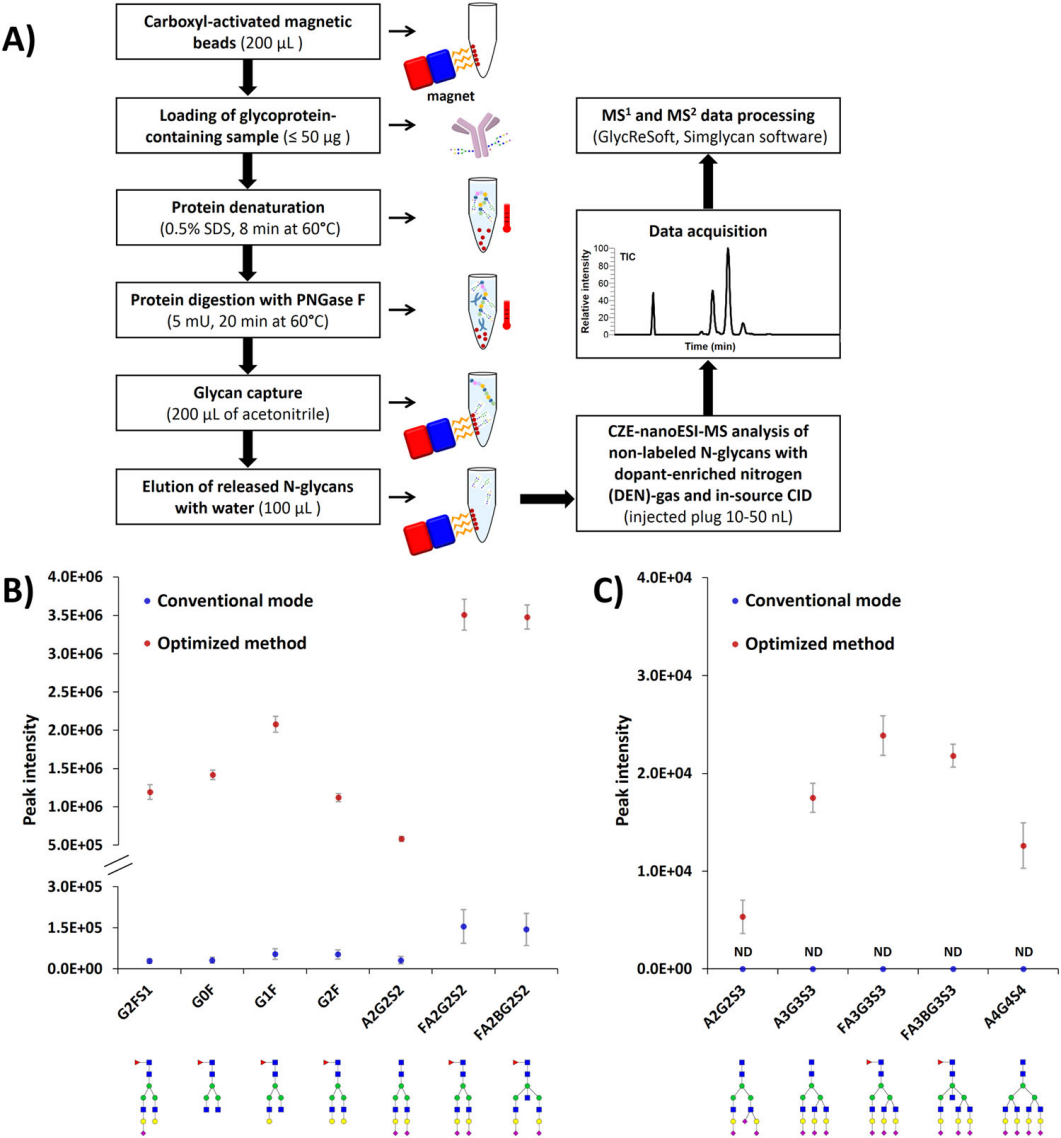
Analytical workflow and sensitivity improvement. **A** Analytical workflow for CZE-MS-based label-free glycan profiling of N-glycans released from biological sources (e.g., human or bovine sera). The total amount of N-glycans eluted in 100 µL of water correspond roughly to ≤1000 ng for human serum IgG. **B**, **C** The effect of experimental conditions on CZE-MS-based N-glycan qualitative and quantitative profiling. CZE-MS analyses (*n* = 3 technical replicates, data are presented as mean values ± the standard deviations (SD)) of N-glycans derived from human serum IgG using two different conditions: 1. conventional mode of instrument operation (ITT at 110 °C); and 2. nitrogen gas enriched with IPA (ITT at 150 °C) and ISCID at 70 eV (see Materials and Methods). **B** Selected highly abundant N-glycans. **C** Selected very low abundance N-glycans, which were not detected using the conventional mode of instrument operation. Glycan symbols: blue square, GlcNAc; red triangle, Fuc; green circle, Man; yellow circle, Gal; purple diamond, Neu5Ac. Source data are provided as a Source Data file.

In the present study, we optimized the CZE-MS method for profiling of N-glycans derived from highly complex blood and tissue isolates available in limited amounts. As in our previous work, carboxylic-coated magnetic beads were used for the enrichment of N-glycans released from glycoproteins by enzymatic digestion with PNGase F^35^. However, contrary to the previous work, no subsequent derivatization with APTS was performed, and consequently, no cleaning steps were required to remove the excess of the derivatizing reagent. Fig 1A depicts the sample preparation workflow used to isolate N-glycans at a high-ng level in their native non-labeled state. CZE-MS analyses of non-labeled N-glycans were performed in negative ESI mode. Under negative electrospray conditions, carbohydrates form [M-H]^-^ ions arising mainly from the deprotonation of sialic acid moieties (for acidic glycans) and hydroxyl groups (for neutral glycans)^55,56^. Similar to our recent study^35^, we used optimized CZE-MS conditions by combining a dopant-enriched nitrogen (DEN)-gas, supplied into the space where the nESI emitter and the inlet into the mass spectrometer are located, with optimized levels of ion transfer tube (ITT) temperature and in-source collision-induced dissociation (ISCID). We selected the same optimized MS parameters (ITT at 150 °C, and ISCID at 70 eV) in combination with DEN-gas with isopropanol (IPA) as a dopant and evaluated the gains in the sensitivity of CZE-MS for non-labeled N-glycans. While the exact physical mechanisms resulting in MS signal enhancement when applying a DEN-gas remain not fully characterized, it was suggested that the increase in ion intensities of the desolvated ions generated during the ESI process was most probably induced by the condensation of solvent molecules on the surface of the electrosprayed liquid droplets, and that the condensation enthalpy released by the bonding of the solvent molecules to the droplet surface was sufficient to effectively desorb and spray solute ions from the droplet surface^57^. As shown in Fig. 1B, using the optimized CZE-MS conditions, the peak heights of a set of representative non-labeled IgG N-glycans were increased up to 45-fold, as directly compared to the conventional mode of CZE-MS instrumentation typically used for N-glycan analysis (i.e., without DEN-gas and ISCID). Some of the shown N-glycans are typically of interest in characterization of biopharmaceutical mAbs. Similar results were observed for peak areas (Supplementary Fig. 1). The mean increase in the peak heights (and peak areas) was slightly lower (∼1.6-fold, i.e., 45-fold vs. 70-fold) under optimized conditions than that achieved when the same selected glycans were labeled with APTS^35^. The lower ionization efficiency of non-labeled glycans in negative ESI mode most probably explains this lower level of sensitivity improvement. Indeed, similar to the findings reported by other groups^46^, we observed that the ionization efficiency of non-labeled glycans was ~2.5-fold lower compared to their APTS-labeled analogs. Strikingly, this non-labeling strategy resulted in the detection of an increased number (~1.5-fold) of IgG N-glycans compared to the labeling approach, and 24 additional highly sialylated glycans were detected and quantified (see below). We attribute this difference to the above-discussed deficiencies of labeling approaches. Fig 1C displays a set of highly sialylated glycans that were detected only in the non-labeled IgG-derived samples. These non-labeled glycans were also not detected using the conventional mode of instrument operation, and therefore, in this case, the sensitivity improvement could be even more substantial than 45-fold.

#### Optimization of the separation performance

As we recently reported, to increase the resolution between glycan peaks, we found helpful to use an initial step (18 min) without applying supplemental pressure (SP) at the inlet of the bare-fused silica CE capillary, followed by the application of SP ranging from 2 to 5 psi^35^. The relatively low electroosmotic flow (EOF) mobility (2.02 × 10^−8^ m^2^/V/s) generated by the BGE consisting of 10 mM ammonium acetate pH 4.5 and 10% isopropanol, which is lower than the electrophoretic mobilities (µ_ep_) of the oligosaccharides (ranging from 2.7 ×10^−^^8^ to 3.8 ×10^−8^ m^2^/V/s), made the application of this step possible. A range of the SP from 2 to 5 psi was evaluated for the CZE-MS analysis of fetuin-derived glycans. The decrease of SP resulted in a significant extension of the separation window from 8 min (when SP of 5 psi was applied) to 20 min (with SP of 2 psi). Analyses could also be performed without SP during the entire time of the run and led to a dramatic extension of the separation window (>44 min) and the improved resolution in the separation of glycans. Thus, glycan species with the same degree of sialylation could be baseline separated. For example, the glycans A2G2S2 and A3G3S2 were separated with a poor resolution of 0.6 with SP of 5 psi, and baseline separated with resolution >2.7 when no SP was applied (Supplementary Fig. 2). Similar results were observed for the A2G2S3-A3G3S3 pair. The CZE-MS analyses performed without SP resulted in a 6-fold increase of the resolution between examined glycans and their fucosylated analogs (Supplementary Fig. 2) and, interestingly, enabled the separation of fucosylated isomers with either a core fucose or an outer arm fucose (see next section). In addition, α−2,6 and α−2,3 SiA linkage isomers could be efficiently separated for FA2G2S2 and A2BG2S2 (see next section). These positional and linkage isomers could not be separated with SP as low as 2 psi. The CZE-MS conditions also allowed us to detect and separate neutral N-glycans with subtle differences in their monosaccharide composition, as shown below. Based on our experimental results (CZE migration patterns), EOF measurements, and theoretical calculations (time required for neutral analytes to reach the inlet after turning on the voltage), we hypothesized that the *endogenous native* neutral glycans (i.e., glycans that do not possess a net charge in physiological fluids or tissues) detected and separated with our CZE-MS method got mobilized under the applied CZE conditions through ion-dipole intermolecular interactions with anions present in the BGE, presumably acetate anions^38^. Additional experiments performed by applying a continuous SP of 5 psi over the entire duration of the CZE-MS run resulted in the detection of neutral glycans that migrated before the neutral marker (acetaminophen) used to measure the EOF and supported the hypothesis that the detected neutral glycans acquired a negative charge during the CZE run (Supplementary Note 1).

The repeatability of the CZE-MS method was evaluated in quadruplicate experiments, where eight selected N-glycans with various degrees of sialylation and fucosylation were monitored. The intra-day repeatability of the method was appropriate for the CZE-based technique, where RSDs were less than 0.7% and 13% for migration times and peak areas, respectively (see Supplementary Note 2 and Supplementary Fig. 3).

### N-glycan profiling of total human serum IgG isolate

#### N-Glycan composition profiling

**Fig. 2:**
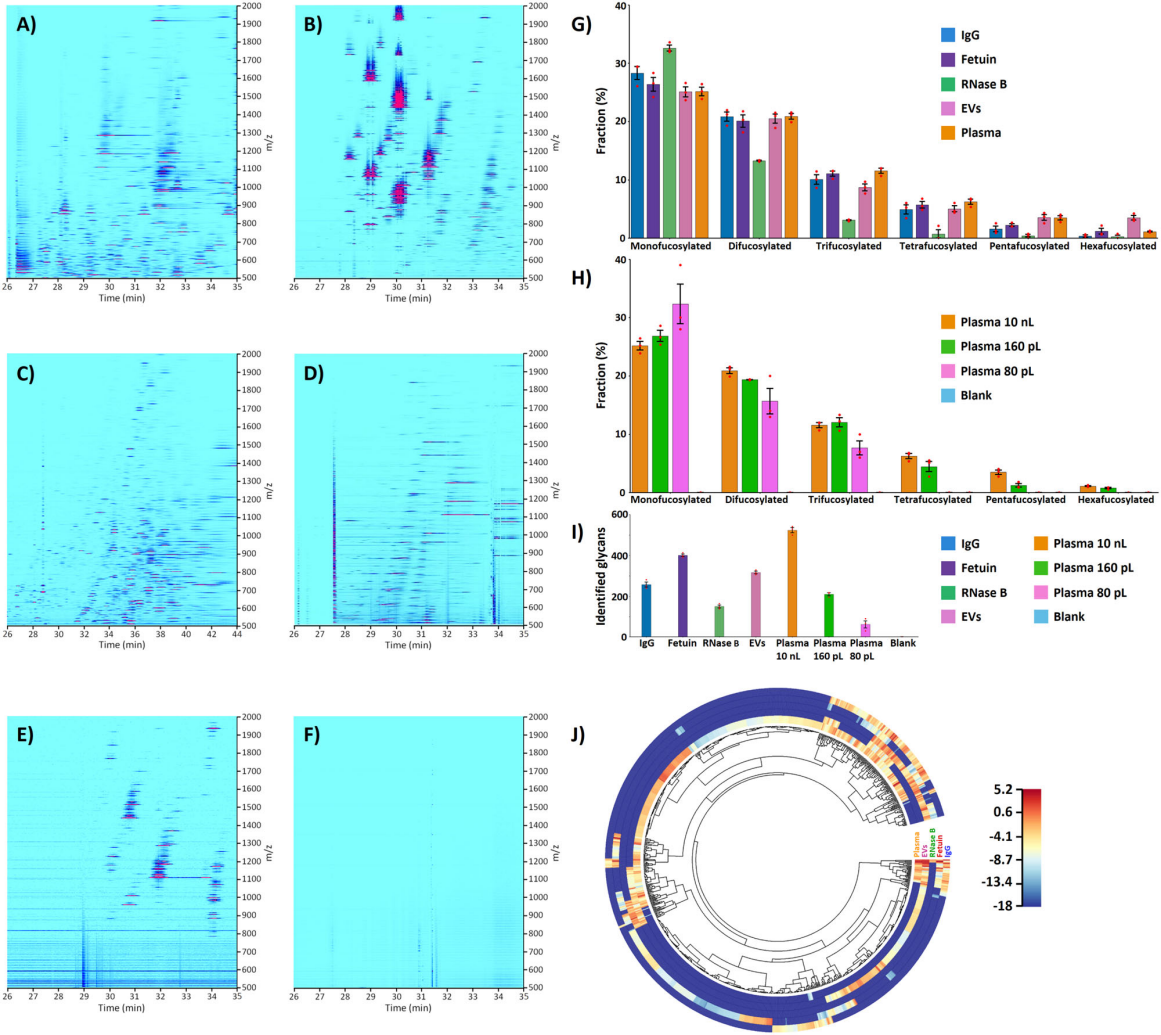
CZE-MS-based N-glycan profiling of biomedically relevant samples. **A**–**F** Ion density maps acquired from the label-free CZE-MS analyses of N-glycans released from (**A**) total human serum IgG isolate, (**B**) total BSF isolate, (**C**) bovine pancreas ribonuclease B isolate (injected sample amounts equivalent to 25, 5, and 15 ng of glycoproteins, respectively), (**D**) human plasma EVs, (**E**) total human plasma (injected sample amounts equivalent to ∼185 nL and ∼0.16 nL of plasma, respectively), and (**F**) blank sample of water. For IgG, BSF, EV and whole plasma isolates, the separation window was comprised between 26 and 35 min, whereas it was extended to 44 min for RNase B due to the detection of a high number of neutral N-glycans. **G** Fractional distributions of the fucosylated glycans detected in human serum IgG, BSF, bovine pancreas RNase B, human plasma EV, and total human plasma isolates. **H** Fractional distributions of the fucosylated glycans detected in sample amounts equivalent to ∼10 nL, ∼0.16 nL, and ∼0.08 nL of total human plasma. **I** Number of N-glycans identified in the five types of analyzed biological samples using the label-free CZE-MS method (*n* = 3 technical replicates, data are presented as mean values ± SD, red dots correspond to individual data points). **J** Differential qualitative and quantitative N-glycan profiling of the five biological samples (IgG, fetuin, RNase B, EV, and total plasma isolates). Circular heatmap showing Euclidean distance-based hierarchical clustering of all detected N-glycans in the five sample types. Red, yellow, and light blue colors correspond to high, medium, and low relative abundances based on the normalized N-glycan signal intensities. N-glycans that are not detected in the samples are highlighted in dark blue. The injected quantities of model proteins are as indicated above. For EV and total plasma isolates, the injected amounts were equivalent to ∼185 nL and ∼10 nL of human plasma, respectively. Source data are provided as a Source Data file.

CZE coupled with single-stage MS resulted in the identification of 258 ± 22 non-redundant N-glycan compositions (i.e., the compositional sum of monosaccharides) in human serum IgG isolate for injected amounts equivalent to ~25 ng of protein (i.e., ~3 nL of serum). Compared to reported studies of N-glycan profiling of serum IgG, the number of identified glycans was *increased ∼10-fold*^16,38,50,51^. The ion density map shown in Fig. 2A illustrates the characteristic CZE-MS migration patterns of non-labeled IgG glycan ions (governed by discrete changes in net charge and hydrodynamic volume) and the *m/z* shifts related to each monosaccharide mass increment. These CZE-MS migration patterns were used, amongst other criteria, in the validation of the glycan composition identification results to avoid misassignment of glycan species and to confirm negligible levels of in-source desialylation and defucosylation. Approximately 68% of the N-glycans detected in the IgG isolate were fucosylated, where ~29% were monofucosylated, ~21% difucosylated, ~10% trifucosylated, ~5% tetrafucosylated, ~2% pentafucosylated, and <1% hexafucosylated (Fig. 2G and Supplementary Fig. 4A) (due to their extremely low abundances, the MS/MS-based structural characterization of penta- and hexafucosylated N-glycans was not possible and their assignments were based on their migration time and single-stage MS). The detection of N-glycans composed of more than four fucose residues in human serum IgG isolates has not been reported yet by other groups. As observed in our recent work^35^, such highly fucosylated glycans could not be detected in the serum IgG isolate using the APTS-labeling strategy and an equivalent injected amount of N-glycans (~0.5 ng) per CZE-MS analysis (Supplementary Fig. 4A). We hypothesize that the numerous cleaning steps carried out to remove the excess of the derivatizing reagent induced a partial or complete loss of these extremely low abundance oligosaccharides. Also, fucose residues are chemically labile and may not withstand the acidic conditions of the derivatization process and/or cleaning steps^37^.

Approximately 60% of the detected IgG N-glycans were shown to be sialylated with 5-N-acetyl-neuraminic acid (Neu5Ac). ~32% of glycans were monosialylated, ~17% disialylated, ~6% trisialylated, ~3% tetrasialylated, and ~2% pentasialylated (Supplementary Fig. 4B). A lower level of sialylation (~33%) was observed in the IgG isolate using the labeling approach (Supplementary Fig. 4B), which indicates that glycan sialylation is better preserved when the glycans are not derivatized. Highly sialylated species (≥4 SiA residues) were detected at low abundance in the non-labeled IgG samples. These specific glycans were not detected in the IgG-derived N-glycan samples labeled with APTS using equivalent injected amounts of released N-glycans. In other types of human blood-derived isolates, we recently showed that highly sialylated glycans were problematic for APTS labeling (most probably due to electrostatic repulsion between APTS and the negatively charged glycan moieties during the labeling procedure), and such glycan species could only be detected in their non-labeled state in CZE-MS analysis^35^. Three hypotheses may explain why highly sialylated glycans (both labeled and unlabeled forms) were not detected in the IgG isolate using the APTS-labeling strategy: 1. the derivatization step induced total or partial desialylation of these specific glycans, 2. these glycans, present at very low abundances in the IgG isolate, may have been partially or completely lost during the cleaning steps performed to remove the excess of the labeling reagent, and 3. incomplete derivatization of these very low abundance glycans resulted in signal intensities for both labeled and unlabeled species below the LOD. As a result, the non-labeling approach led to the detection of 93 unique N-glycans, which were not detected using APTS-labeling (Supplementary Fig. 4C). Supplementary Fig. 4DE and Supplementary Data 1 show the results of the Euclidean distance-based hierarchical clustering of N-glycan quantitative profiles detected in human serum IgG with the APTS-labeling and label-free CZE-MS methods using injected amounts equivalent to ∼3 nL of serum. 82% of the glycans that were not detected with APTS labeling were sialylated glycans with up to 5 SiA residues. In contrast, 27% of the glycans that were not detected with the label-free approach were sialylated glycans with low levels of sialylation (mono- and disialylation). Similar trends were observed for the fucosylated glycans. 75% of the glycans uniquely detected with the label-free method were fucosylated glycans with up to 6 fucose residues, whereas 55% of the glycans uniquely detected with the APTS tag were low fucosylation level-glycans (1–3 fucose residues). Approximately half of the glycans commonly detected with both approaches were sialylated with 1 to 3 SiA residues. The set of sialylated glycans A2G2S2, FA2G2S2, and FA2BG2S2, detected at high abundance levels in the IgG isolate using the label-free approach (Fig. 1B), were also detected at high abundance levels using the APTS-labeling approach. The fucosylated glycans commonly detected with both approaches contained up to 4 fucose residues. The set of neutral glycans FA2G1, FA2G2, and FA3G1 were detected using both approaches but expectedly at relatively lower abundance levels using the label-free approach compared to the APTS-based strategy. Nevertheless, the abundance ratios between these three neutral fucosylated glycans were similar using both labeling and label-free approaches (e.g., the abundance level of FA2G1 was ∼3-times higher than that of FA3G1, as determined with both strategies). Finally, ∼50% of the total number of glycans commonly detected with both approaches exhibited insignificant differences in abundance levels between the APTS-glycans and their non-labeled counterparts (i.e., the abundance ratios of APTS glycan/non-labeled analog (or vice versa) did not exceed a factor of 2).

We demonstrated that higher levels of fucosylation and sialylation could be detected using the label-free approach due not only to a lesser extent of desialylation/defucosylation and sample loss during sample preparation but also because of lower levels of ESI- or in-source-induced decay.

We assessed the levels of in-source decay derived from in-source fragmentation of APTS-labeled and native non-labeled glycans selecting a set of sialylated glycans detected with both approaches at similar intensity levels. Significantly higher levels of in-source decay (>6%) were observed when the sialylated glycans were labeled with APTS than detected in their native non-labeled state. Using the label-free approach, the levels of in-source decay were either undetectable or negligible (<1%) (Supplementary Fig. 4F). Similar trends were observed for fucosylated glycans. The same drawbacks are expected to be manifested in any other glycan labeling approaches. Any labeling-based sample preparation procedure may lead to a well-known set of issues that include an artificial increase in the complexity of the sample due to incomplete labeling and aberrant over-labeling, formation of reaction byproducts, losses during sample labeling and cleanup steps, ion suppression due to the increased sample complexity and contamination with the labeling reagent(s). All these issues (1) are very challenging to tackle using limited sample amounts and (2) can lead to obscure identification and quantitation results. The described here approach is not affected by such limitations, which is evident from the presented depth of profiling.

#### CZE-MS^2^-based structural characterization

In this study, as anticipated, fragmentation of non-labeled glycans in negative ion mode, using higher energy collision dissociation (HCD), provided extensive and highly abundant cross-ring cleavages, enabling unambiguous characterization of structural features such as composition and branching of each antenna, location of fucose residues, and nature of SiA linkages, similarly to results reported by other groups^58,59^. Such level of in-depth structural characterization could not be obtained from tandem MS analysis of APTS-labeled glycans that generated MS^2^ spectra dominated by Y- and B-type glycosidic fragment ions, with low abundances of cross-ring cleavages^35^. High-quality MS^2^ spectra of native non-labeled glycans derived from total serum IgG isolate enabled reliable and accurate characterization of 156 N-glycan structures (i.e., exact monosaccharide arrangements and interconnecting glycosidic linkages) out of a total of 258 identified glycan compositions. Supplementary Data 2 presents an exhaustive list of the glycan structures identified in the human serum IgG isolate using the label-free glycan profiling approach. ~84% of the structures characterized by MS^2^ were complex-type glycans, ~7% high-mannose-type glycans, and ~9% hybrid-type glycans. Some polylactosamines could be detected as well (~5%).

In the field of biopharmaceutical and clinical research, glycan isomers must be differentiated since they may be of biological relevance. For instance, in breast cancer, isomers of both A2G2S2 and A3G3S3 N-glycans are detected at increased or decreased levels depending on the nature of the SiA linkage or the antenna branching^8^. The thorough structural characterization of representative IgG N-glycans is described in the following sections.

#### Disialylated N-glycans

The disialylated bi-antennary glycan **A2G2S2** (Mr_th_ 2222.78 Da) and its fucosylated and bisecting analogs were amongst the highest abundance N-glycans detected in the IgG isolate from total serum. The structurally informative MS^2^ spectra of these disialylated glycans illustrated the superior efficiency of HCD fragmentation in negative ion mode for non-labeled N-glycans compared to the MS fragmentation of APTS-labeled N-glycans. The MS^2^-based structural characterization of A2G2S2 was performed by selecting the [M-2H]^2−^ molecular ion at *m/z* 1110.38 as a precursor ion **(**Supplementary Fig. 5A**)**. The highest intensity fragment ion detected in the MS^2^ spectra of A2G2S2 was the singly charged B_1_ ion (*m/z* 290.09), corresponding to the loss of one SiA residue. The highly abundant doubly charged fragments B_6_ (*m/z* 999.84) and C_6_ (*m/z* 1008.84) indicated a glycosidic cleavage between the GlcNAc residues of the chitobiose core. The fragments Z_6_^1−^ (*m/z* 1912.65), Y_6_^1−^ (*m/z* 1930.66), and B_6_/Y_6_^1−^ (*m/z* 1709.58) enabled localization of the SiA binding sites at the termini of the antennae. B_3_^1−^ (*m/z* 655.22) and C_4_^1−^ (*m/z* 835.28) ions determined the composition of the antennae. The absence of a core fucose was documented by the characteristic mass difference of 60.02 Da between the cross-ring ions ^2,4^A_7_^2−^ (*m/z* 1029.85) and ^0,2^A_7_^2−^ (*m/z* 1059.86), and the cross-ring/glycosidic ions ^2,4^A_7_/Y_6_^1−^ (*m/z* 1769.61) and ^0,2^A_7_/Y_6_^1−^ (*m/z* 1829.63) ^60^. The detection of the singly charged diagnostic ion ^0,4^A_2_-CO_2_ at *m/z* 306.12 reflected the presence of at least one α−2,6-linked Neu5Ac^61^.

The MS^2^-based structural characterization of the fucosylated analog **FA2G2S2** (Mr_th_ 2368.84 Da), detected at higher abundance (∼7-fold) than A2G2S2, was performed by selecting the [M-2H]^2−^ molecular ion at *m/z* 1183.42 as a precursor ion (Supplementary Figs. 5B,6). The fragment ion B_1_^1−^ (*m/z* 290.09) was detected at high abundance. The presence of one additional fucosyl residue was supported by the shift of Z_6_^1−^ and Y_6_^1−^ fragment ions detected at *m/z* 2058.71 and 2076.72, respectively. The mass difference of 206.08 Da between the two pairs of ions ^2,4^A_7_^2−^ (*m/z* 1029.85) and ^0,2^A_7_^2−^ (*m/z* 1132.89), and ^2,4^A_7_/Y_6_^1−^ (*m/z* 1769.61) and ^0,2^A_7_/Y_6_^1−^ (*m/z* 1975.68) located the fucose on the chitobiose core^60^. Other fragments were largely similar to those obtained from the fragmentation of the non-fucosylated analog. The diagnostic ion ^0,4^A_2_-CO_2_^1−^ (*m/z* 306.12) was also observed in the mass spectra of the parent ions corresponding to the fucosylated glycan, indicating the presence of at least one α−2,6 SiA linkage, as in the previous example. To illustrate the significant differences between the fragmentation of non-labeled and APTS-labeled glycans in negative ion mode, Supplementary Fig. 7 shows examples of MS^2^ spectra of FA2G2S2 glycan without (panel A) or with (panel B) APTS-labeling. The fragmentation of FA2G2S2 in its non-labeled state generated *~10 times* as many structurally informative fragments as the fragmentation of the APTS-labeled counterpart (Supplementary Data 3). Cross-ring and cross-ring/glycosidic cleavage ions dominated the mass spectra of non-labeled FA2G2S2, accounting for ~75% of the total number of detected fragments for this glycan (Supplementary Data 3A). On the contrary, only 30% of the fragments detected in the mass spectra of APTS-labeled FA2G2S2 were derived from cross-ring and cross-ring/glycosidic cleavages (Supplementary Data 3B). It was reported that cross-ring cleavages required deprotonated hydroxyl groups in negative ion mode^55,62^. We hypothesize that the introduction of a negatively charged fluorescent tag at the reducing end of the glycans could induce the competition for deprotonation between the acidic groups of the tag and the hydroxyl groups of the glycans, making the deprotonation of the hydroxyl groups less favorable and thus decreasing the probability of cross-ring cleavages^59^. The fucosylated bisecting analog **FA2BG2S2** (Mr_th_ 2571.92 Da) was detected at a similar to FA2G2S2 abundance level (Fig. 1B). The MS^2^-based structural characterization of FA2BG2S2 is depicted in Supplementary Note 3 and Supplementary Fig. 8.

#### Tri- and tetrasialylated N-glycans

The reported here label-free glycan profiling approach allowed us to detect and structurally characterize highly sialylated glycans that we were not able to detect in the same samples using APTS labeling and similar sample amounts (equivalent to ~25 ng of total serum IgG)^35^. For example, tri- and tetrasialylated glycans, e.g., A3G3S3, FA3G3S3, A3G3S4, and A4G4S4, were detected at extremely low signal intensities in the total IgG isolate (see the fragmentation patterns of these glycans in next section). As shown in Fig. 1C, the signal intensities corresponding to these highly sialylated glycans were over ~300-fold lower compared to the signal intensities of the highly abundant disialylated FA2G2S2 and FA2BG2S2 glycan ions. Tri- and tetrasialylated glycans were reported as potential cancer biomarkers. For instance, increased levels of A4G4S4 were detected in the serum specimens of patients with breast, ovarian, and prostate cancer^8^. The described here results acquired using samples equivalent to only ∼3 nL of serum per analysis demonstrate the potential of the CZE-MS method to detect, identify, structurally characterize, and quantify extremely low abundance sialylated glycans that can potentially serve as biomarkers, using minimal amounts of liquid biopsies.

#### Neutral N-glycans

The developed technique provided the capability to successfully detect and characterize structural features of net-neutral N-glycans in the IgG isolate, e.g., FA2G1, FA2G2, FA3G1, and FA4F1BG1 (Supplementary Note 4 and Supplementary Fig. 9 for the structural characterization of these glycans). The MS detection of neutral glycans without derivatization is challenging because of the expectedly low ionization efficiency of these uncharged species in negative ESI mode as opposed to highly charged sialylated glycans. The SP applied during the CZE-MS analyses, the relatively low cathodic EOF, and possibly the acetate ion-glycan interaction, made the migration of these net-neutral glycans toward the anode possible. Neutral glycan moieties are expectedly important glycoprotein biomarkers. For example, unique neutral glycan structures, not detected in healthy control samples, were detected in serum samples of ovarian cancer patients^17^. Based on the demonstrated here results, our developed CZE-MS method is a promising approach to monitor the levels of neutral N-glycans in IgG isolates from human serum without glycan derivatization in minute amounts of samples at high sensitivity.

### N-glycan profiling of bovine serum fetuin isolate

#### N-Glycan composition profiling

**Fig. 3:**
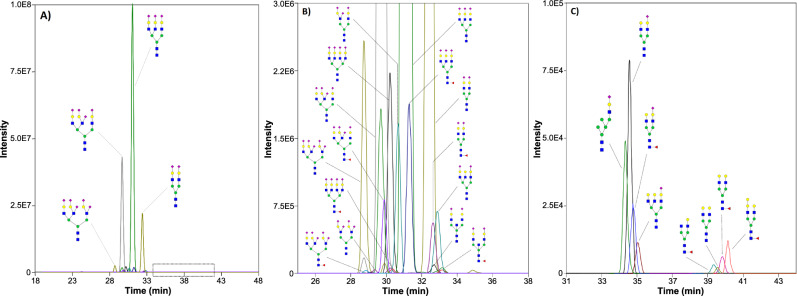
CZE-MS analysis of fetuin-derived glycans. Extracted ion electropherograms (EIEs) of 23 selected N-glycans detected in the BSF isolate. **A**, **B** EIEs of 15 highly abundant sialylated N-glycans (*panel B* is a zoomed region of *panel A* in the 25–38 min time range). Selected *m/z*: 1152.73; 1201.41; 933.98; 1055.69; 1104.38; 1177.40; 1226.09; 836.95; 958.66; 1007.35; 1110.38; 1183.41; 1211.92; 1284.95; and 1292.95; with a tolerance range of 20 ppm. **C** EIEs of 8 low abundance monosialylated and neutral N-glycans that migrate later than highly sialylated glycans (the dashed-line box in *panel A* delineates their zone of migration). Selected *m/z*: 944.32; 964.84; 1037.86; 1147.40; 811.29; 819.29; 892.32; and 973.34; with a tolerance range of 20 ppm.

The developed CZE-MS method resulted in the detection of 401 ± 11 non-redundant N-glycans in the BSF isolate for injected amounts equivalent to ∼5 ng of the protein (i.e., ~10 nL of serum), which represents *an ∼15-fold increased number* of N-glycans identified in bovine fetuin compared to other profiling studies of BSF isolates^52,54^. Fig 2B displays an example of an ion density map acquired in the CZE-MS analysis of fetuin N-glycans, which reflects the complexity of the fetuin glycome. As described above, the CZE-MS migration patterns of glycan ions and the *m/z* shifts were used in the glycan composition identification analysis. ~67% of the N-glycans detected in the fetuin isolate were shown to be fucosylated. ~26% of the glycans were monofucosylated, ~20% difucosylated, ~11% trifucosylated, ~6% tetrafucosylated, ~2% pentafucosylated, and ~1% hexafucosylated (Fig. 2G and Supplementary Fig. 10A). Approximately 72% of the detected fetuin N-glycans were sialylated with Neu5Ac and/or 5-N-glycolyl-neuraminic acid (Neu5Gc), another common mammalian sialic acid. In the total number of N-glycans containing Neu5Ac only, ~55% were monosialylated, ~27% disialylated, ~10% trisialylated, ~3% tetrasialylated, ~2% pentasialylated, <1% hexasialylated, and <2% heavily sialylated (≥7 SiA residues) (Supplementary Fig. 10C). The distribution of N-glycans containing Neu5Gc only was quite different with the following values: 51%, 34%, 13%, and 2% for mono-, di-, tri-, and tetrasialylated glycans, respectively (Supplementary Fig. 10E). It is noteworthy that the injection of 10 times lower sample amounts (equivalent to ∼0.5 ng of proteins, i.e., ∼1 nL of serum) resulted in the detection of 232 ± 15 N-glycans. Similar proportions of fucosylated species were measured compared to the above-described results, whereas slight differences were observed in the distribution of sialylated glycans. Nevertheless, due to their very low abundances in the fetuin isolate, hexafucosylated and highly sialylated glycans (≥5 SiA residues) could not be detected using these low-nL sample amounts (Supplementary Fig. 10BDF). Fig 3 shows the extracted ion electropherograms (EIEs) of 23 representative fetuin-derived N-glycans with various degrees of sialylation, fucosylation, and bisection. As can be observed, the migration times were governed by the number of sialic acids (the highly sialylated species migrating first, and the neutral glycans migrating last) and the hydrodynamic volume of the glycans.

#### CZE-MS^2^-based structural characterization

A large variety of sialylated N-glycans (from mono- to multisialylated), with a broad range of abundance levels, could be structurally characterized in the bovine fetuin isolate. The cross-ring-rich MS^2^ spectra acquired in the present study enabled in-depth structural characterization (the complexity of the spectra increasing with the size of the glycans and the degree of sialylation) and can serve as excellent reference spectra for the structural assignment of complex oligosaccharides (including previously uncharacterized ones) in heterogeneous and complex biological matrices. The following sections describe representative examples of fragmentation patterns of sialylated N-glycans released from bovine fetuin.

#### Trisialylated N-glycans

**Fig. 4:**
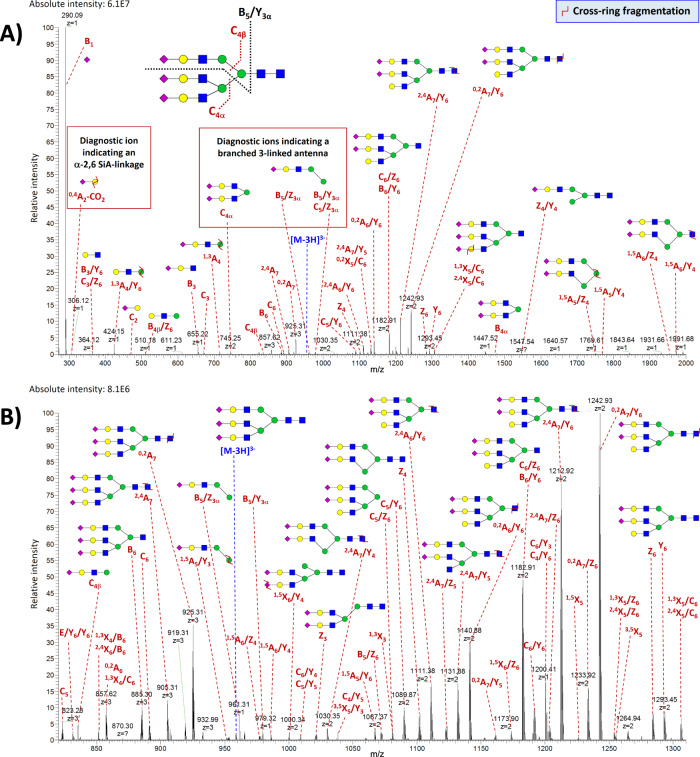
CZE-MS^2^-based structural characterization of trisialylated glycan. Example of the characteristic MS^2^ spectrum of A3G3S3 detected in the BSF isolate. The MS^2^-based structural characterization was performed by selecting the [M-3H]^3−^ molecular ion at *m/z* 958.66 as a precursor ion. Panels (**A**) and (**B**) show the same mass spectrum at different signal intensity and *m/z* ranges. Fragment ions are annotated based on the Domon and Costello nomenclature. Blue square, GlcNAc; red triangle, Fuc; green circle, Man; yellow circle, Gal; purple diamond, Neu5Ac. Symbol Ζ indicates cross-ring fragmentation. Only the most intense/relevant fragments are annotated in the shown spectrum.

The most abundant N-glycan detected in the BSF isolate was the tri-antennary trisialylated oligosaccharide **A3G3S3** (Mr_th_ 2879.01 Da). For the MS^2^-based structural characterization of this glycan, the triply charged ion at *m/z* 958.66 was selected as a precursor (Fig. 4). As shown in Supplementary Data 4, ~75% of the total number of detected fragments in the MS^2^ spectra of A3G3S3 were derived from cross-ring and cross-ring/glycosidic cleavages. The highest intensity fragment ion detected in these spectra was the fragment B_1_^1−^ (*m/z* 290.09), corresponding to the loss of one SiA residue. The singly charged B_5_/Z_3α_ (*m/z* 961.31) and B_5_/Y_3α_ (*m/z* 979.32) ions, formed by the loss of the 3-linked antenna (i.e., antenna attached to the carbon 3 of the core mannose, Supplementary Fig. 11) together with the N-glycan chitobiose core, enabled the assignment of a branched 3-linked antenna. These fragments were previously reported as diagnostic ions because they can serve to distinguish the 3- and 6-linked antennae of N-linked glycans due to a difference in the relative stability of substituents attached to each antenna^60^. On the contrary, B_5_/Y_3β_^1−^ ion (theoretical *m/z* 1635.56), which would represent the loss of the 6-antenna together with the chitobiose core, was not detected. C_4α_^2−^ (*m/z* 745.25) and C_4β_^1−^ (*m/z* 835.28) fragments were both detected in the mass spectra, but C_4β_ ion was much less abundant (∼4-fold) than C_4α_. Besides, the mass spectra exhibited a singly charged fragment ion at *m/z* 831, which could consist of two branches of the 3-antenna (having lost the SiA residues by internal fragmentation) and carbon atoms 1–4 from their attached mannose residue. This ion was also reported as a 3-branched antenna diagnostic ion^56^. A glycosidic cleavage between the GlcNAc residues of the chitobiose core was observed with the fragments B_6_^3−^ (*m/z* 884.96) and C_6_^3−^ (*m/z* 890.97) (Fig. 4). The fragments Z_6_^2−^ (*m/z* 1283.94), Y_6_^2−^ (*m/z* 1292.95), and Y_6_/Y_6_^1−^ (*m/z* 2295.81) made possible to localize the SiA binding sites at the termini of the antennae. The absence of a core fucose was confirmed by the typical mass difference of 60.02 Da between the pair of cross-ring ions ^2,4^A_7_^3−^ (*m/z* 904.97) and ^0,2^A_7_^3−^ (*m/z* 924.98), and the pair of cross-ring/glycosidic ions ^2,4^A_7_/Y_6_^2−^ (*m/z* 1212.41) and ^0,2^A_7_/Y_6_^2−^ (*m/z* 1242.42)^60^. Besides, the mass spectra exhibited the diagnostic ion ^0,4^A_2_-CO_2_^1−^ at *m/z* 306.12 that revealed the presence of α−2,6 Neu5Ac linkages^61^. Other fragments, including ^3,5^X_5_^2−^ (*m/z* 1255.93) and ^1,3^X_5_/C_6_^2−^ (*m/z* 1306.96) ions, confirmed the presence of α−2,6 SiA linkages in the trisialylated oligosaccharide.

The fucosylated form **FA3G3S3** (Mr_th_ 3025.07 Da) migrated shortly after the non-fucosylated analog, as expected. The analyses performed without applying SP over the whole duration of the CZE run made possible to separate (at resolution 0.6) this highly abundant fucosylated glycan from a positional isomer **A3F1G3S3** with an outer arm fucose (Supplementary Note 5 and Supplementary Figs. 12, 13 for the structural characterization of FA3G3S3 and A3F1G3S3). Most likely, the core fucosylated isomer possesses a larger hydrodynamic size, which may explain its lower µ_ep_ compared to the other isomer. The differentiation of fucose isomers (glycans with either a core fucosylation or an outer arm fucosylation) may be clinically and biologically relevant. For instance, increased levels of outer arm fucosylation were reported in the blood serum of ovarian cancer patients^18,19^. The results reported here demonstrate the capability of the CZE-MS method to separate and structurally elucidate two fucose isomers without using selective enzymatic digestions.

#### Tetrasialylated N-glycans

The second most abundant N-glycan detected in bovine fetuin was the tetrasialylated tri-antennary glycan **A3G3S4** (Mr_th_ 3170.11 Da). The structural characterization of this glycan was based on the triply charged precursor ion at *m/z* 1,055.69 (see Supplementary Note 6 and Supplementary Fig. 14AB). The fucosylated analog **FA3G3S4** was also detected in the fetuin isolate at low abundance levels (Supplementary Fig. 14C). Other tetrasialylated glycans were detected by MS^2^, e.g., the tetra-antennary oligosaccharide **A4G4S4** and its fucosylated analog **FA4G4S4** (Supplementary Note 6 and Supplementary Fig. 15).

#### Penta-, hexa- and heavily sialylated N-glycans

Besides the detection of the above-described N-glycans with a relatively low degree of sialylation, the developed highly sensitive CZE-MS method made possible to detect, separate, structurally characterize, and quantify highly sialylated glycans (up to 10 SiA residues), which could represent a new class of clinical biomarkers. Such glycans were rarely identified in previous glycomic studies and, consequently, they have not been evaluated as potential disease biomarkers yet.

**Fig. 5:**
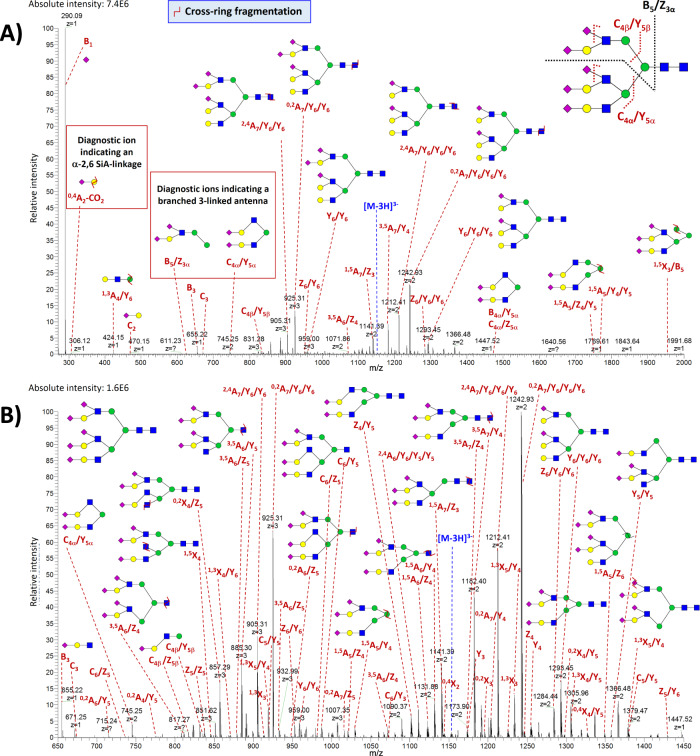
CZE-MS^2^-based structural characterization of pentasialylated glycan. Example of the characteristic MS^2^ spectrum of A3G3S5 detected in the BSF isolate. The MS^2^-based structural characterization was performed by selecting the [M-3H]^3−^ molecular ion at *m/z* 1152.73 as a precursor ion. Panels (**A**) and (**B**) show the same mass spectrum at different signal intensity and *m/z* ranges. See Fig. 4 for fragment ion annotations. Only the most intense/relevant fragments are annotated in the shown spectrum.

Pentasialylated glycans are complex and unusual structures that have been rarely reported in the literature^63–66^. Their detection and characterization with LC-MS methods remain difficult due to MS-incompatible high salt eluents typically used in anion exchange chromatography and the detection of low abundance peaks using hydrophilic interaction-based chromatography^66^. The CZE-MS method we developed allowed detailed structural characterization of pentasialylated glycans, based on high-quality and structurally informative MS^2^ spectra with extensive cross-ring fragmentation. For instance, the pentasialylated glycan **A3G3S5** (Mr_th_ 3461.20 Da), exhibiting a very high µ_ep_ (~3.56 × 10^−8^ m^2^/V/s), which is expected based on its five SiA residues, was detected. MS^2^ analysis of this highly sialylated glycan generated even more complex MS^2^ spectra (where ≥800 fragments were assigned per spectrum), which were dominated by ~72% of cross-ring and cross-ring/glycosidic fragment ions (Supplementary Data 5). The structural characterization of **A3G3S5** was based on the triply charged precursor ion at *m/z* 1152.73 (Fig. 5). The diagnostic ion B_5_/Z_3α_^2−^ ion at *m/z* 625.70 allowed the assignment of a branched 3-linked antenna. This ion also provided the composition of the 6-linked antenna and reflected the presence of two SiA residues on this antenna. The detection of the C_4α_/Y_5α_^2−^ fragment ion at *m/z* 745.25 in significantly higher abundance (∼7-fold) than the C_4β_/Y_5β_^1−^ ion at *m/z* 835.28 confirmed this assignment. The presence of α-2,6 SiA linkages was revealed by the detection of the diagnostic ion ^0,4^A_2_-CO_2_^1−^ at *m/z* 306.12. The fucosylated analog **FA3G3S5** was detected as well but in much lower abundance (∼70-fold) compared to the non-fucosylated analog. The mass difference of 206.08 Da between the two pairs of ions ^2,4^A_7_/Y_6_/Y_6_^3−^ (*m/z* 904.97) and ^0,2^A_7_/Y_6_/Y_6_^3−^ (*m/z* 973.66), and ^2,4^A_7_/Y_6_/Y_6_/Y_6_^2−^ (*m/z* 1212.41) and ^0,2^A_7_/Y_6_/Y_6_/Y_6_^2−^ (*m/z* 1315.45), derived from multiple internal fragmentations, confirmed the localization of the fucose on the chitobiose core (Supplementary Fig. 16A). The developed method also resulted in the MS^2^-based structural characterization of the hexasialylated glycan **A3G3S6** (Mr_th_ 3,752.30 Da) (Supplementary Fig. 16B), detected in the fetuin isolate at ∼1,000-fold lower abundance compared to A3G3S5 (Supplementary Fig. 17). As shown in Supplementary Fig. 17, the migration pattern of sialylated glycans was highly dependent on the level of sialylation, A3G3S6 migrating first and A3G3S1 migrating last. These separation results unequivocally demonstrate that the above-described detected sialylated glycans were not derived from ESI- or in-source-induced decay.

CZE-MS^2^ analysis of the fetuin isolate resulted in the detection and structural characterization of high molecular mass N-glycans with up to ten SiA residues. To date, only a few studies reported the detection of heavily sialylated N-glycans containing 7–11 SiA residues in biological specimens^67^. Supplementary Fig. 18A depicts the migration pattern of a set of heavily sialylated glycans detected in the CZE-MS analysis of fetuin isolate, which contain seven to ten Neu5Ac residues, and differ from each other by one Neu5Ac monosaccharide. Putative structures were proposed based on the fragmentation patterns of these large size glycans (Mr_th_ ranging from 5739.01 to 6612.30 Da), which were not reported yet in the literature. As no characteristic and intense fragment ion corresponding to a lactone form (*m/z* 272.09), indicating the presence of polysialic acids, was detected in the MS/MS spectra of the heavily sialylated glycans, we assumed that the sialic acids were located at the termini of the antennae. In addition, based on the detection of a set of fragment ions that were also detected in the MS/MS spectra of A3G3S4, A3G3S5, and A3G3S6 (e.g., at *m/z* 904.97, 924.98, 1182.40, 1212.42, 1242.42, and 1379.48), we assumed that these heavily sialylated N-glycans possessed a similar structure with six antennae and no core fucosylation (Supplementary Note 7 and Supplementary Fig. 19).

#### Neu5Gc-containing N-glycans

N-glycans from bovine fetuin contain two types of sialic acids, Neu5Ac and Neu5Gc. There is a critical need for the development of highly sensitive analytical methods to detect and quantify glycans containing the immunogenic carbohydrate Neu5Gc, which is detected at high levels in oncological and inflammatory diseases. The label-free CZE-MS method we developed enabled the detection and structural characterization of a large panel of Neu5Gc-containing glycans (∼10% of the total characterized structures) in the BSF isolate, using sample amounts equivalent to ∼10 nL of serum. For instance, the disialylated glycan **A2G2S2** was detected as a set of three different glycans terminated with either two Neu5Ac, or two Neu5Gc, or both Neu5Ac and Neu5Gc (Supplementary Note 8 and Supplementary Fig. 20 for the structural characterization of these glycans). Heavily sialylated N-glycans with 7–9 total SiA residues containing one Neu5Gc monosaccharide per glycan (Mr_th_ ranging from 5755.01 to 6337.20 Da) were also detected and characterized in the bovine fetuin isolate (Supplementary Fig. 21**)**. The MS^2^ spectra of these glycans exhibited the B_1_^1−^ fragment ion at *m/z* 306.08, corresponding to the loss of the Neu5Gc residue. The MS^2^ analysis of these Neu5Gc-containing N-glycans showed that their structures were similar to those above-described for heavily sialylated N-glycans carrying 7–10 Neu5Ac residues. As shown in Supplementary Fig. 18B, the migration times of these Neu5Gc-containing glycans were in good agreement with the level of sialylation as well as the migration times of their glycan counterparts composed solely of Neu5Ac residues. As the mass difference between one Neu5Ac and one Neu5Gc residues is only 15.99 Da (i.e., one oxygen atom) (Supplementary Fig. 21C), our developed CZE-MS method did not allow us to achieve a significant migration time shift between Neu5Gc-containing glycans and their respective counterparts carrying only Neu5Ac residues.

#### Separation of near-isobaric N-glycans

The monosialylated glycan **A2G2S1** (Mr_th_ 1931.69 Da) was detected in the fetuin isolate and structurally characterized by MS^2^ (Supplementary Fig. 22A**)**. The predominant fragment ions detected in the MS^2^ spectra of A2G2S1 were B_1_^1−^ (*m/z* 290.09), B_3_^1−^ (*m/z* 655.22), and C_4_^1−^ (*m/z* 835.28) ions, in good agreement with the presence of one Neu5Ac residue at the terminus of an antenna. The ^0,4^A_2_-CO_2_^1−^ ion at *m/z* 306.12, which is diagnostic for the α-2,6 linkage, was missing despite the high intensity of the B_1_ ion, which supported the assignment of the α-2,3 Neu5Ac linkage. Interestingly, a glycan with a molecular mass shift of one Da (Mr_th_ 1932.71 Da) compared to A2G2S1 migrated 1 min later. Both glycans were effectively separated at resolution >1.5 (Supplementary Fig. 23). MS^2^ data confirmed that the later migrating species was the difucosylated non-sialylated glycan **FA2F1G2**. For this glycan, the fragment ions ^0,2^A_6_/Z_1_^1−^ (*m/z* 247.08) and ^1,3^X_4_/B_3_^1−^ (*m/z* 425.13) helped identify one core fucose and one outer arm fucose, respectively (Supplementary Fig. 22B).

#### Separation of SiA linkage isomers

**Fig. 6:**
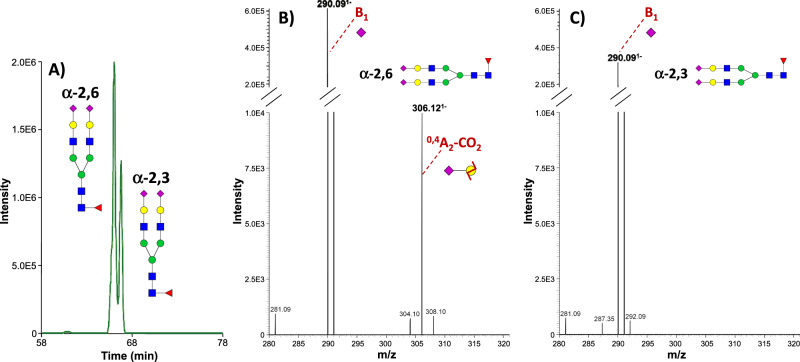
Differentiation of linkage isomers. CZE-MS-based separation of FA2G2S2 sialic acid-linkage isomers released from the BSF isolate. **A** EIE of FA2G2S2 glycan (at *m/z* 1183.42 ± 0.02 (i.e., ± 20 ppm)). **B**, **C** MS^2^ spectra of α−2-6- and α-2-3-linked sialic acid isomers showing the presence (**B**) or absence (**C**) of the α-2-6 linkage diagnostic ion ^0,4^A_2_-CO_2_^1−^ at *m/z* 306.12.

SiA linkage determines specific biological activities and altered levels of α-2,6- or α-2,3-SiA linkages are associated with oncological diseases^10,11,68^. We evaluated the capability of the developed CZE-MS method to separate glycan isomers that differ by the nature of the SiA linkages. The fucosylated glycan **FA2G2S2**, with two Neu5Ac residues, was detected in the fetuin isolate (see the discussion and Supplementary Fig. 5B for the fragmentation pattern of this glycan in the previous section). Interestingly, the analyses performed without applying the SP over the whole duration of the run resulted in the separation of α-2,6 and α-2,3 SiA linkage isomers at resolution >1.2, which would be a challenge for other separation approaches. As shown in Fig. 6, only the MS^2^ spectrum of the earlier migrating species exhibited the α-2,6 diagnostic ion at *m/z* 306.12 despite similar intensity levels of the B_1_ ions detected in the MS^2^ spectra of both species. This finding indicates that the separated glycans differ by their SiA linkages and that the later migrating species carries at least one α-2,3-linked SiA. α-2,6 and α-2,3 SiA isomers were also resolved for the bisecting form **A2BG2S2** when no pressure was applied (Supplementary Fig. 24). For the latter glycan, α-2,3 isomer was ∼1.5-fold more abundant than α-2.6 isomer.

#### Neutral N-glycans

The optimized CZE-MS^2^ method allowed us to detect, separate, quantitate, and structurally characterize unaltered *endogenous* neutral N-glycans in the fetuin isolate (see the above discussion regarding the separation mechanism of neutral glycans). As shown in Fig. 3 and Supplementary Fig. 3, the neutral N-glycans migrated later than the sialylated glycans, and a strong correlation was observed between the migration patterns of the detected neutral glycans and their respective monosaccharide composition. The developed CZE-MS^2^ technique made possible the structural characterization of neutral N-glycans like **FA2G2** and **FA2G3** (Supplementary Note 9 and Supplementary Figs. 9B and 25).

The conducted CZE-MS^2^ experiments expectedly did not result in the structural characterization of all N-glycans detected and identified using single-stage MS in the total fetuin isolate because of the stochastic mode of MS^2^ data-dependent acquisition, and because MS^2^ spectra acquired for low abundance precursors were insufficiently informative for reliable and accurate structural characterization. Nevertheless, the MS^2^ spectra that did not lead to reliable characterization of glycan structural features were examined manually to verify if their MS^2^ spectral patterns were characteristic of glycan fragmentation. This manual verification, together with the assessment of the migration patterns, helped confirm the MS^1^-based glycan composition identification results (see Supplementary Methods). Finally, the MS^2^ spectra resulted in the characterization of 123 N-glycan structures in the BSF isolate out of a total of 401 identified glycan compositions (Supplementary Data 6) and enabled confirmation of the glycan composition of some highly sialylated glycans (containing up to ten SiA residues), which have not yet been reported in the literature. Approximately 85% of the identified sialylated N-glycans contained Neu5Ac only (1 to 10 residues), ~6% Neu5Gc only (1 to 3 residues), and ~9% both SiA residues (Supplementary Data 6). Approximately 88% of the characterized structures were complex-type glycans, ~5% high-mannose-type glycans, and ~7% hybrid-type glycans. Some polylactosamines were detected as well (~9%).

### N-glycan profiling of bovine pancreas ribonuclease B

Ribonuclease B (RNase B) is a ∼17 kDa glycoprotein with a single N-glycosylation site at Asn34. It is frequently used as a model glycoprotein in the glycomics field for method development and performance assessment of analytical techniques for characterization of neutral glycans in biomedical samples. In recent studies, glycan profiling of serum RNase isolates was evaluated for the diagnosis of pancreatic cancer^69,70^. To evaluate the potential of the developed CZE-MS method for N-glycan profiling of glycoproteins with a lower level of sialylation, N-glycans released from bovine pancreas RNase B were analyzed (Fig. 2C). The glycome of RNase B is highly heterogeneous in high-mannose structures, as reported before^71–73^. Single-stage MS resulted in the identification of 150 ± 13 non-redundant N-glycans in RNase B isolate for injected amounts equivalent to ∼15 ng of total protein, which represents *a ∼10-fold increased number* of identified N-glycans in RNase B compared to literature data^53^. Approximately half of them were found to be fucosylated: ~33% were monofucosylated, ~13% difucosylated, ~3% trifucosylated, ~1% tetrafucosylated, <1% pentafucosylated, and <1% hexafucosylated (Fig. 2G). 48% of the detected RNase B glycans were sialylated with Neu5Ac and/or Neu5Gc residues. As described above, much higher levels of sialylation were measured in IgG and fetuin isolates (60% and 72%, respectively). Moreover, tetra- and pentasialylated glycans were not detected in the RNase B isolate. In the total number of N-glycans containing Neu5Ac only, ~76% were monosialylated, ~17% disialylated, and ~7% trisialylated.

CZE-MS^2^ experiments enabled the reliable characterization of 36 glycan structures in the RNase B isolate (Supplementary Data 7), among which 12 oligomannose-type glycans from Man3 to Man13 (glycan compositions corresponding to Man14 and Man15 were also confidently detected, but the structures could not be confirmed by MS^2^). The fragmentation pattern of Man13 is described in Supplementary Note 10 and Supplementary Fig. 26 as a representative example. The reported here results demonstrate the applicability of the label-free CZE-MS method to the profiling of biomedical samples with a low degree of sialylation.

#### N-glycan profiling of blood-derived extracellular vesicles

The label-free CZE-MS method we developed and optimized was applied to the analysis of N-glycans released from EVs isolated from human blood plasma (see Supplementary Methods for sample preparation). Single-stage MS analysis resulted in the identification of 317 ± 11 non-redundant N-glycans in the purified total EV isolate for injected amounts equivalent to ∼185 nL of plasma. The fractional distributions of fucosylated N-glycans (67% in total) detected in the total EV isolate accounted for ~25%, ~20%, ~9%, ~5%, ~4%, and ~4%, for mono-, di-, tri-, tetra-, penta-, and hexafucosylated N-glycans, respectively (Fig. 2G). Approximately 73% of the N-glycans detected in the EV isolate were sialylated with Neu5Ac and/or Neu5Gc. The fractional distributions of N-glycans containing only Neu5Ac accounted for ~47%, ~24%, ~15%, ~8%, ~4%, <1%, and ~2% for mono-, di-, tri-, tetra-, penta-, hexa-, and heptasialylated N-glycans, respectively. These numbers were respectively ~49%, ~31%, ~14%, ~5%, and ~1%, for mono-, di-, tri-, tetra-, and pentasialylated N-glycans containing only Neu5Gc. Such levels of sialylation with up to 7 SiA residues per glycan were not reported before in blood-derived EV isolates. Overall, the developed CZE-MS method resulted in an unsurpassed number of N-glycans detected in EVs isolated from biofluids using injected amounts as low as ∼185 nL of plasma (i.e., ∼500 nL of blood)^35,74^.

CZE-MS^2^ analysis of the purified EV isolate resulted in the structural characterization of 50 N-glycans (Supplementary Data 8). The most abundant N-glycans detected and fully structurally characterized by MS^2^ in the EV isolate were A2G2S2 and its fucosylated FA2G2S2 and bisecting FA2BG2S2 analogs, and A3G3S3 and its fucosylated analog FA3G3S3. Some tetrasialylated glycans, e.g., A3G3S4 and A4G4S4, were also detected at relatively high abundance. The molecular masses and fragmentation patterns of these glycans are discussed in the above sections.

Finally, we conducted a direct and thorough qualitative and quantitative comparison of the N-glycans detected and identified in human plasma EV isolates with the APTS-labeling and label-free CZE-MS methods using similar amounts of human plasma (Supplementary Fig. 27 and Supplementary Data 9). As shown in Supplementary Fig. 27, 173 N-glycans were uniquely detected using APTS-labeling. 33% of these unique glycans were non-sialylated glycans and 52% were mono- or disialylated glycans. The label-free method resulted in the detection of 208 unique N-glycans, including hexa- and heptasialylated glycans that were not detected with the APTS tag (non-sialylated glycans accounted for 11% in this set of unique glycans). 112 N-glycans were commonly detected with both strategies, and ∼60% of these common glycans (including A2G2S2, FA2G2S2, A3G3S3, FA3G3S3, and A3G3S4 above-discussed) exhibited not significantly different abundance levels between the APTS-glycans and their non-labeled counterparts.

#### N-glycan profiling of blood-derived total plasma

The developed label-free CZE-MS method was applied to the analysis of N-glycans released from total human plasma, another highly complex blood-derived isolate. The N-glycome analysis of human plasma represents another minimally invasive approach in the identification and monitoring of disease biomarkers. 523 ± 21 non-redundant N-glycans were identified in the total plasma isolate using injected amounts equivalent to *∼10 nL of plasma*. The fractional distributions of fucosylated N-glycans (69% in total) detected in whole plasma accounted for ~25%, ~21%, ~12%, ~6%, ~4%, and ~1%, for mono-, di-, tri-, tetra-, penta-, and hexafucosylated N-glycans, respectively (Fig 2G, H). The fractional distributions of Neu5Ac-containing glycans were as follows: ~31%, ~24%, ~16%, ~13%, ~9%, ~2%, and ~5%, for mono-, di-, tri-, tetra-, penta-, hexa-, and heavily (≥7 SiA residues) sialylated glycans, respectively. To evaluate the sensitivity level of the label-free CZE-MS method in the N-glycan profiling of limited biological and clinical specimens, sample amounts equivalent to sub-nL volume of total human plasma were analyzed (Fig. 2E). The analysis of ~0.16 nL of plasma-equivalent volume resulted in the detection of 210 ± 12 N-glycans, among which highly fucosylated (up to 6 fucose residues, Fig. 2H) and highly sialylated (up to 5 SiA residues) glycans were identified (hexafucosylated and pentasialylated glycans accounted for ~1% and ~6%, respectively). Hexasialylated and heavily sialylated (≥7 SiA residues) glycans were not detected in these experiments, confirming that these peculiar glycans are present in total plasma at extremely low abundance levels since they were only detected using larger plasma-equivalent volumes. The injection of ~80 pL of plasma-equivalent volume led to the detection of ∼62 glycans, among which ~3% were pentasialylated glycans. However, highly fucosylated glycans (≥4 fucose residues) were not detected in these experiments using sub-0.1 nL plasma volumes (Fig. 2H), and the acquired MS signal intensity levels and the number of assigned glycans expectedly demonstrated lower reproducibility between technical replicates due to the extremely low sample volumes injected. To confirm that the levels of carryover derived from the analysis of preceding plasma-released samples were unsignificant and did not introduce any bias in the subsequent CZE-MS analysis of sub-nL volumes of whole plasma, negative control CZE-MS analyses were systematically performed with a blank sample of water. As shown in Fig 2FI, the analysis of a blank sample did not result in the detection and assignment of N-glycans and allowed us to ensure the reliability and accuracy of the number of glycans identified using sub-1 nL analyses of plasma-equivalents. Finally, compared to recent studies reporting highly sensitive N-glycan profiling of human plasma^49^ or serum^16,52^, the number of identified N-glycans was *increased* > *3-5-fold* and, most importantly, highly fucosylated N-glycans containing up to 6 fucose residues and highly sialylated N-glycans containing up to 8 SiA residues could be detected in human plasma. These glycans were detected in plasma at low or extremely low levels with some abundances being 1000 to 100,000-fold lower than the abundances of the most abundant glycans detected in the total plasma isolate (based on the comparative MS intensity analysis).

The results of the comparative qualitative and quantitative profiling of N-glycans detected and identified in the five sample types (IgG, fetuin, RNase B, EV, and plasma isolates) analyzed in this study are summarized in Fig. 2J. The glycan compositions as well as the determined relative abundances of the glycans identified in the five types of blood- and tissue-derived isolates (Supplementary Data 10) reflect the significant qualitative and quantitative differences of the examined N-glycomes and highlight the uniqueness and complexity of each glycome, with a colossal diversity of glycan structures. For instance, A3G3S3 was detected in each examined biological specimen but at very low abundance in IgG compared to plasma-derived EVs and total plasma. Penta- and hexasialylated glycans A3G3S5 and A3G3S6 were detected only in the fetuin isolate, and the octasialylated glycans detected in the biosamples were specific to each sample type. Overall, these results demonstrate the applicability of the developed CZE-MS method for deep, sensitive, and highly informative N-glycan profiling of minute amounts of biological fluids or tissues, which can be potentially used in clinical applications.

## Discussion

In this study, we developed a CZE-MS method that can be used for sensitive label-free qualitative and quantitative profiling of N-glycans released from proteins isolated from ng-scale complex biomedical samples. N-glycans released from protein isolates derived from blood serum or plasma and pancreas were analyzed in their native non-labeled state, which substantially reduced the extent of desialylation/defucosylation and sample loss, and dramatically simplified the analytical workflow. The combination of DEN-gas, supplied into the region of the nESI interface, with optimized levels of ISCID energy and MS ion transfer tube temperature, improved the detection sensitivity over 45-fold, compared to the conventional mode of instrument operation. CZE-MS migration patterns showed a strong correlation with the structure and charges of the detected glycans and made possible to confirm that ESI- or in-source-induced decay was negligible and did not induce a bias in glycan assignment. Neutral N-glycans that are typically challenging to analyze without derivatization could be detected as well and, as expected, migrated later than sialylated ones.

CZE-MS^2^ in negative ion mode yielded highly informative MS^2^ spectra with extensive cross-ring fragmentation and diagnostic ions that enabled unequivocal identification of antennary structures, core fucose residues, and α-2,3/α-2,6 SiA linkages, and resulted in the successful separation of near-isobaric glycans. Compared to the APTS-labeling approach^35^, the label-free CZE-MS^2^ method generated ~10 times as many structurally informative fragments for underivatized glycans. The analyses conducted without applying the supplemental pressure during the entire time of the CZE-MS run resulted in a ~6-fold increased resolution between closely related glycans and in the separation of positional and linkage isomers. Unmatched separation performance was achieved for α-2,6- and α-2,3-linked SiA isomers. CZE-MS^2^ analysis enabled straightforward and unequivocal assignment of each SiA linkage isomer through the detection of the SiA linkage diagnostic ions. To the best of our knowledge, so far, such differentiation could only be achieved with exoglycosidase-based sequencing^52,75^, a rather tedious, expensive, and time-consuming approach, which may leave some ambiguity in the assignment of glycosidic linkages.

The label-free CZE-MS method we developed resulted in unique and highly specific N-glycosylation profiles for each of the examined proteins, including high-purity model proteins as well as EV and plasma proteins isolated from human plasma. The analysis of the total IgG isolate from human serum resulted in the reliable identification of >250 N-glycans, among which 156 were structurally characterized by MS^2^, using sample amounts equivalent to ~25 ng of glycoprotein (i.e., ~3 nL of serum). The label-free approach resulted in the detection of highly sialylated (up to 5 SiA residues) and highly fucosylated (up to 6 fucose residues) glycans that could not be detected using the APTS-labeling and other reported labeling strategies in equivalent injected amounts of N-glycans. Also, the numbers of highly sialylated (≥4 SiA residues) and highly fucosylated (≥4 fucose residues) glycans detected in this study largely exceed those reported in other glycan profiling studies of human serum IgG^16,38,50,51,76^. These results indicate that the label-free CZE-MS method we developed is gentle for labile monosaccharides. Notably, in human blood, a large variety of factors (number and types of IgG molecules, sequence and PTM variations, cells of origin, high dynamic range, etc.) contribute to the higher heterogeneity of N-glycans in comparison to the majority of more homogeneously glycosylated biopharmaceutical mAbs. Our developed CZE-MS method allowed us to further expand the catalog of blood IgG glycans.

More than 400 N-glycans were identified in the total BSF isolate, and 123 N-glycans were structurally characterized by MS^2^ for injected amounts equivalent to ~5 ng of glycoprotein (i.e., ~10 nL of serum). Highly diverse sialylated N-glycans, with a broad range of sialylation (from mono- to multisialylated (up to 10 SiA residues)) and different types of sialic acids (Neu5Ac and Neu5Gc), were extensively characterized. Detailed structural characterization of unusual and complex heavily sialylated N-glycans carrying up to ten SiA residues was achieved. Such peculiar N-glycans could be included in biopharmaceutical and clinical research and development efforts as novel and unique classes of targets.

The glycan composition profiling of bovine pancreas RNase B isolate resulted in the identification of 150 N-glycans from injected amounts equivalent to ~15 ng of total glycoprotein. High-mannose glycans were detected at high abundance, and the level of sialylation was relatively low compared to the IgG and fetuin isolates. These results demonstrate the potential of the developed CZE-MS method for glycan profiling of proteins with low levels of sialylation.

The analysis of plasma EV isolates and total blood plasma resulted in the detection of 317 and 523 N-glycans from injected amounts equivalent to ∼185 nL and ∼10 nL of plasma, respectively, without applying any derivatization techniques. The high sensitivity and high dynamic range of detection (over 5 orders of magnitude) of the developed CZE-MS method, as well as the preservation of the glycan structures during the sample preparation and the minimal loss of labile monosaccharides during the ESI process, allowed us to detect heavily sialylated (6 to 13 SiA residues) glycans in plasma-derived EVs and total plasma, which were not reported yet using LC- or CE-MS. Moreover, the developed technique allowed us to conduct informative and deep N-glycan profiling injecting samples equivalent to only ~one hundred pL of blood plasma. This minute amount of plasma corresponds to ~6,000 pg of total protein, which is the equivalent to the protein content of ≤10 mammalian cells. These results demonstrated the applicability of the developed method to real-world biological and patient-derived clinical samples, with a sensitivity of detection potentially approaching the single-cell level.

The reported here results demonstrate that the presented CZE-MS method is a powerful qualitative and quantitative approach for deep, sensitive, and highly informative N-glycan profiling of complex biomedical specimens isolated from scarce amounts of physiological fluids (nL- and sub-1 nL-level) or tissues (ng-level). To benchmark the reported numbers of N-glycans for the five types of biological samples analyzed in this study, we performed a systematic and thorough comparison with the literature data. An up to 15-fold increase in the numbers of identified N-glycans was achieved, compared to the techniques previously reported for N-glycan profiling of limited amounts of similar complexity blood isolates. The meticulously processed and annotated MS^2^ spectra across multiple CZE-MS^2^ runs and sample types may become a helpful resource in building an open-source spectral library or for defining pathways for in-silico glycan fragmentation to improve glycan identification workflows and enable novel N-glycan-related studies to address unmet needs in glycan analysis and glycobiology. We believe that the developed label-free technique represents a significant advance in the field of glycomic research and appears as a promising approach for identifying potential glycan biomarkers in cancer or other pathologies using limited amounts of biological or clinical samples (i.e., liquid microbiopsies). The presented approach demonstrates the potential to enable glycan profiling of small cell populations and even single cells. Finally, the most important advantage of our label-free approach is that any sample processing-related analyte alteration is mostly eliminated because the sample preparation is vastly simplified. The labeling efficiency typically varies based on the structure, size and charge of specific glycan species. The same glycan species may yield several species of labeled and unlabeled glycans due to incomplete labeling and partial disintegration of glycans during the process of labeling and sample preparation (i.e., desialylation, defucosylation, etc.). We believe that we could increase the depth of glycan profiling (i.e., detect more glycans) using our label-free approach mostly because we preserve the endogenous glycan structure, especially sialylation and fucosylation, and minimize losses in our simplistic sample preparation protocol. Based on our previous studies and the presented here results, we believe that label-based techniques for N-glycan profiling are incapable of accurately recapitulating the complexity of glycans present in the biological systems due to the substantial sample alteration over the course of sample preparation and derivatization. In this study, we presented an alternative approach, which can preserve and structurally characterize native glycan structures with higher accuracy, precision, and sensitivity.

## Methods

### Human samples

The described research complies with all relevant ethical regulations. The research experiments involving human subjects were reviewed by the respective authorized Institutional Review Boards (IRB) with approvals IRB#2001P000S91 (BIDMC) and IRB#17-12-14 (NU). Twelve self-declared healthy male donors of the age 23–67 years old were selected, and informed consent was obtained for each participant. A compensation of $10 was provided to the donors. No sex or gender analysis was carried out since the comparative analysis of the selected samples was not the goal of this proof-of-concept study.

### Materials and chemicals

Deionized water, methanol (99.9%), and isopropanol (99.9%) were obtained from Fisher Scientific (Waltham, MA). 1 N NaOH, 1 N HCl, 5 N ammonium hydroxide, glacial acetic acid (99.99%), ultra-high-purity ammonium acetate (99.999%), total human serum IgG isolate (purity ≥95%, based on non-reduced SDS-PAGE and verified by nanoLC-MS/MS of tryptic digests), and BSF isolate (purity ≥90%) were purchased from Sigma-Aldrich (St. Louis, MO). Agencourt Cleanseq carboxyl-coated magnetic microparticles (COOH-beads) were from SCIEX (Brea, CA). PNGase F enzyme and bovine pancreas ribonuclease B isolate (purity ≥85%, based on SDS-PAGE) were from New England Biolabs (Ipswich, MA). A neodymium magnet was obtained from K&J Magnetics (Pipersville, PA). All bare-fused silica (BFS) capillaries (91 cm × 30 µm i.d. × 150 µm o.d.) with sheathless CESI-MS emitters in OptiMS cartridges were from SCIEX. A NanoBooster^TM^ unit for generating the DEN-gas was from Bruker Daltonics (Billerica, MA).

### Sample preparation

The protocol to prepare all of the N-glycans analyzed in this study was adapted from the fast glycan kit protocol developed by^77,78^ and commercialized by SCIEX. In brief, 200 µL of the magnetic bead suspension were pipetted into a 0.6 mL PCR tube. Then, the magnetic beads were pulled to the side of the tube using the neodymium magnet, and the storage solution was carefully collected. 10 µL of glycoprotein isolates (human serum IgG, BSF, or bovine pancreas ribonuclease B) at 5 mg/mL or 50 µL of blood-derived EV or plasma isolates were loaded on the beads. The mixture was vortexed for 10 s. 5 µL of denaturation solution (SCIEX) were then added to the sample tube, placed on a heating block, and thermostated at 60 °C. After the protein denaturation step (8 min), 12 µL of digestion solution containing 5 mU of PNGase F enzyme were added to the sample tube, and the incubation was performed at 60 °C for 20 min with the sample tube open. After the deglycosylation step, 200 µL of acetonitrile were added to the sample tube for glycan capture. The supernatant (containing the deglycosylated proteins) was then removed from the tube by pulling the magnetic beads to the side. Finally, the N-glycans were eluted from the beads by the addition of 100 µL of water. For IgG, the concentration of the released N-glycans was assumed to be ≤10 µg/mL (based on a rough estimate that glycosylation accounts for ~2% of the molecular mass of IgG^14^). N-glycans were analyzed by CZE-MS in their native non-labeled state.

### CZE methods

CZE-MS experiments were conducted using a CESI 8000 instrument (SCIEX). In all experiments, bare-fused silica (BFS) OptiMS capillaries (91 cm × 30 µm i.d. × 150 µm o.d.) were used. No capillary surface adsorption-related phenomena resulting in characteristic peak tailing were observed for non-labeled N-glycans using these uncoated capillaries. Sample injections were performed at 1, 3, or 5 psi for 60 s, corresponding to 10, 31, and 51 nL injection volumes, respectively (i.e., 1.6, 4.8, and 7.9% of the capillary volume, respectively) for standard model glycoproteins. Approximately 7 nL and 2–5 nL of samples were injected for total EV isolate and total plasma, respectively. Prior to each injection, a series of rinses of the separation and conductive lines were performed. For the separation capillary, these rinses included: 0.1 M HCl (100 psi, 3 min), Milli-Q water (100 psi, 5 min), followed by the BGE (100 psi, 10 min). The conductive line was rinsed with the BGE (75 psi, 4 min). In each CZE-MS analysis of released N-glycans, sample amounts equivalent to ~25 ng (human serum IgG), ~0.5 or 5 ng (BSF), and ~15 ng (ribonuclease B) of glycoproteins were injected, which correspond to ~3 nL of human serum for IgG, and ~1 or 10 nL of bovine serum for fetuin.

All CZE methods employed 20 kV in reverse polarity with voltage ramp times of 1 min. The experiments were carried out with a BGE of 10 mM (ionic strength) ammonium acetate pH 4.5 with 10% isopropanol. This BGE generated a relatively low cathodic EOF (µ_EOF_ 2.02 × 10^−8^ m^2^/V/s) based on the detection of a neutral marker, acetaminophen. The CZE analyses were performed with or without a supplemental pressure (SP) equal to or less than 5 psi, and the analysis times varied from 48 to 78 min depending on the applied SP. For profiling of glycan compositions (i.e., the number and types of monosaccharides in a given oligosaccharide) and glycan structures (i.e., exact monosaccharide arrangements and interconnecting glycosidic linkages in the oligosaccharide), the CZE SP was switched off for 18 min before applying 5 psi during the rest of the analysis. For CZE-MS method optimization, at least three replicate experiments were performed under the selected analytical conditions. The optimized method used DEN-gas with IPA combined with ITT at 150 °C and ISCID at 70 eV.

### MS instrumentation and techniques

Q Exactive^TM^ Plus Orbitrap^TM^ MS (Thermo Fisher Scientific, Bremen, Germany) was used. All analyses were carried out in negative ESI mode. The nanoelectrospray potential was set to −1.4 kV. The ion transfer tube (ITT) temperature was set to 150 °C (the distance between the electrospray emitter and the MS ITT was set to ∼5 mm). The CZE-MS analyses were performed with automatic gain control (AGC) of 10^6^, a maximum injection time of 250 ms, 5 microscans, a S-lens voltage set to 65 eV, the nominal resolving power of 70,000 at 200 Th, and in-source collision-induced dissociation (ISCID) at 70 eV. For CZE-MS^2^ experiments, a “Top 15” data dependent acquisition method was used. CZE-MS was performed as described above. For MS^2^ scans, instrument resolving power was set at 35,000 at 200 Th with 1 microscan. AGC was set to 2 ×10^5^ with a maximum injection time of 150 ms. An isolation window of 2 Th was selected, and 32 eV was determined to provide the optimum normalized collision energy. The DEN-gas supply line was connected to the commercial sheathless CZE-ESI-MS system via a Teflon tube with an outlet placed in close proximity to the electrospray emitter (∼2 cm) to saturate the space around the ESI emitter with the DEN-gas. 100% IPA was used as a dopant with a pressure of supplied nitrogen gas set to ~3–5 psi.

### Data analysis

For data acquisition and processing, Xcalibur^TM^ (v. 2.8) software was used. CZE-MS data were processed with GlycReSoft (v. 3.10) software (Boston University, Boston, MA, USA)^79^. Analyses of CZE-MS^2^ data were performed with SimGlycan (v. 5.91) software (Premier Biosoft, Palo Alto, CA, USA). The generated results were based on the processing of three replicate analyses. The glycan composition identification results were mainly based on CZE-MS data processing using GlycReSoft. As additional verification of the plausible glycan identifications made using GlycReSoft, several supplementary levels of manual data examination were applied according to our recent study^35^. In brief, this verification included 1. CZE-MS migration patterns (predictable trends in CE migration based on net charge and hydrodynamic volume, and *m/z* shifts governed by monosaccharide mass increments), 2. charge state and isotopic distributions characteristic to glycan ions, 3. the detection of neutral losses (e.g., GlcNAc, hexose, and fucose), and 4. the manual examination of CZE-MS^2^ data for low intensity parent ions identified using GlycReSoft to confirm if the MS^2^ spectral patterns were characteristic of glycan fragmentation even if the MS^2^ spectra did not result in positive identifications using SimGlycan (see Supplementary Methods for more details). The relative quantitation of the detected N-glycans was based on the single-stage MS signal intensities of the detected N-glycans that were normalized with respect to the summed MS signal intensities of all the N-glycans detected in the sample. In addition, a qualitative comparison was performed based on the fractional distributions corresponding to the number of specific species (e.g., monofucosylated glycans) out of the total number of N-glycans detected and identified in the analyzed blood or tissue isolates.

Additional experimental details in accordance with the MIRAGE guidelines^80^ are provided in the Supplementary Information. The generated raw data were deposited in GlycoPOST, a dedicated repository for MS-based glycomics^81^. The glycan structures were designed with the open-access version of GlycoWorkBench and a schematic representation of IgG was designed with ChemDraw software (v.22.0.0).

### Reporting summary

Further information on research design is available in the Nature Portfolio Reporting Summary linked to this article.

## Supplementary information

Supplementary Information

Description of Additional Supplementary Files

Supplementary Data 1

Supplementary Data 2

Supplementary Data 3

Supplementary Data 4

Supplementary Data 5

Supplementary Data 6

Supplementary Data 7

Supplementary Data 8

Supplementary Data 9

Supplementary Data 10

Reporting Summary

## Data Availability

The raw data generated in this study have been deposited in GlycoPOST (https://glycopost.glycosmos.org) under the accession numbers GPST000122 and GPST000281. Source data are provided with this paper.

## Source data

Source Data
