# Supplementary material for: Highly-sensitive label-free deep profiling of N-glycans released from biomedically-relevant samples
Source: Nat Commun. 2023 Mar 23;14:1618. doi: 10.1038/s41467-023-37365-4 (PMC10036494; doi:10.1038/s41467-023-37365-4)
Supplement: Supplementary file 1 — Supplementary Information [file 41467_2023_37365_MOESM1_ESM.pdf]

Supplementary Materials for

Highly-sensitive label-free deep profiling of N-glycans released from biomedically-relevant samples

Anne-Lise Marie[1], Somak Ray[1], and Alexander R. Ivanov[1*]

* Corresponding author. E-mail: a.ivanov@northeastern.edu
Phone: +1 617 373 6549

Supplementary Methods

Supplementary Notes 1-10

Supplementary Figures 1-27

Supplementary References

Supplementary Methods

Preparation and characterization of EV isolate. The EV isolate was prepared from ~2.5 mL of platelet-free anticoagulated with EDTA pooled human blood plasma (from blood donated by healthy male donors of 23-67 years old) using ultracentrifugation and differential centrifugation, as described in previously published work [1]. The purity of the EV isolate was ≥ 90-95%, based on a combination of EV counting, using tunable resistive pulse sensing (TRPS), nanoparticle tracking analysis (NTA) or nano-flow cytometry, and protein concentration measurements, using UV-spectrophotometry, fluorescence, and BCA assay [2]. The validation of the purity of the EV isolate was performed as described in our previous work [3].

Data analysis. For data acquisition and processing, Xcalibur™ (v. 2.8) software was used. CZE-MS data were processed with GlycReSoft (v. 3.10) software (Boston University, Boston, MA, USA) [4]. Analyses of CZE-MS^2 data were performed with SimGlycan (v. 5.91) software (Premier Biosoft, Palo Alto, CA, USA). The generated results were based on the processing of three replicate analyses. The glycan composition identification results were mainly based on CZE-MS data processing using GlycReSoft. As additional verification of the plausible glycan identifications made using GlycReSoft, several supplementary levels of manual data examination were applied according to our recent study [3]. In brief, this verification included 1. CZE-MS migration patterns (predictable trends in CE migration based on net charge and hydrodynamic volume, and *m/z* shifts governed by monosaccharide mass increments), 2. charge state and isotopic distributions characteristic to glycan ions, 3. the detection of neutral losses (e.g., GlcNAc, hexose, and fucose), and 4. the manual examination of CZE-MS^2 data for low intensity parent ions identified using GlycReSoft to confirm if the MS^2 spectral patterns were characteristic of glycan fragmentation even if the MS^2 spectra did not result in positive identifications using SimGlycan.

In GlycReSoft, a mass matching error tolerance of 20 ppm was used in all searches. A background reduction factor of 5 was selected to remove the low abundance signal in MS^1 scans. Different charge states (up to 4) and ammonium and sodium adducts were included in the search to eliminate redundant identifications, so that only one single glycan composition was provided for a glycan species that could be ionized at different charge states or associated with adduct ions. Only glycan compositions provided with a score ≥8 were selected according to the recommendations of the program developers and manual validation. The glycan identification analysis of the CZE-MS data was conducted using database searches against human and mammalian databases of N-glycans provided with the software package.

For CZE-MS^2 processing with SimGlycan, a 20 ppm precursor mass tolerance and a 10 ppm fragment mass tolerance were used in all searches. Negative ion mode was set, and for adduct formation, "H, NH_3, and Na" were chosen. For the search of non-labeled glycans, the options "Underivatized" and "Free" were selected in the chemical derivatization and reducing terminal windows, respectively. The research filters encompassed glycoproteins and N-glycans (intact core). Only the glycan compositions and structures provided with a proximity score of ≥60% were selected. Besides this search engine-assisted annotation, more than 30 MS^2 spectra that resulted in positive structural identification (scored above the selected threshold of 60) were analyzed manually to 1. confirm the structural characterization results provided by the software, 2. identify

diagnostic ions for the assignment of SiA linkages and antenna branching, and 3. assign the fragment ions derived from multiple internal fragmentation cleavages.

For data clustering, the observed abundance of each glycan detected in the analyzed sample (IgG, BSF, RNase B, EV or plasma isolates) was normalized with respect to the summed abundances of all the glycans detected in the sample. Hierarchical clustering based on the Euclidean distance measure and the complete linkage algorithm was done using the normalized intensity values after imputing 10% of minimum intensity for missing values followed by log2 transformation. The R (version 4.05) packages "pheatmap" (version 1.0.12) and "circlize" (version 0.4.14) were used respectively to generate linear and circular clustered heatmaps.

The bar charts with individual data points, mean values and error bars were plotted with R statistical analysis software (version 4.2.2).

Supplementary Note 1

Separation of neutral N-glycans. The hypothesis that the endogenous neutral glycans detected and separated with our CZE-MS method got mobilized under the applied CZE conditions through ion-dipole intermolecular interactions with anions present in the BGE, presumably acetate anions, was supported by the following results and observations:

1- In our CZE-MS conditions where the supplemental CZE pressure is switched off for 18 min before applying a CZE voltage, the neutral analyte molecules present in the sample plug migrate toward the inlet at the beginning of the CZE-MS run driven by the EOF and should reach the inlet in less than 3 min after turning on the voltage, which would make their detection impossible.

2- Experiments performed by applying a continuous pressure of 5 psi over the all duration of the CZE-MS run resulted in the detection of endogenous neutral glycans, with migration times lower than the migration time of the neutral marker (acetaminophen) that was used to measure the EOF, and higher than the migration times of the sialylated glycans.

3- A strong correlation was observed between the CZE migration order of the endogenous neutral N-glycans and their respective monosaccharide composition, which determines their molecular mass as well as their hydrodynamic volume.

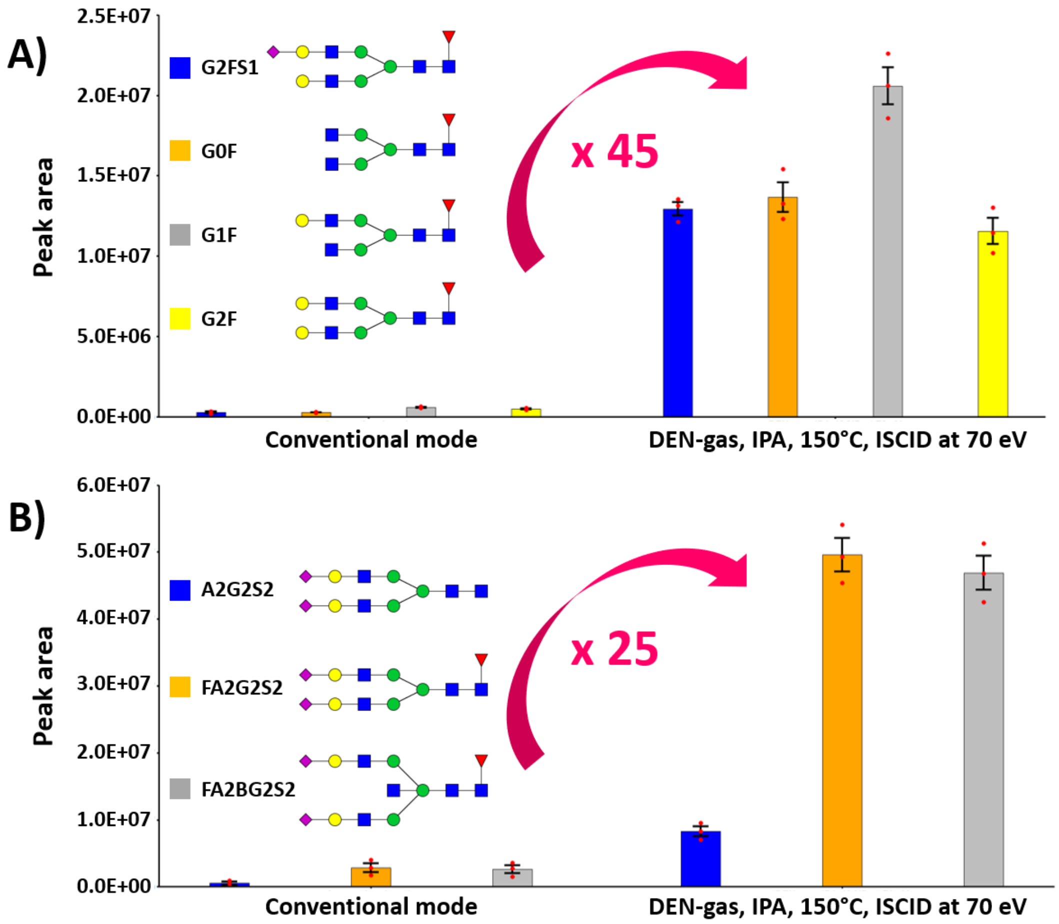

Supplementary Figure 1: The effect of experimental conditions on peak areas of selected complex-type N-glycans in CZE-MS-based profiling of IgG isolate. CZE-MS analyses (n=3 technical replicates, data are presented as mean values ± SD, red dots correspond to individual data points) of human serum IgG were conducted in two different conditions: 1. conventional mode of instrument operation (ITT at 110°C); and 2. nitrogen gas enriched with IPA (ITT at 150°C) and ISCID at 70 eV. Neutral and monosialylated N-glycans (A), and disialylated glycans (B) were selected as representative examples of the sensitivity improvement.

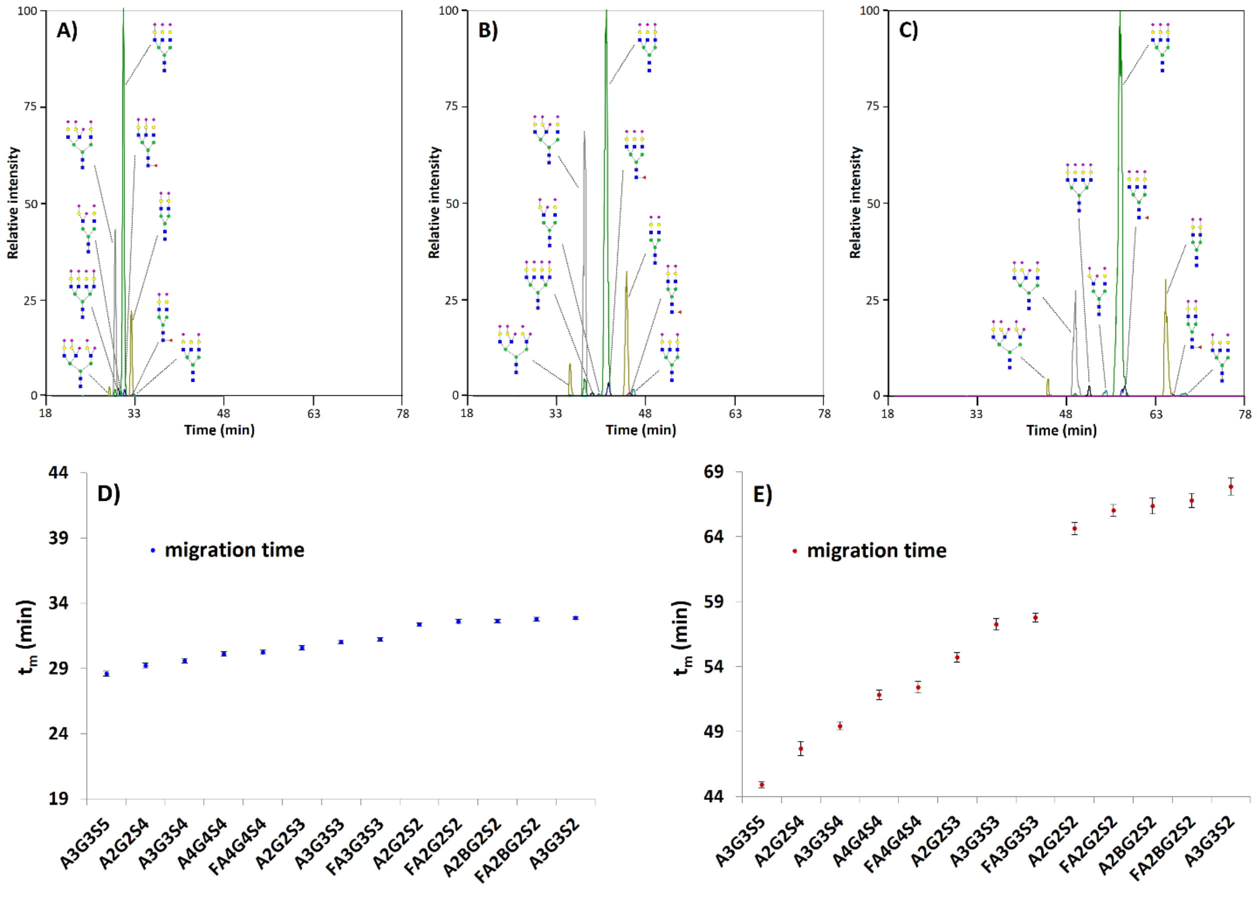

Supplementary Figure 2: Improvement of the CZE resolution of N-glycans with a decreased supplemental pressure (SP). A-C) Extracted ion electropherograms (EIEs) of nine representative sialylated N-glycans acquired from the CZE-MS analysis of fetuin-derived N-glycans using a SP of A) 5 psi, B) 2 psi, and C) 0 psi. The SP was applied at the inlet of the CE capillary 18 min after the beginning of the CZE-MS run. D-E) Migration times (t_m) of thirteen selected fetuin-derived N-glycans as determined with three successive CZE-MS analyses using a SP of A) 5 psi, and B) 0 psi, applied 18 min after the beginning of the CZE-MS run (n=3 technical replicates, data are presented as mean values ± SD).

Supplementary Note 2

To evaluate the repeatability of the developed CZE-MS method, four successive analyses of fetuin-derived N-glycans were performed the same day on the same capillary, and the migration times and peak areas of eight selected N-glycans with various degrees of sialylation and fucosylation were measured (**Supplementary Figure 3**). The intra-day repeatability of the method was appropriate for the CZE-based technique, where RSDs were less than 0.7% and 13% for migration times and peak areas, respectively. An inter-day comparison (three replicates on three different days on the same capillary) provided RSDs less than 1.0% and 15% for migration times and peak areas, respectively, for the eight selected N-glycans.

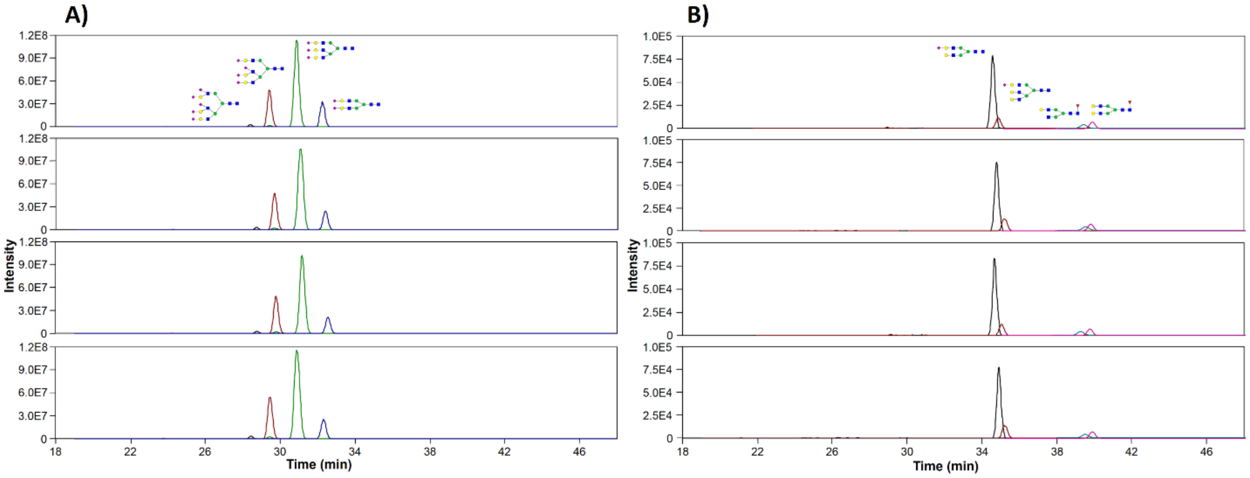

Supplementary Figure 3: Assessment of the CZE-MS method repeatability with four successive CZE-MS analyses of fetuin-derived N-glycans. EIEs of eight selected high and low abundance N-glycans. A) EIEs of four sialylated N-glycans. B) EIEs of two monosialylated and two neutral N-glycans.

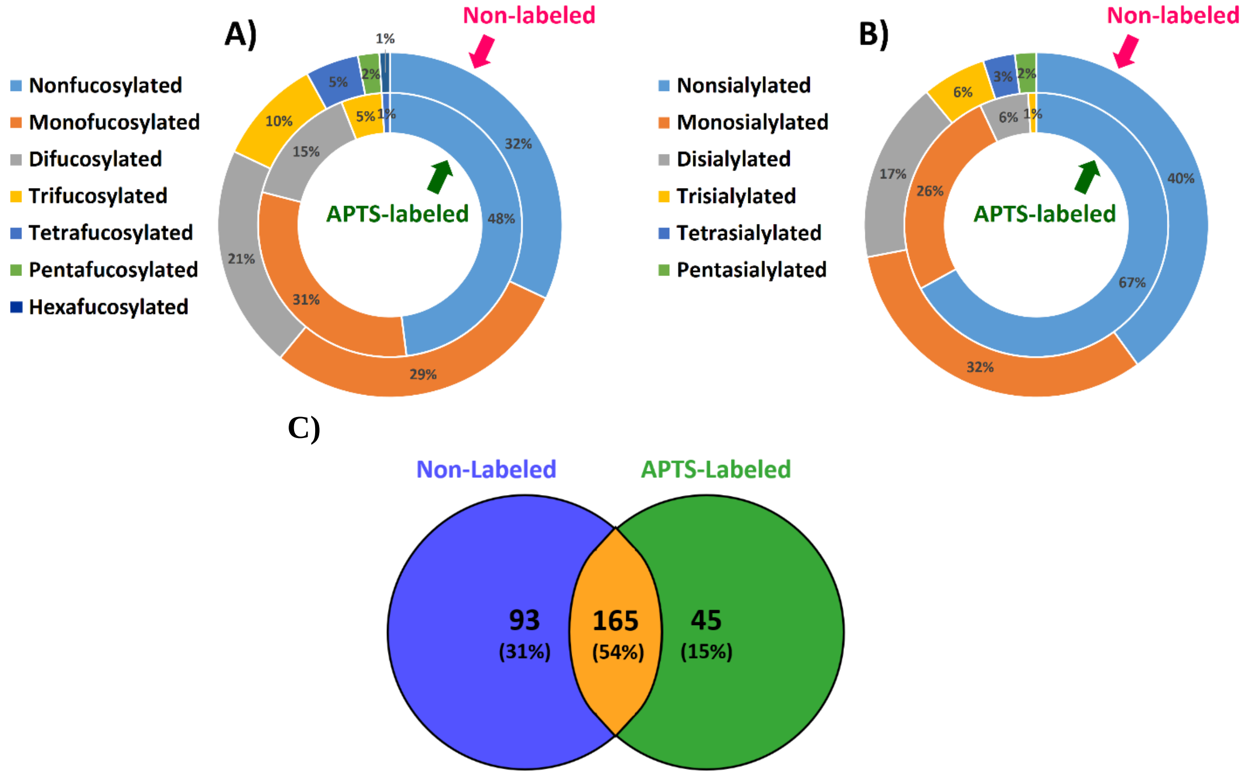

D)

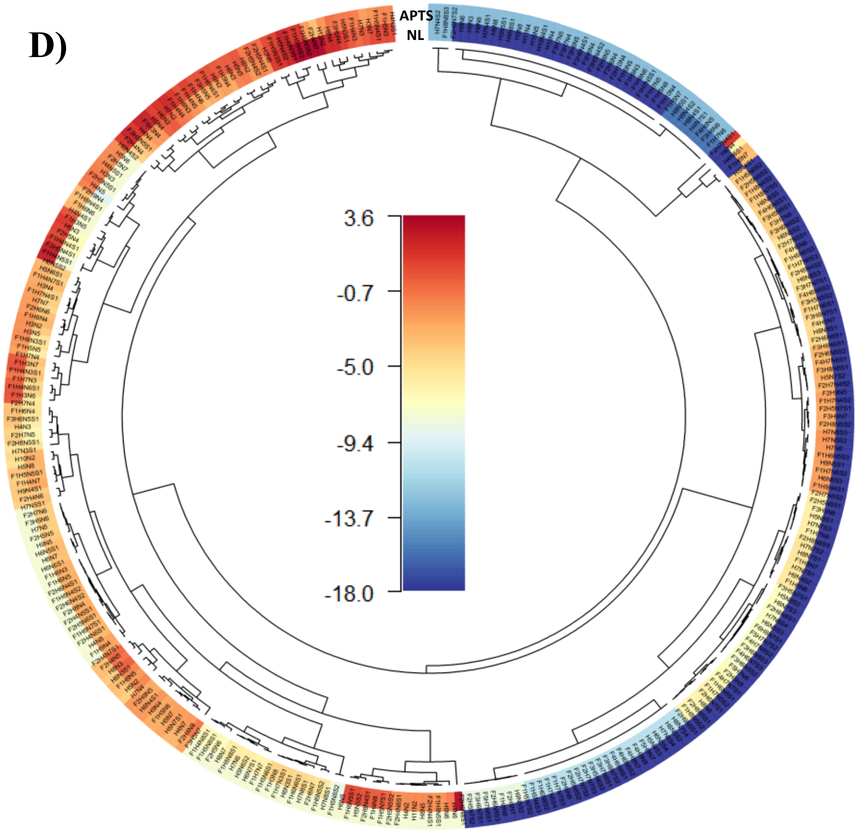

E)

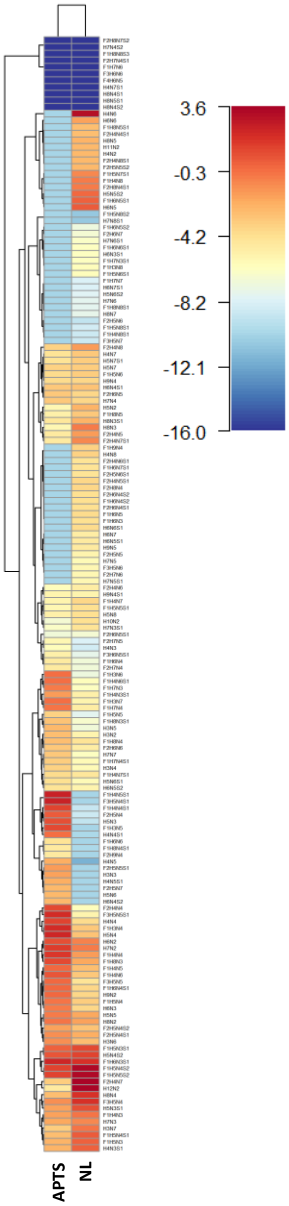

F)

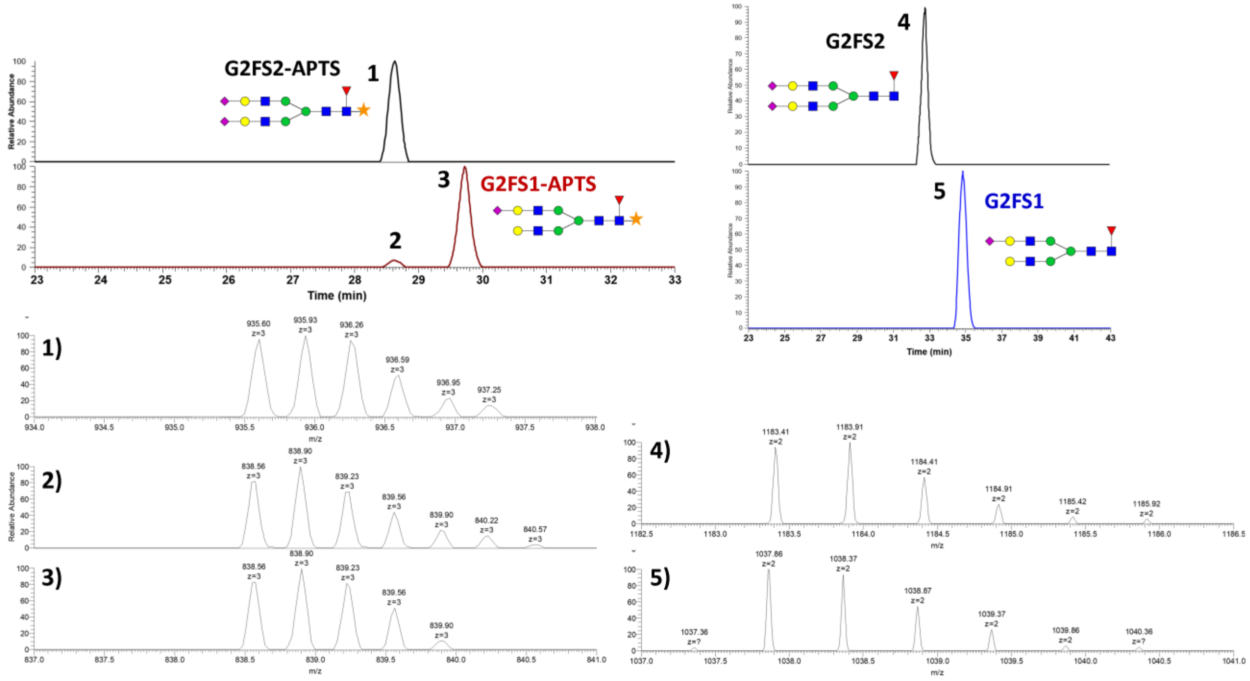

Supplementary Figure 4: Comparison of label-free and APTS-labeling approaches in the CZE-MS-based N-glycan profiling of human serum IgG. Fractional distributions of fucosylated **(A)** and sialylated **(B)** N-glycans detected in the human serum IgG isolate. The label-free CZE-MS method enables to detect highly fucosylated (≥5 fucose residues) and highly sialylated (≥4 SiA residues) glycans that are not detected using the APTS-labeling-based CZE-MS method [3]. As shown in the Venn diagram in panel **C**, the non-labeling strategy results in the detection of 93 additional and unique N-glycans that are not detected with APTS labeling. Panels **D-E** show the Euclidean-based hierarchical clustering of quantitative profiles of N-glycan detected in human serum IgG with the APTS-labeling and label-free CZE-MS methods, respectively, using injected sample amounts equivalent to ~3 nL of serum (NL stands for non-labeled). The glycans commonly detected using both strategies are shown on panel **E**. Red, yellow, and light blue colors correspond to high, medium, and low relative abundances based on the N-glycan signal intensities. N-glycans that are not detected in the samples are highlighted in dark blue. F, H, N, and S stand for fucose, hexose, HexNAc, and Neu5Ac, respectively. **F)** Characteristic examples of in-source decay fragmentation of APTS-labeled glycans. On the left-hand side is shown in-source fragmentation of the APTS-labeled disialylated glycan G2FS2, which results in the loss of one sialic acid residue. No detectable in-source decay fragmentation was observed when the same disialylated glycan was analyzed in its native non-labeled state, as shown on the right-hand side of panel F (selected *m/z*: 935.59 (G2FS2-APTS), 838.56 (G2FS1-APTS), 1,183.41 (G2FS2), and 1,037.86 (G2FS1), with a mass tolerance of 20 ppm).

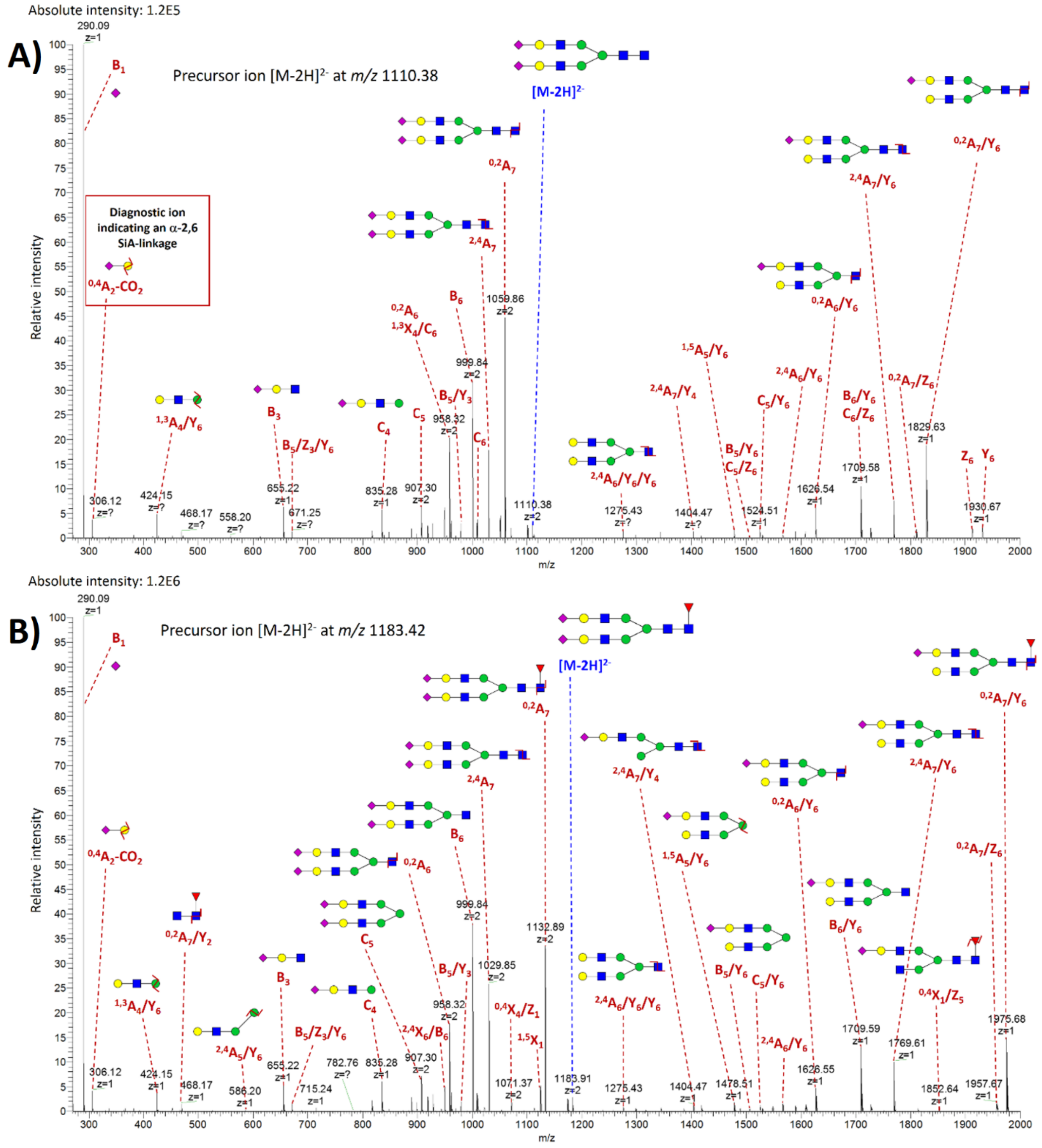

Supplementary Figure 5: Examples of MS^2 spectra of closely-related glycan species **A2G2S2** (A), and **FA2G2S2** (B). The MS^2-based structural characterization was performed selecting the $[M-2H]^{2-}$ molecular ions at *m/z* 1,110.38, and 1,183.42, respectively, as precursor ions. Fragment ions are annotated based on the Domon and Costello nomenclature. Blue square, GlcNAc; red triangle, Fuc; green circle, Man; yellow circle, Gal; purple diamond, Neu5Ac. Symbol Z indicates cross-ring fragmentation. Only the most intense/relevant fragments are annotated in the shown spectra.

A)

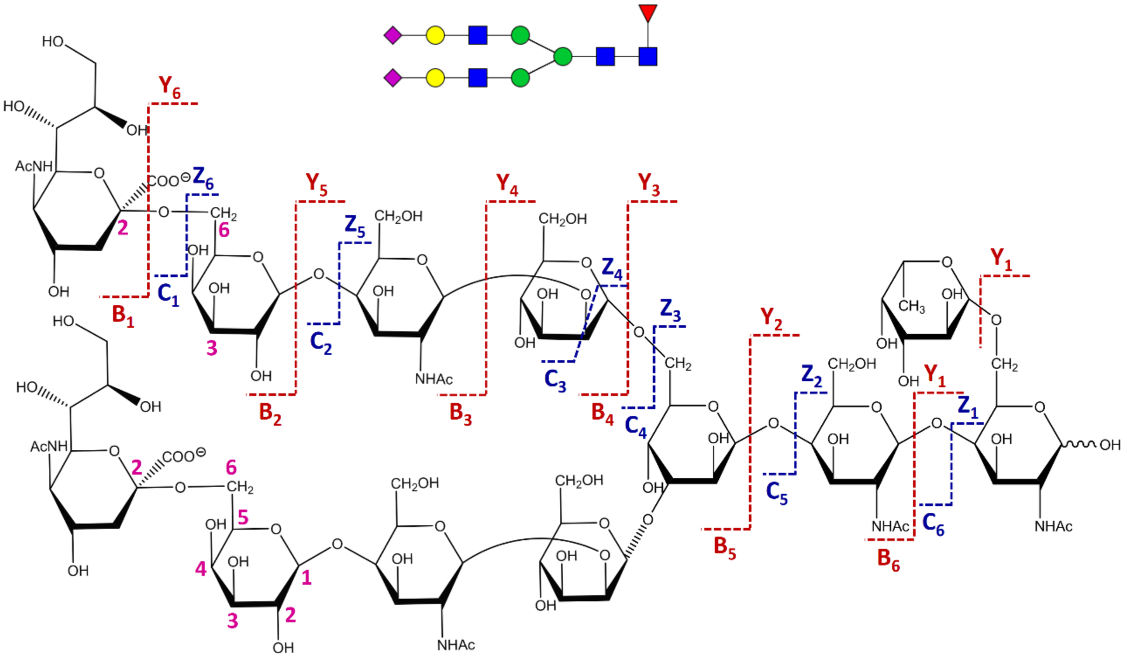

B)

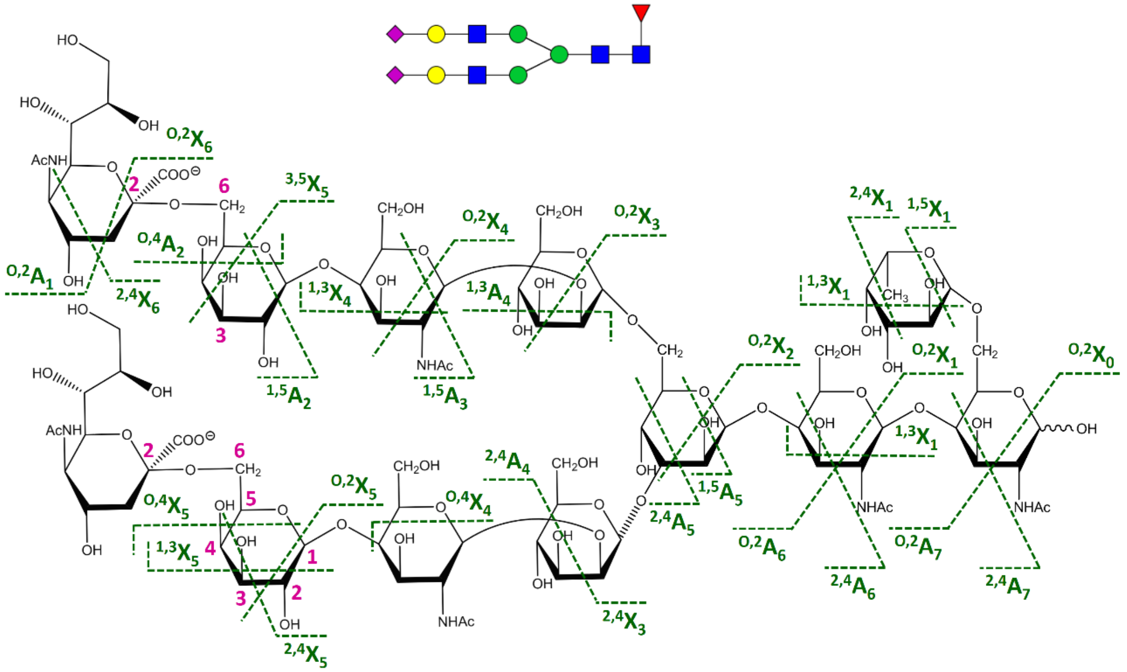

Supplementary Figure 6: ***In-silico*** **predicted fragmentation cleavages** in negative ion mode of the bi-antennary fucosylated glycan **FA2G2S2** with α-2.6-linked sialic acid residues. A) Glycosidic and glycosidic/glycosidic *in-silico* predicted fragments. B) Single cross-ring, and cross-ring/glycosidic *in-silico* predicted fragments. The $^{0,4}A_2$ fragment ion enables the assignment of α-2.6 SiA linkage.

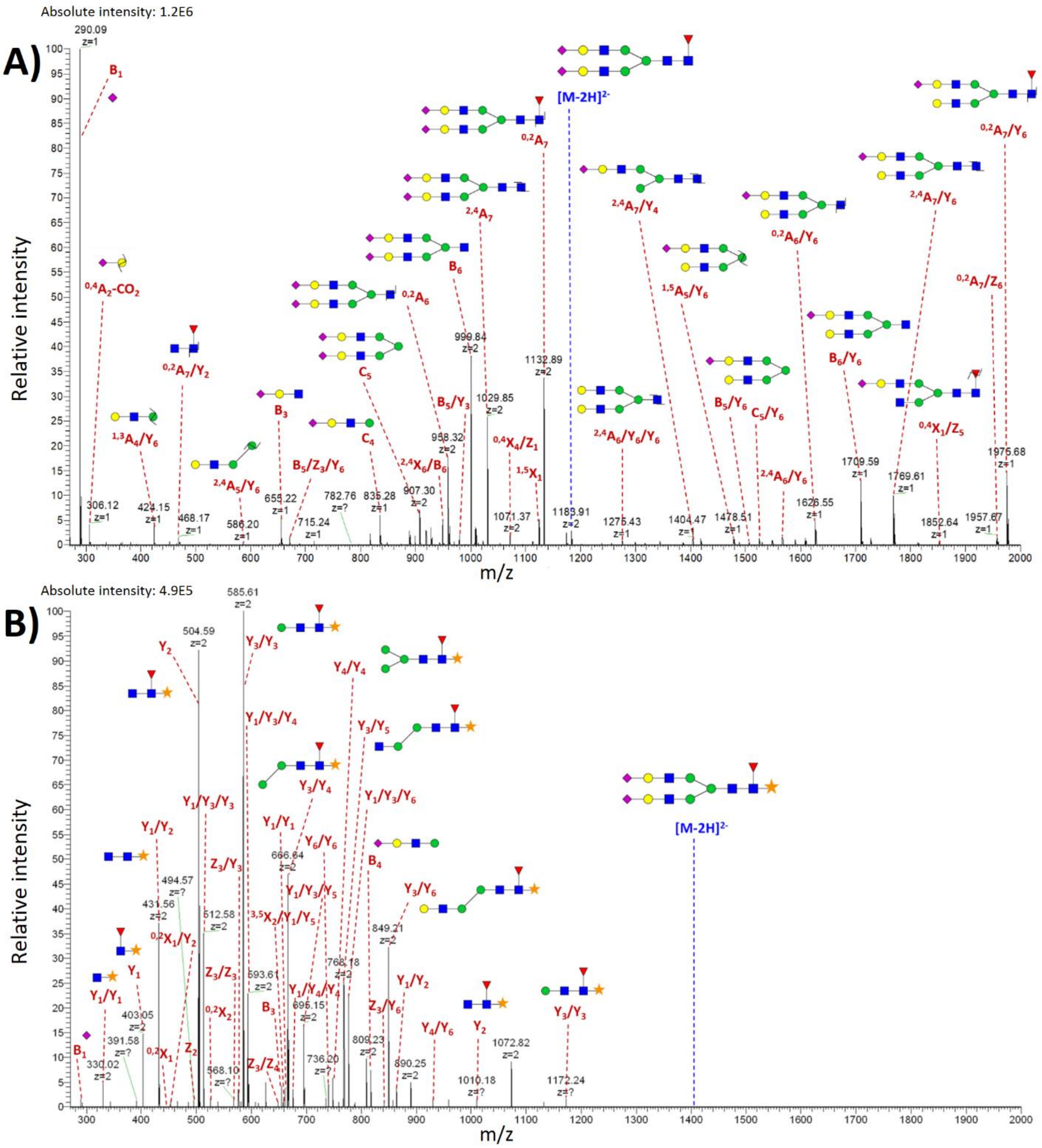

Supplementary Figure 7: Examples of the characteristic MS^2 spectra in negative ion mode of FA2G2S2 without (A) or with (B) APTS-labeling. Cross-ring and cross-ring/glycosidic fragment ions are predominant in the mass spectra of **non-labeled FA2G2S2**, whereas glycosidic and glycosidic/glycosidic fragment ions dominate the mass spectra of **APTS-labeled FA2G2S2**. Symbol *Z* relates to cross-ring fragmentation. The orange star indicates the APTS label. See Supplementary Figure 5 for other symbols.

Supplementary Note 3

For MS^2 structural characterization of **FA2BG2S2**, the $[M\text{-}2H]^{2-}$ molecular ion at *m/z* 1,284.95 was selected as a precursor ion (**Supplementary Figure 8**). The presence of one additional GlcNAc residue was supported by the shift of Z_6^{1-} and Y_6^{1-} fragment ions, detected at *m/z* 2,261.79 and 2,279.81, respectively, compared to the non-bisecting fucosylated analog, but also with the shift of B_6^{2-} and C_6^{2-} ions, detected at *m/z* 1,101.38 and 1,110.38, respectively, compared to the non-bisecting fucosylated and unfucosylated analogs. The bisecting nature of the additional GlcNAc residue was clearly indicated by the neutral loss of one GlcNAc moiety (221.09 Da) from the $B_5/Y_{3\alpha}^{1-}$ ion (*m/z* 1182.40), as illustrated by the pair of singly-charged ions at *m/z* 220.08 and 961.31 [5]. The mass difference of 206.08 Da between the two pairs of ions ${}^{2,4}A_7^{2-}$ (*m/z* 1,131.39) and ${}^{0,2}A_7^{2-}$ (*m/z* 1,234.43), and ${}^{2,4}A_7/Y_6^{1-}$ (*m/z* 1,972.68) and ${}^{0,2}A_7/Y_6^{1-}$ (*m/z* 2,178.76) located the fucose on the chitobiose core. In addition, α-2,6 SiA linkages were documented with the presence of ${}^{0,4}A_2\text{-}CO_2^{1-}$ diagnostic ion at *m/z* 306.12.

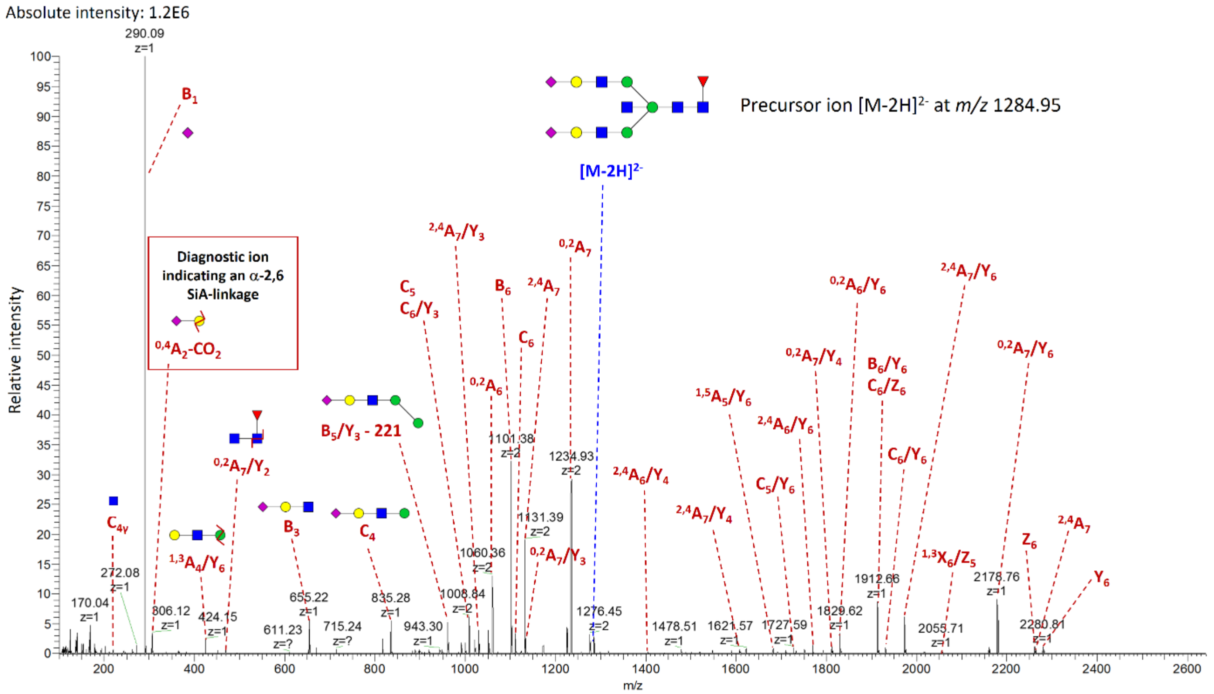

Supplementary Figure 8: An example of the characteristic MS^2 spectrum of the bisected N-glycan **FA2BG2S2**. The $[M\text{-}2H]^{2-}$ molecular ion at *m/z* 1,284.95 was selected as a precursor ion for MS^2-based structural characterization.

Supplementary Note 4

Neutral N-glycans like **FA2G1** (G1F) and **FA2G2** (G2F) are known to be present at high abundance in serum IgG and have been extensively described in the literature [6,7]. **Supplementary Figure 9B** depicts the fragmentation pattern of FA2G2 (Mr_{th} 1,786.65 Da). For this glycan, the $[M\text{-}2H]^{2-}$ molecular ion at *m/z* 892.32 was selected as a precursor ion. The fragment ions C_1^{1-} (*m/z* 179.05) and ${}^{1,3}A_3^{1-}$ (*m/z* 424.14), detected at high intensity, enabled us to allocate the galactose

residues at the termini of the antennae. The mass spectra of FA2G2 also exhibited a prominent $^{2,4}A_6/Y_2^{1-}$ (*m/z* 262.09) fragment ion, corresponding to the cleavage of the chitobiose core. The MS^2 spectra of a structurally-related species, **FA3G1** (Mr_{th} 1,827.68 Da), were also dominated by $^{2,4}A_6$ fragment ions, with or without internal fragmentation (**Supplementary Figure 9C**). The CZE-MS analyses also allowed us to detect neutral glycans rarely reported in human IgG, including the tetra-antennary bisecting glycan **FA4F1BG1**, with one core fucose and one outer arm fucose (**Supplementary Figure 9D**).

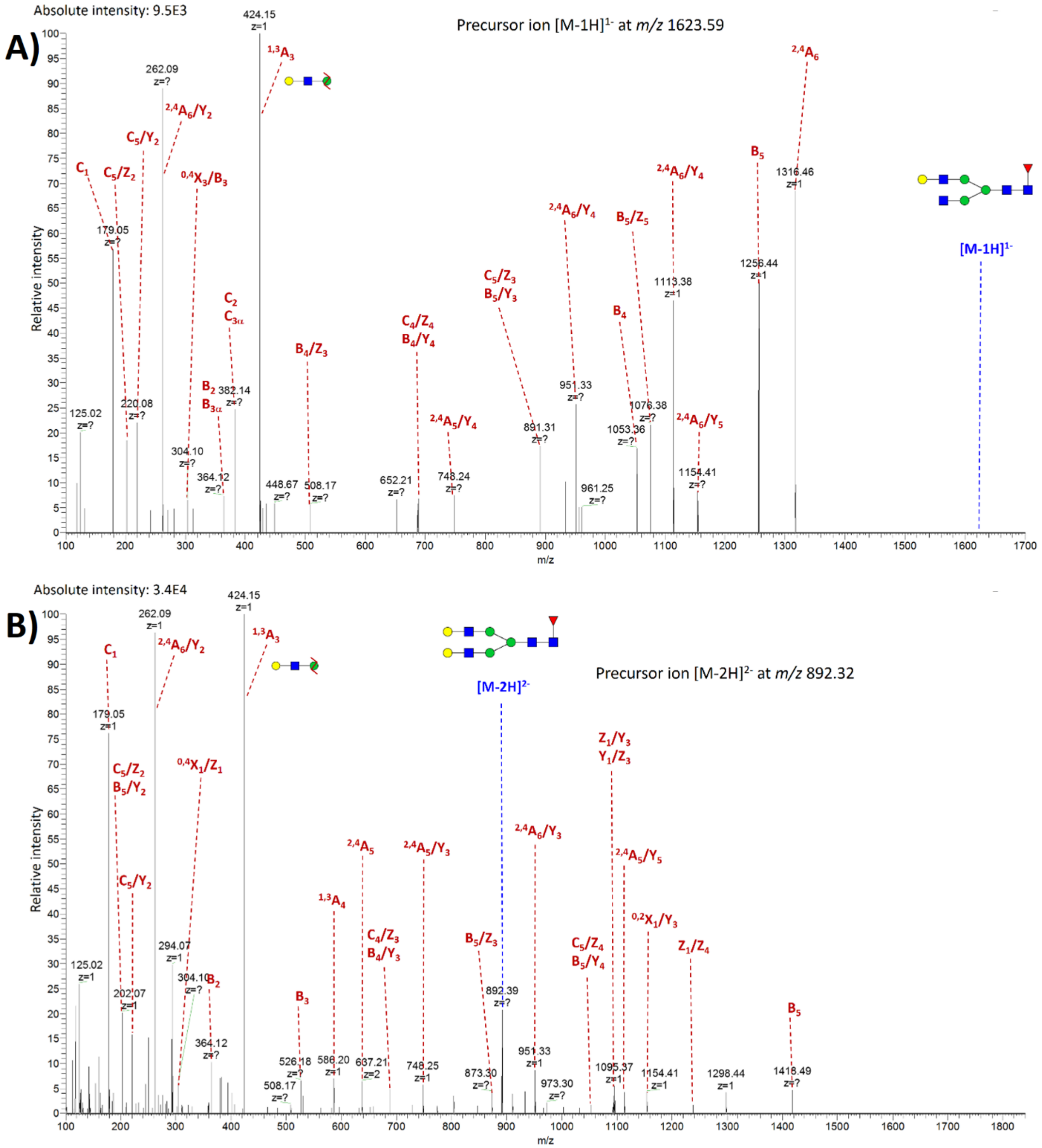

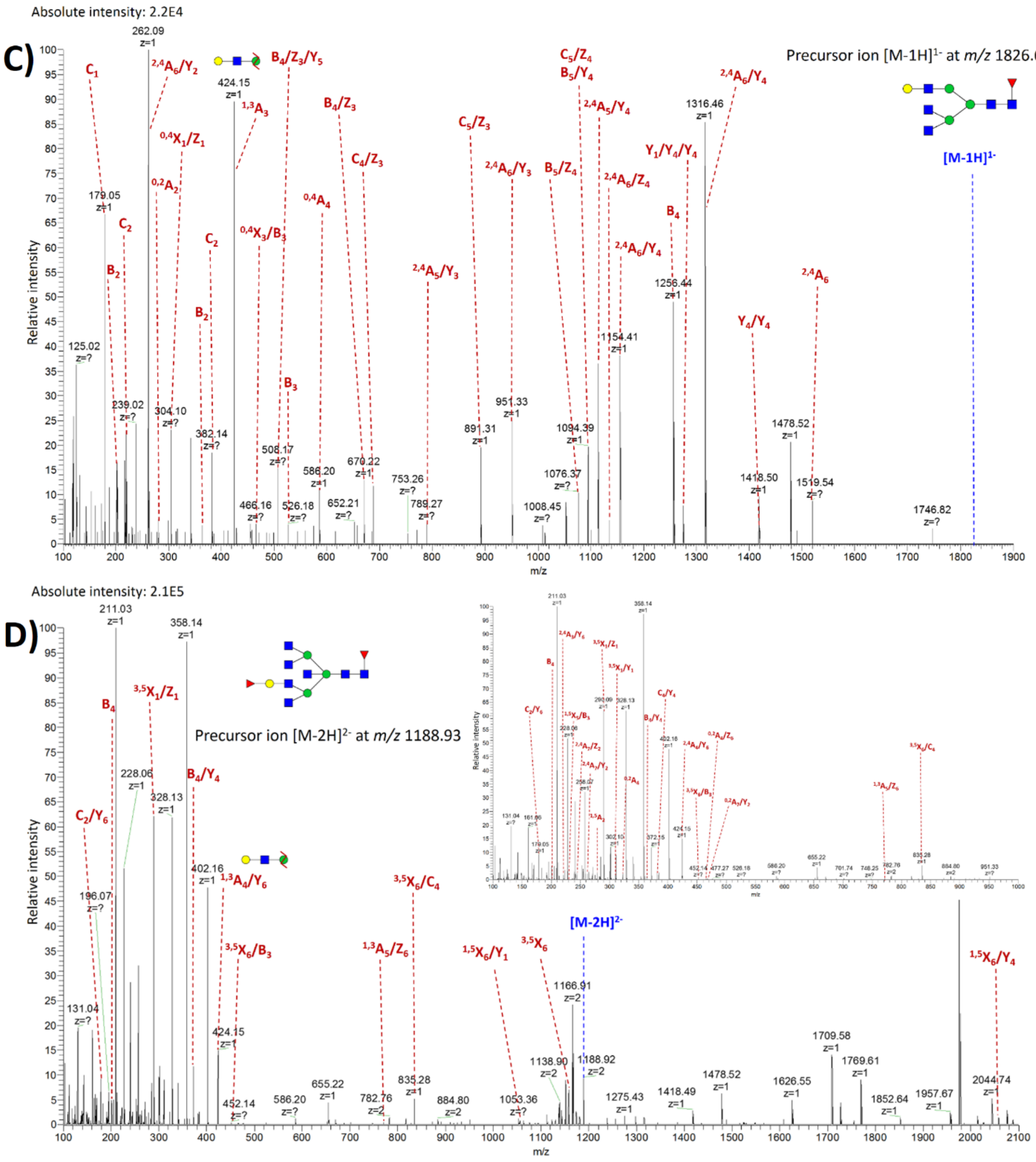

Supplementary Figure 9: Examples of characteristic MS^2 spectra of non-labeled **neutral N-glycans** detected in the IgG isolate: A) **FA2G1**, B) **FA2G2**, C) **FA3G1**, and D) **FA4F1BG1**. For A) and B), the singly-charged $[M\text{-}1H]^{1-}$ molecular ions at *m/z* 1,623.59, and 1,826.67, respectively, were selected as precursor ions. For B) and D) the doubly-charged $[M\text{-}2H]^{2-}$ molecular ions at *m/z* 892.32, and 1,188.93, respectively, were selected as precursor ions. In panel D), the inset is a zoomed-region of the mass spectrum in the *m/z* 100-1,000 range.

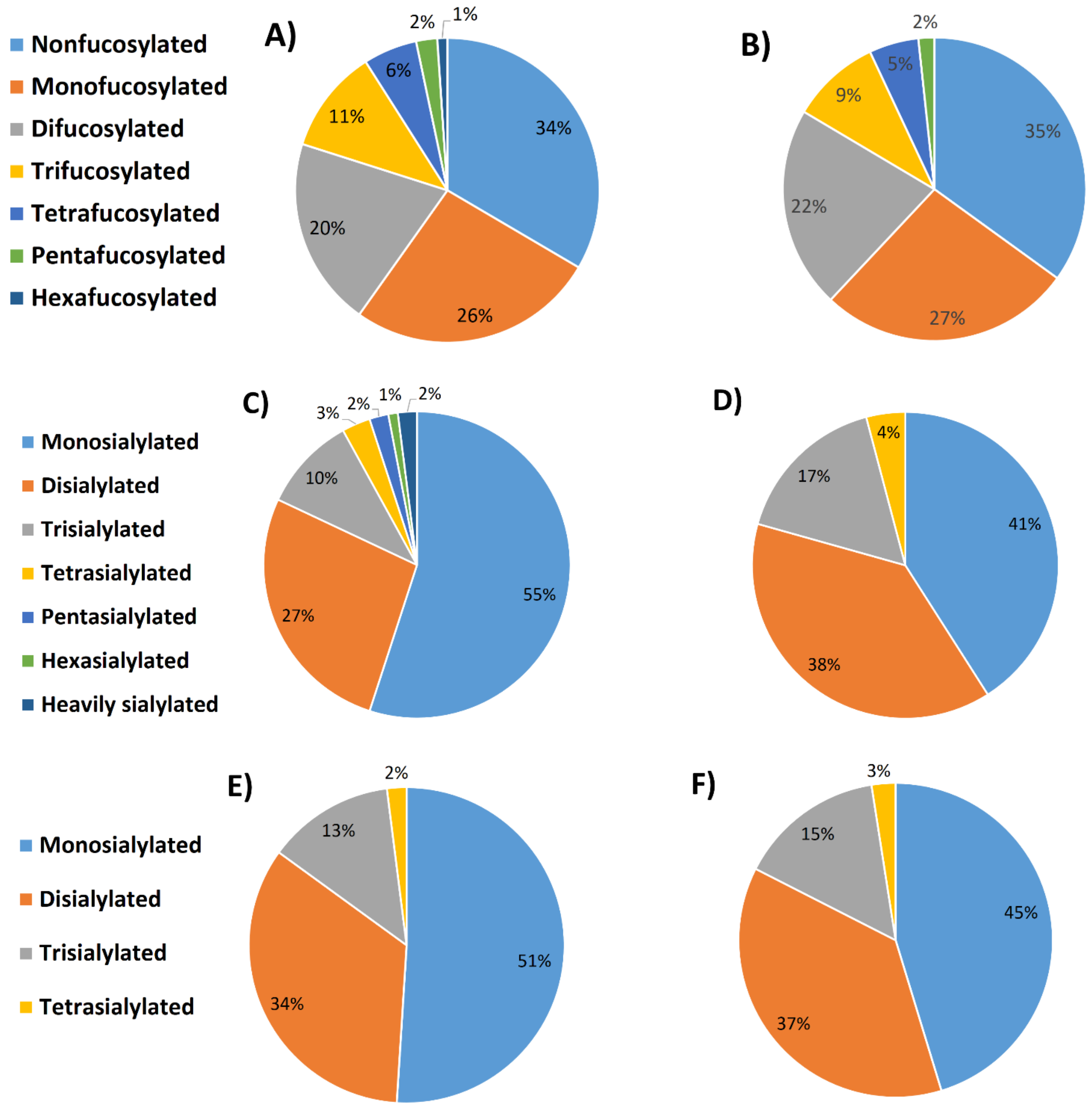

Supplementary Figure 10: CZE-MS-based **N-glycan profiling of bovine serum fetuin** using injected amounts equivalent to **~5 ng** (A, C, E) and **~0.5 ng** (B, D, F) of proteins (in total, 401 and 232 N-glycans were identified using 5 ng and 0.5 ng of fetuin, respectively). A-B) Fractions of fucosylated glycans detected in the BSF isolate. C-D) Fractions of sialylated glycans containing only Neu5Ac monosaccharide. E-F) Fractions of sialylated glycans containing only Neu5Gc monosaccharide. An injected amount of ~5 ng of proteins results in the detection of extremely low abundance highly fucosylated (6 Fuc residues) and highly sialylated (≥5 SiA residues) glycans in the BSF isolate. The detected heavily sialylated glycans contain between 7 and 10 SiA residues.

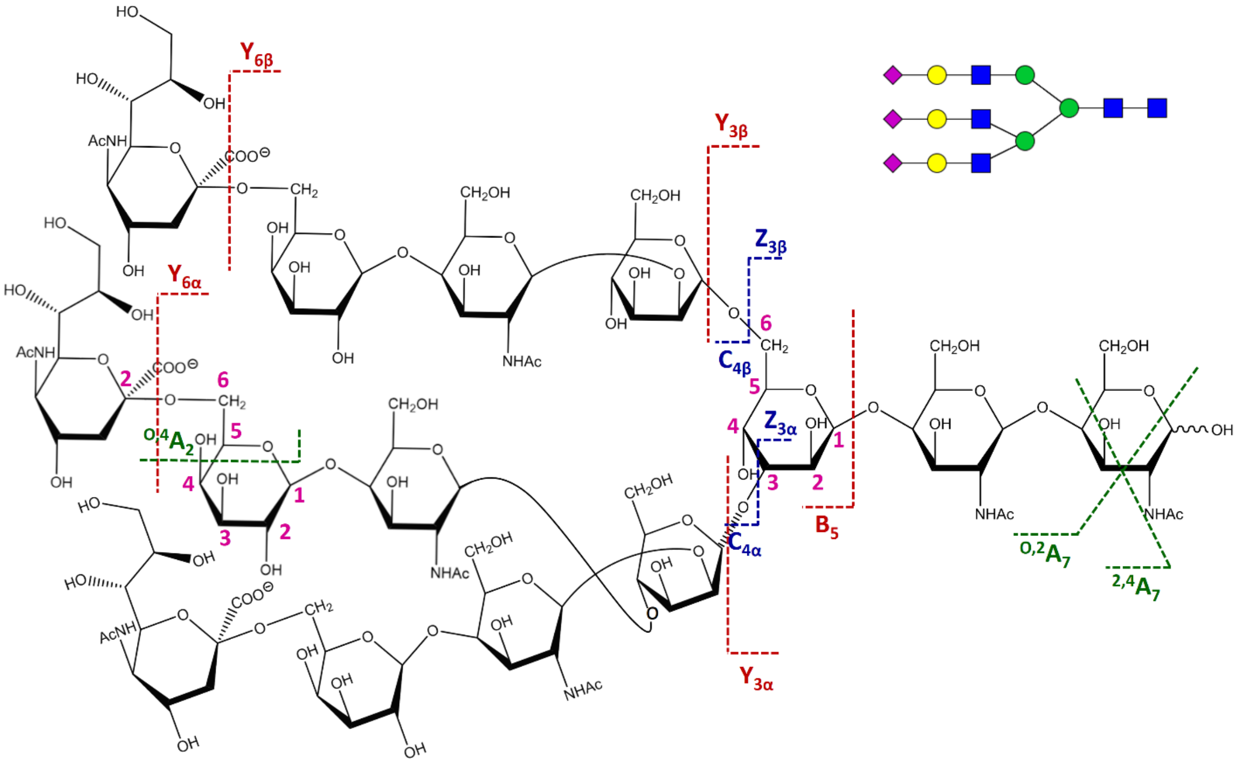

Supplementary Figure 11: ***In-silico*** **predicted fragmentation cleavages** in negative ion mode of the tri-antennary glycan **A3G3S3** with a branched 3-linked antenna. The diagnostic ions $B_5/Z_{3\alpha}$, $B_5/Y_{3\alpha}$, $C_{4\alpha}$ and $C_{4\beta}$ enable the assignment of the 3-branched antenna (i.e., antenna attached to the carbon 3 of the core mannose).

Supplementary Note 5

For the MS^2 structural characterization of **FA3G3S3**, the triply-charged ion at *m/z* 1,007.35 was selected as a precursor (**Supplementary Figure 12**). The diagnostic ions $B_5/Z_{3\alpha}{}^{1-}$ (*m/z* 961.31) and $B_5/Y_{3\alpha}{}^{1-}$ (*m/z* 979.32) allowed us to allocate the branched 3-linked antenna. Detection of the fragment $Y_6/Y_6{}^{1-}$ at *m/z* 2,441.86 indicated the presence of one additional fucosyl residue. The mass difference of 206.08 Da between ${}^{2,4}A_7{}^{3-}$ (*m/z* 904.97) and ${}^{0,2}A_7{}^{3-}$ (*m/z* 973.66), and ${}^{2,4}A_7/Y_6{}^{2-}$ (*m/z* 1,212.41) and ${}^{0,2}A_7/Y_6{}^{2-}$ (*m/z* 1,315.45) ions confirmed the location of the fucose on the chitobiose core [8]. Other fragments were mostly similar to those obtained from the fragmentation of the non-fucosylated analog. The α-2,6 Neu5Ac diagnostic ion ${}^{0,4}A_2\text{-}CO_2{}^{1-}$ was also observed in the mass spectra of the fucosylated glycan. The analyses performed without applying SP over the whole duration of the CZE run made possible to separate (at resolution 0.6) this highly abundant fucosylated glycan from a positional isomer **A3F1G3S3** with an outer arm fucose (**Supplementary Figure 13)**. For A3F1G3S3, two pairs of fragment ions with a mass difference of 60.02 Da, ${}^{2,4}A_7{}^{3-}$ (*m/z* 953.66) and ${}^{0,2}A_7{}^{3-}$ (*m/z* 973.67), and ${}^{2,4}A_7/Y_6{}^{2-}$ (*m/z* 1,285.45) and ${}^{0,2}A_7/Y_6{}^{2-}$ (*m/z* 1,315.46), confirmed the absence of a core fucose, and fragments like $C_{4\alpha}/Y_{5\alpha}{}^{2-}$ (*m/z* 745.25) and $C_{4\beta}{}^{1-}$ (*m/z* 835.28) located the fucose on the 3-branched antenna.

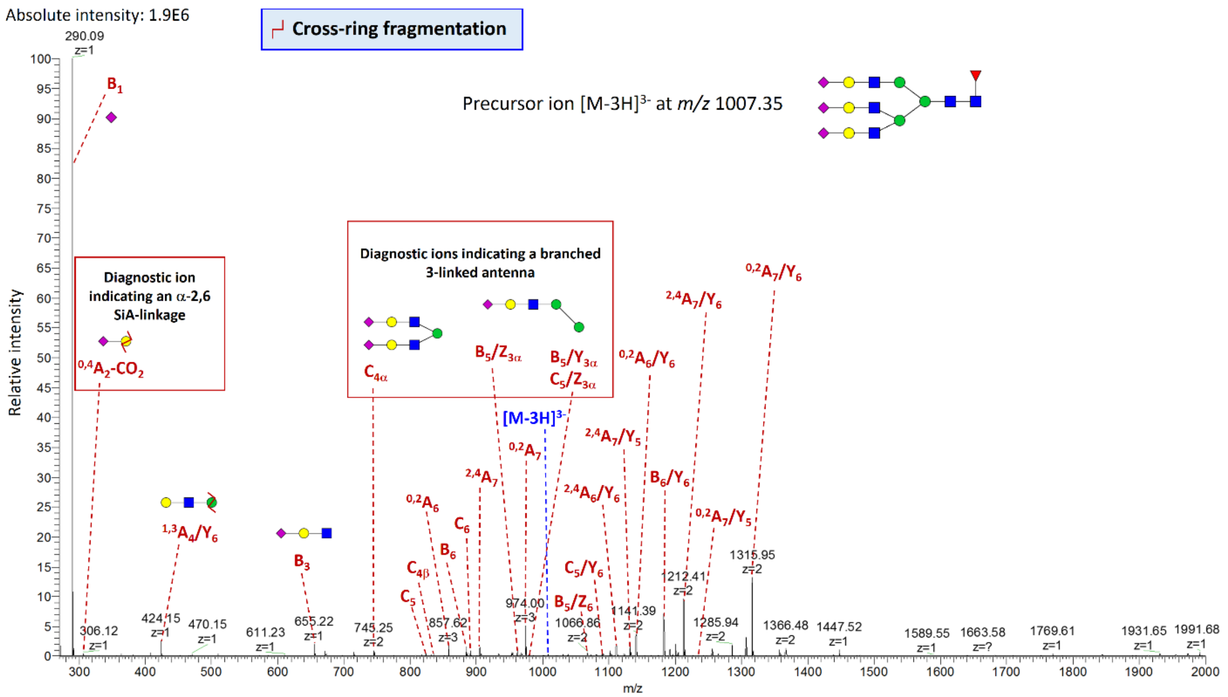

Supplementary Figure 12: An example of MS^2 spectrum of the fucosylated glycan **FA3G3S3**. The $[M\text{-}3H]^{3-}$ molecular ion at *m/z* 1,007.35 was selected as a precursor ion. The mass difference of 206.08 Da between the pair of ions $^{2,4}A_7^{3-}$ and $^{0,2}A_7^{3-}$, and $^{2,4}A_7/Y_6^{2-}$ and $^{0,2}A_7/Y_6^{2-}$ ions confirms the presence of the fucose on the chitobiose core.

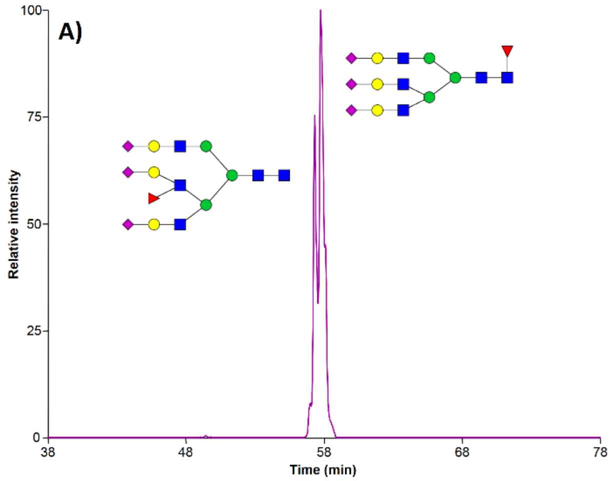

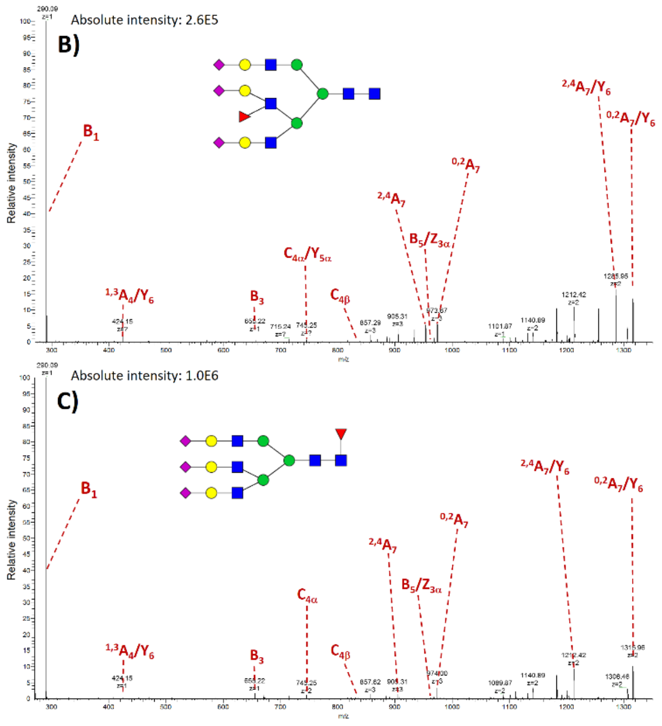

Supplementary Figure 13: An example of **separation of fucose isomers** is demonstrated for label-free CZE-MS analysis of N-glycans released from bovine serum fetuin. A) EIEs of the fucose isomers. B) and C) MS^2 spectra of the outer arm fucosylated isomer (B) and the core fucosylated isomer (C). The shift of the $^{2,4}A_7^{3-}$ and $^{2,4}A_7/Y_6^{2-}$ ions in the mass spectrum of FA3G3S3 confirms the location of the fucose on the chitobiose core.

Supplementary Note 6

The second most abundant N-glycan detected in bovine fetuin was the tetrasialylated tri-antennary glycan **A3G3S4** (Mr_{th} 3,170.11 Da). The structural characterization of this glycan was based on the triply-charged precursor ion at *m/z* 1,055.69 (**Supplementary Figure 14AB**). B_1^{1-} (*m/z* 290.09) ions were detected at high abundance. The diagnostic ions $B_5/Z_{3\alpha}^{1-}$ (*m/z* 961.31) and $B_5/Y_{3\alpha}^{1-}$ (*m/z* 979.32) allowed us to assign a branched 3-linked antenna. The fragment ion at *m/z* 745.25, corresponding in this case to $C_{4\alpha}/Y_{5\alpha}^{2-}$, was detected with a higher abundance (~3-fold) than the $C_{4\beta}^{1-}$ ion at *m/z* 835.28, and confirmed a branched 3-linked antenna with three sialylated residues. A mass difference of 60.02 Da between the two pairs of ions $^{2,4}A_7/Y_6^{3-}$ (*m/z* 904.97) and $^{0,2}A_7/Y_6^{3-}$ (*m/z* 924.98), and $^{2,4}A_7/Y_6/Y_6^{2-}$ (*m/z* 1,212.41) and $^{0,2}A_7/Y_6/Y_6^{2-}$ (*m/z* 1,242.42) was indicative of the absence of a core fucose. The presence of α-2,6 SiA linkages was confirmed by the detection of $^{0,4}A_2$-CO_2^{1-} (*m/z* 306.12), $^{3,5}X_5/Z_6^{2-}$ (*m/z* 1,246.93) and $^{1,3}X_5/Z_6^{2-}$ (*m/z* 1,253.93) ions. The fucosylated analog **FA3G3S4** was also detected in the fetuin isolate at low abundance levels. The shift of the fragments $^{0,2}A_7/Y_6^{3-}$ and $^{0,2}A_7/Y_6/Y_6^{2-}$ at *m/z* 973.66 and 1,315.45, respectively, allowed the unambiguous assignment of the core fucose (**Supplementary Figure 14C**). Other tetrasialylated glycans were detected by MS^2, e.g., the tetra-antennary oligosaccharide **A4G4S4** (Mr_{th} 3,535.24 Da) (**Supplementary Figure 15**). Selecting the triply-charged precursor ion at *m/z* 1,177.74, the structure of A4G4S4 could be fully characterized. B_1^{1-} (*m/z* 290.09) ions were detected at high abundance, and the loss of one GlcNAc residue from the non-labeled reducing

end was detected with the glycosidic fragment Z_1^{1-} (*m/z* 202.07). The fragmentation spectra of A4G4S4 exhibited a high number of glycosidic and glycosidic/glycosidic fragments like Y_6^{3-} (*m/z* 1,080.37), Z_6^{3-} (*m/z* 1,074.37), Y_4/Y_4^{2-} (*m/z* 1,110.39), and Y_6/Y_6^{2-} (*m/z* 1,475.51) ions. In addition, α-2,6 SiA linkages were confirmed by the presence of $^{0,4}A_2\text{-}CO_2^{1-}$ ion.

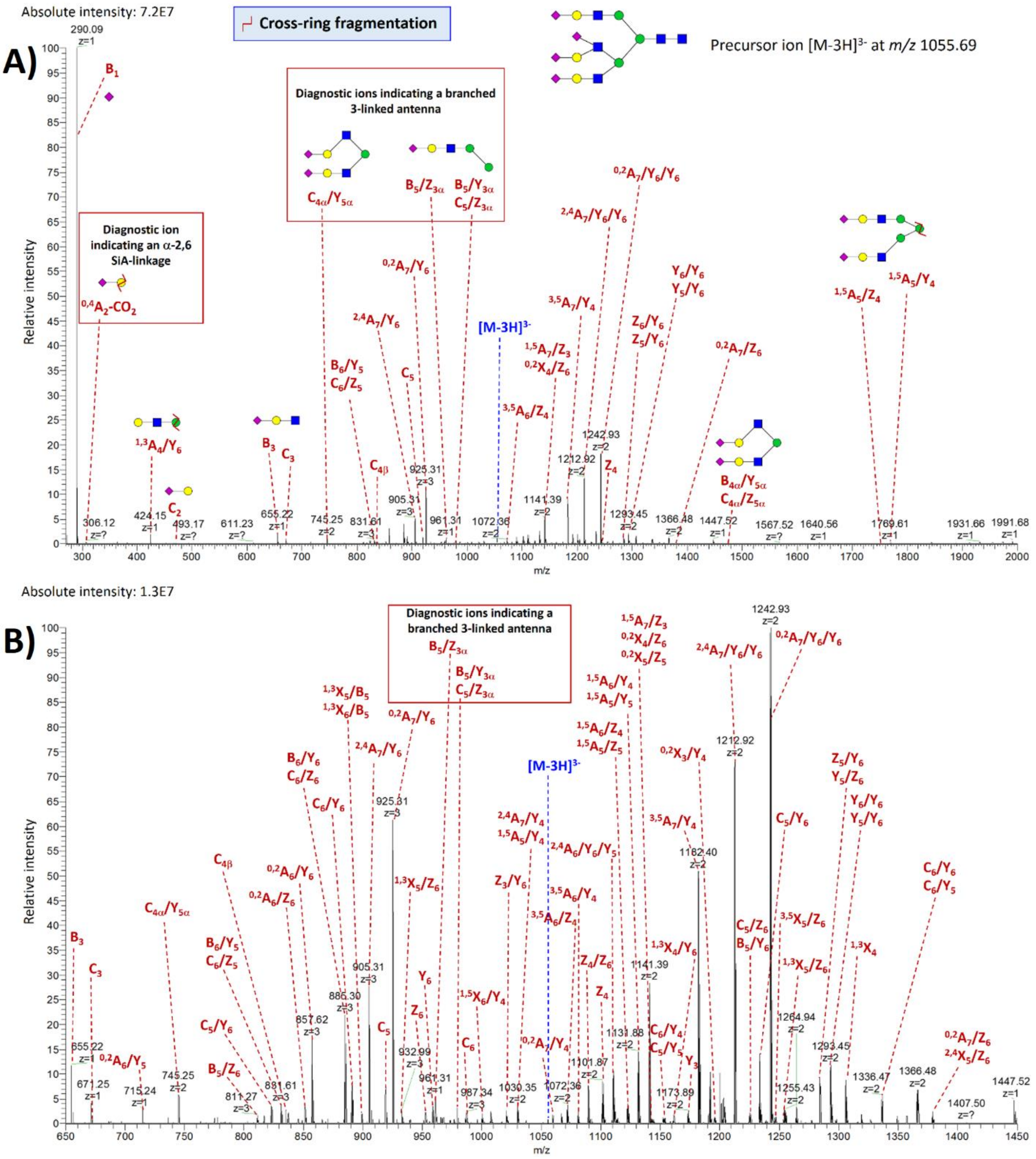

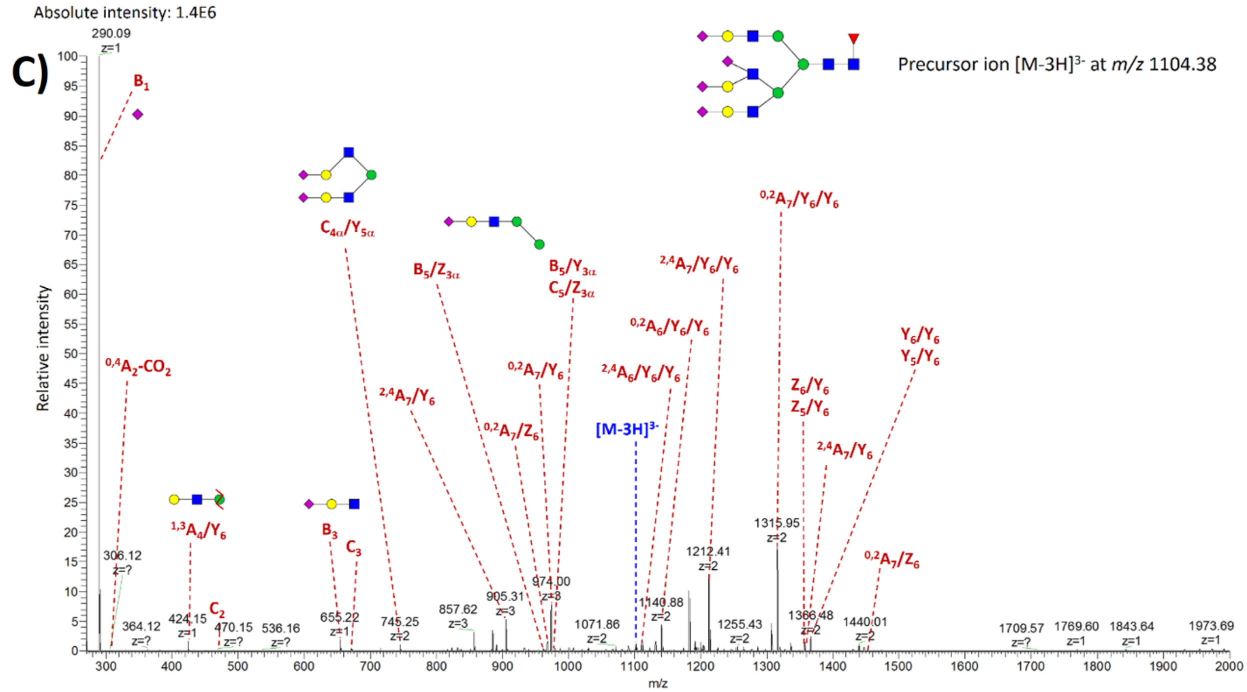

Supplementary Figure 14: Examples of characteristic MS^2 spectra of **A3G3S4** (A-B) and its fucosylated analog **FA3G3S4** (C) acquired in negative ion mode. The panels A) and B) show the same mass spectrum at different signal intensity and *m/z* ranges. The $[M-3H]^{3-}$ molecular ions at *m/z* 1,055.69, and 1,104.38, respectively, were selected as precursor ions. In panel C) the shift of the fragments $^{0,2}A_7/Y_6^{3-}$ and $^{0,2}A_7/Y_6/Y_6^{2-}$ enables the unambiguous assignment of the core fucose.

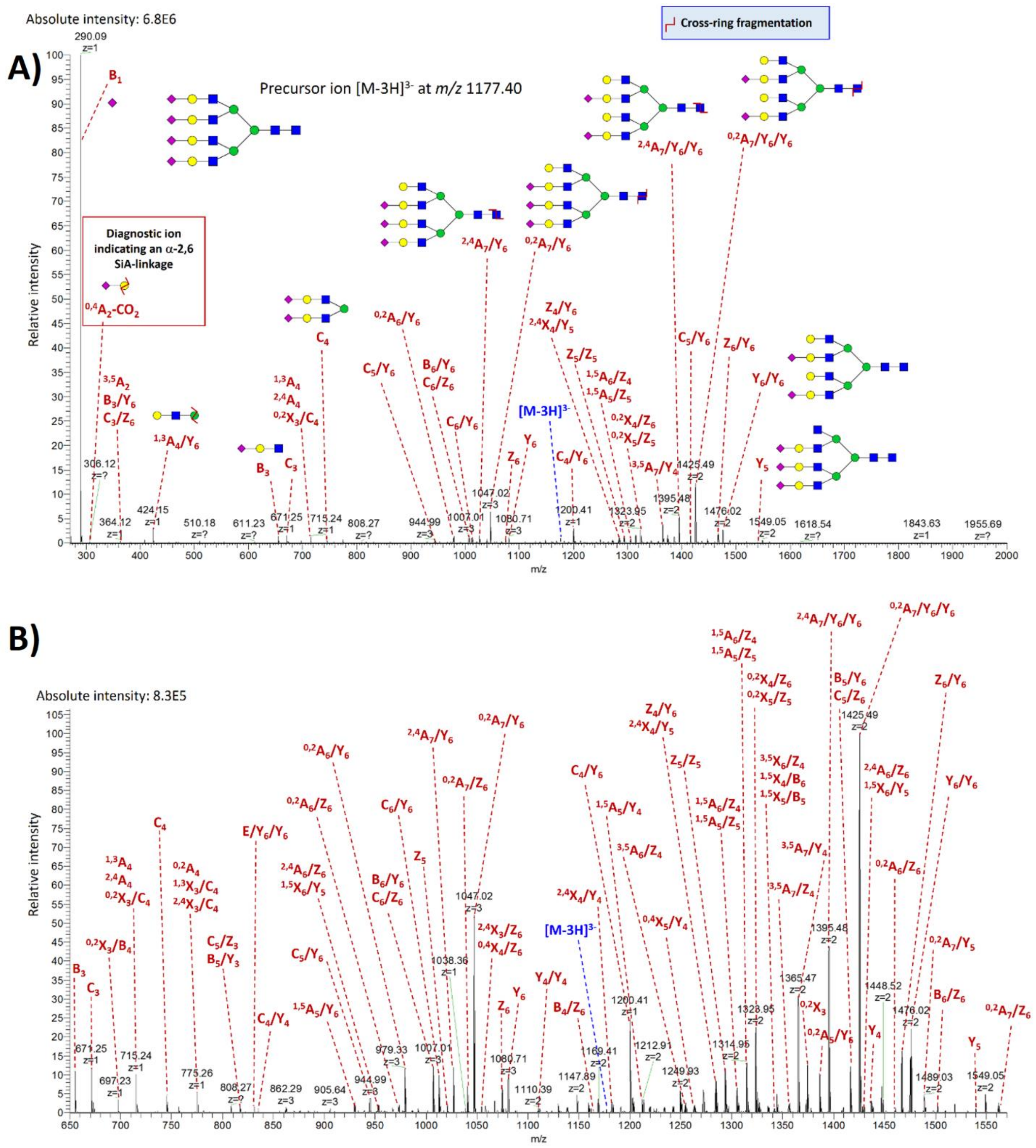

Supplementary Figure 15: An example of MS^2 spectrum of the tetra-antennary tetrasialylated N-glycan **A4G4S4**. The panels A) and B) show the same mass spectrum at different signal intensity and *m/z* ranges. The $[M-3H]^{3-}$ molecular ion at *m/z* 1,177.40 was selected as a precursor ion.

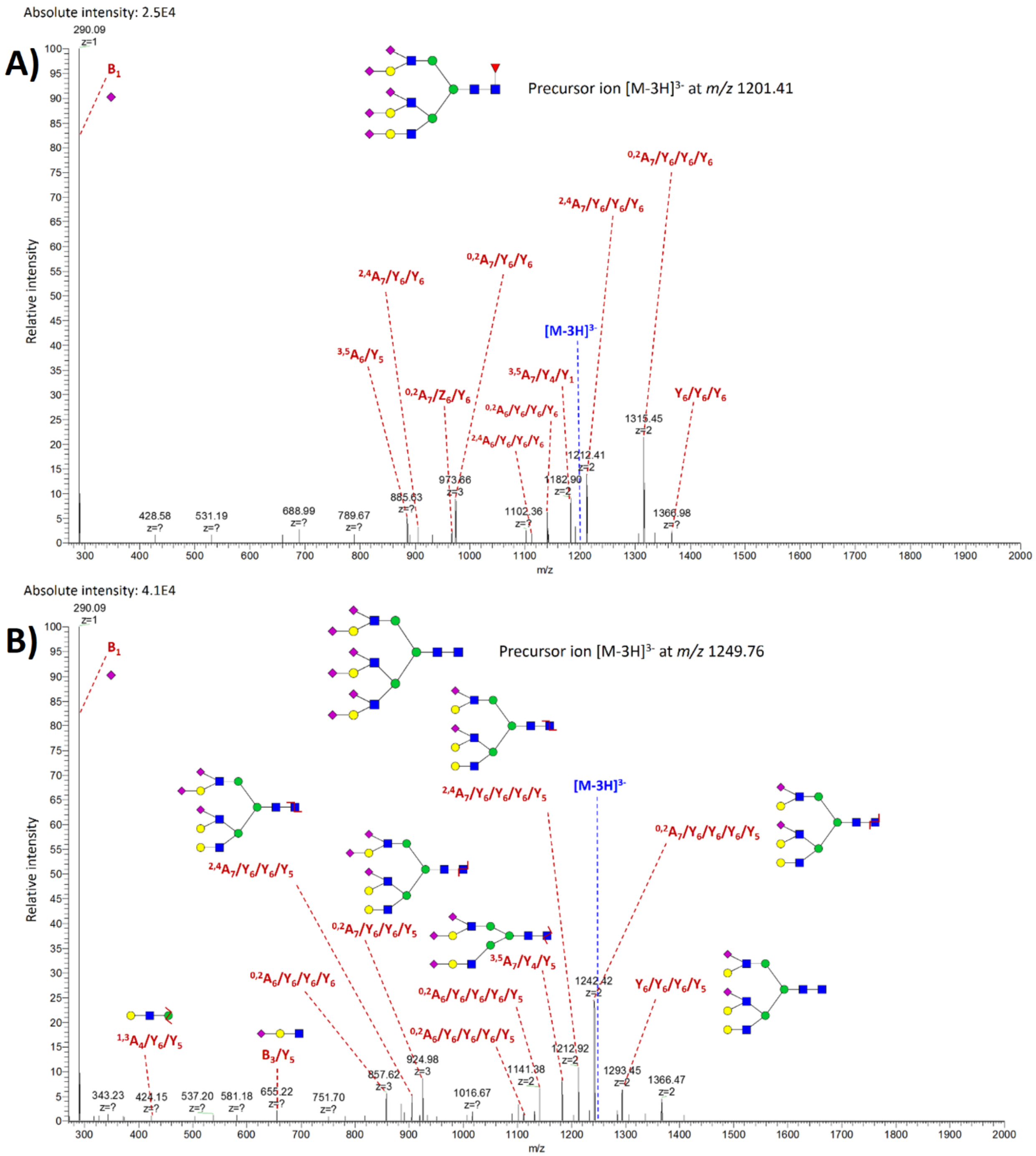

Supplementary Figure 16: Examples of characteristic MS^2 spectra in negative ion mode of the fucosylated pentasialylated N-glycan **FA3G3S5** and the hexasialylated N-glycan **A3G3S6**. For MS^2-based structural characterization the $[M-3H]^{3-}$ molecular ions at *m/z* 1,201.41, and 1,249.76, respectively, were selected as precursor ions.

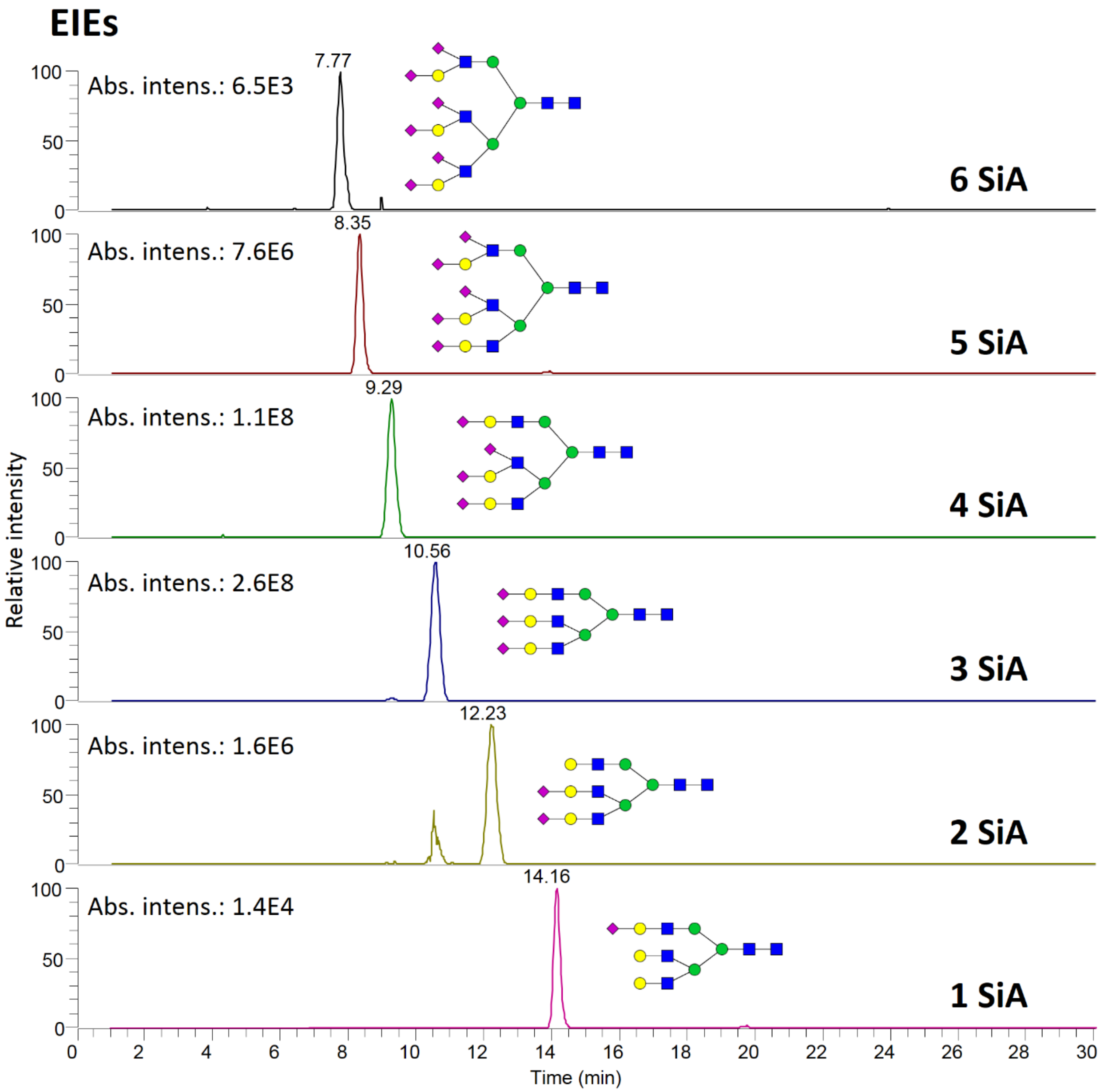

Supplementary Figure 17: Impact of the level of sialylation on the electrophoretic migration of sialylated N-glycans. **EIEs of six representative sialylated N-glycans** acquired in the label-free CZE-MS analysis of fetuin-derived N-glycans. A strong correlation is shown between the migration time shift and each incrementation of one additional SiA residue.

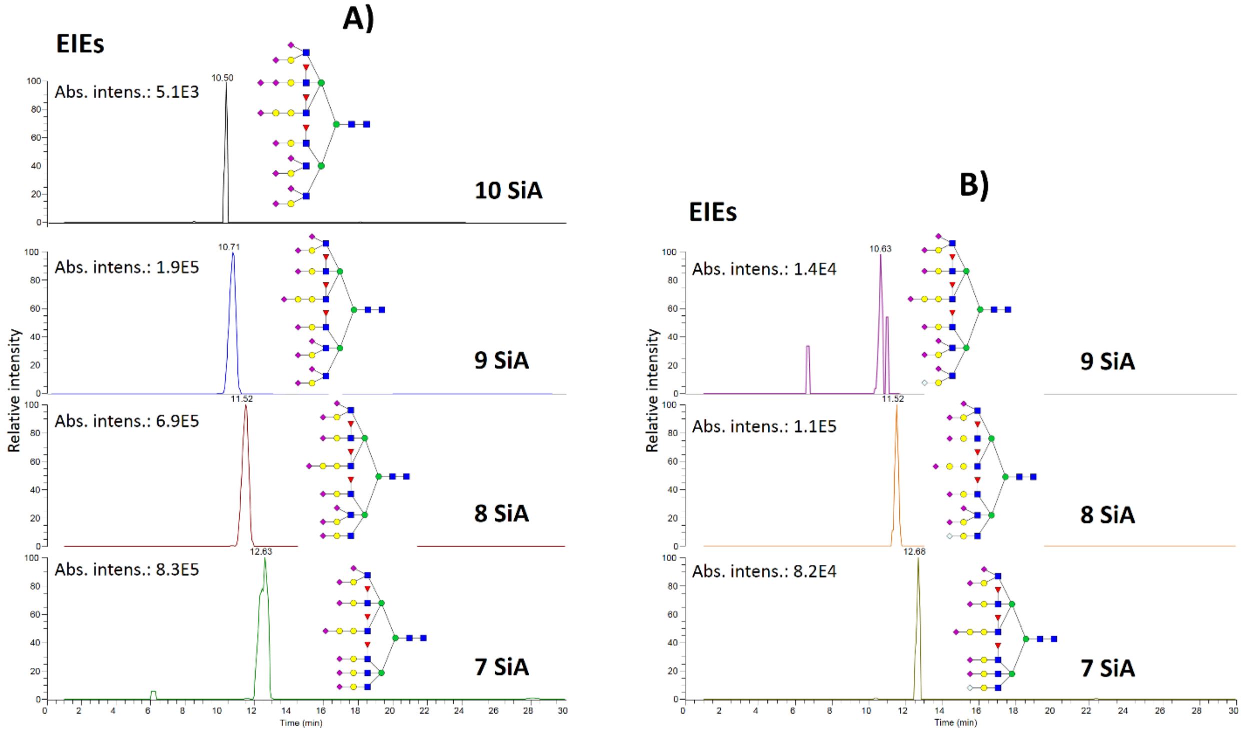

Supplementary Figure 18: Label-free CZE-MS analyses of **heavily sialylated N-glycans** released from the bovine serum fetuin isolate. EIEs of four heavily sialylated N-glycans containing only Neu5Ac residues (A) and EIEs of three heavily sialylated N-glycans containing Neu5Ac residues and one Neu5Gc residue (B). The migration of these sialylated glycans is governed by the level of sialylation. Purple diamond, Neu5Ac; light blue diamond, Neu5Gc.

Supplementary Note 7

Supplementary Figure 19CD shows a characteristic MS^2 spectrum of the glycan carrying nine Neu5Ac residues, detected at high abundance in the fetuin isolate. The structural characterization of this nonasialylated glycan was based on the quintuply-charged precursor ion at *m/z* 1,263.23. The highest intensity fragment ion was the fragment B_1^{1-} (*m/z* 290.09). The fragment ions $Y_6/Y_6/Y_6^{5-}$ (*m/z* 1,088.58), $Y_6/Y_6/Y_6/Y_6^{4-}$ (*m/z* 1,288.20), and $Y_6/Y_6/Y_6/Y_6/Y_6^{3-}$ (*m/z* 1,620.90) derived from multiple internal fragmentation cleavages were also detected at high abundance, and corresponded to the loss of 3, 4, and 5 Neu5Ac residues, respectively. A mass difference of 60.02 Da between the two pairs of ions $^{2,4}A_8/Y_6/Y_5/Y_6/Y_4/Y_4/Y_4^{3-}$ (*m/z* 904.97) and $^{0,2}A_8/Y_6/Y_5/Y_6/Y_4/Y_4/Y_4^{3-}$ (*m/z* 924.98), and $^{2,4}A_8/Y_6/Y_5/Y_6/Y_4/Y_4/Y_4/Y_6^{2-}$ (*m/z* 1,212.41) and $^{0,2}A_8/Y_6/Y_5/Y_6/Y_4/Y_4/Y_4/Y_6^{2-}$ (*m/z* 1,242.42) was indicative of the absence of a core fucose.

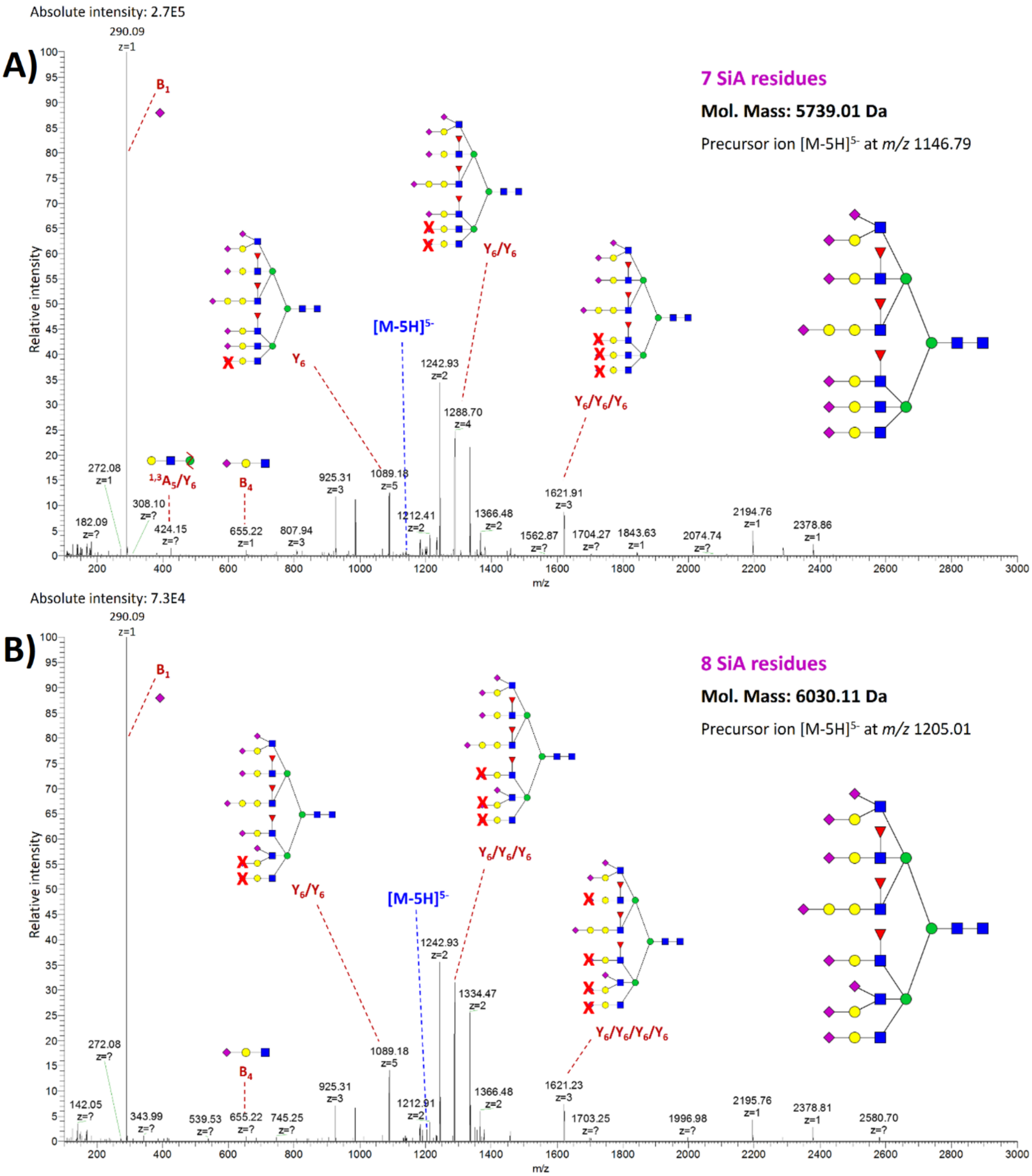
Absolute intensity: 2.7E5
A)
7 SiA residues
Mol. Mass: 5739.01 Da
Precursor ion [M-5H]5- at m/z 1146.79
B1
Y6
Y6/Y6
Y6/Y6/Y6
[M-5H]5-
1,3A5/Y6
B4
Relative intensity
m/z
Absolute intensity: 7.3E4
B)
8 SiA residues
Mol. Mass: 6030.11 Da
Precursor ion [M-5H]5- at m/z 1205.01
B1
Y6/Y6
Y6/Y6/Y6
Y6/Y6/Y6/Y6
[M-5H]5-
B4
Relative intensity
m/z

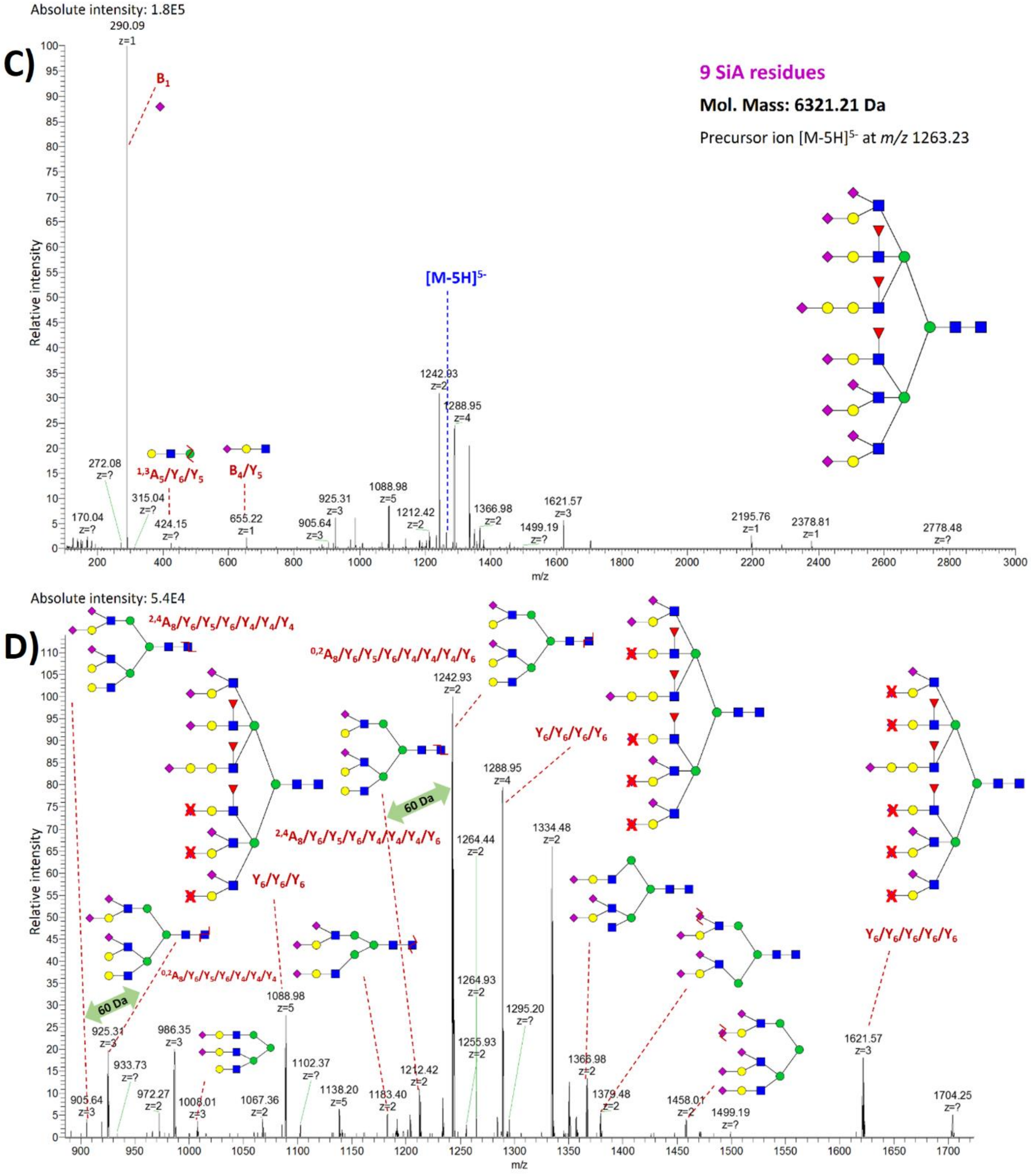

Supplementary Figure 19: Examples of characteristic MS^2 spectra in negative ion mode of **heavily sialylated N-glycans containing solely Neu5Ac residues**. Putative structures were proposed based on the fragmentation patterns of these large size glycans. The panels C) and D) show the same mass spectrum at different signal intensity and *m/z* ranges. For MS^2-based structural characterization the quintuply-charged molecular ions at *m/z* 1,146.79 (A), 1,205.01 (B), and 1,263.23 (C-D), respectively, were selected as precursor ions. Panel D) shows the typical mass difference of 60.02 Da between the two pairs of ions $^{2,4}A_8/Y_6/Y_5/Y_6/Y_4/Y_4/Y_4^{3-}$ and

$^{0,2}A_8/Y_6/Y_5/Y_6/Y_4/Y_4/Y_4^{3-}$, and $^{2,4}A_8/Y_6/Y_5/Y_6/Y_4/Y_4/Y_4/Y_6^{2-}$ and $^{0,2}A_8/Y_6/Y_5/Y_6/Y_4/Y_4/Y_4/Y_6^{2-}$, indicative of the absence of a core fucose. Purple diamond, Neu5Ac; light blue diamond, Neu5Gc. The red cross symbol relates to the loss of the selected monosaccharide.

Supplementary Note 8

The disialylated glycan **A2G2S2** was detected as a set of three different glycans terminated with either two Neu5Ac, or two Neu5Gc, or both Neu5Ac and Neu5Gc. The glycan terminated with two Neu5Ac residues (Mr_{th} 2,222.78 Da), the most abundant one among the three types of detected A2G2S2, was structurally characterized by selecting the precursor ion at *m/z* 1,110.38 **(Supplementary Figure 20A)**. The fragmentation pattern of this glycan containing only Neu5Ac was similar to that described in the previous section. Therefore, the series of fragment ions B_1^{1-} (*m/z* 290.09), B_3^{1-} (*m/z* 655.22), C_4^{1-} (*m/z* 835.28), B_6^{2-} (*m/z* 999.84), C_6^{2-} (*m/z* 1,008.84), Z_6^{1-} (*m/z* 1,912.66), Y_6^{1-} (*m/z* 1,930.67), $^{2,4}A_7^{2-}$ (*m/z* 1,029.85), $^{0,2}A_7^{2-}$ (*m/z* 1,059.86), $^{2,4}A_7/Y_6^{1-}$ (*m/z* 1,769.61), and $^{0,2}A_7/Y_6^{1-}$ (*m/z* 1,829.63) were detected. Additionally, the detection of $^{0,4}A_2\text{-}CO_2^{1-}$ ion at *m/z* 306.12 reflected the presence of α-2,6-linked Neu5Ac. The A2G2S2 glycan terminated with one Neu5Ac and one Neu5Gc residues (Mr_{th} 2,238.78 Da), less abundant, was structurally characterized by selecting the $[M\text{-}2H]^{2-}$ molecular ion at *m/z* 1,118.38 **(Supplementary Figure 20B)**. Two highly abundant B_1^{1-} fragment ions were detected at *m/z* 290.09 and 306.08 (this latter ion being unequivocally distinguished from the diagnostic ion at *m/z* 306.12 using high resolution and high mass accuracy measurements), and corresponded to the loss of Neu5Ac and Neu5Gc, respectively. The fragment ions that allowed us to allocate one Neu5Ac residue on one antenna (e.g., Z_6 and Y_6) were similar to those described above. The presence of Neu5Gc on the other antenna was confirmed by the shift of B_3^{1-}, C_4^{1-}, B_6^{2-}, C_6^{2-}, Z_6^{1-}, and Y_6^{1-} fragment ions detected at *m/z* 671.21, 851.28, 1,007.84, 1,016.84, 1,928.66, and 1,946.67, respectively. A similar shift was observed with the cross-ring fragments $^{2,4}A_7^{2-}$, $^{0,2}A_7^{2-}$, $^{2,4}A_7/Y_6^{1-}$ and $^{0,2}A_7/Y_6^{1-}$, detected at *m/z* 1,037.84, 1,067.85, 1,785.60, and 1,845.62, respectively. A singly-charged diagnostic ion $^{0,4}A_2\text{-}CO_2$ was detected at *m/z* 322.12, revealing that the Neu5Gc sialic acid was α-2,6-linked [9]. The α-2,6 Neu5Ac diagnostic ion at *m/z* 306.12 was not detected, but this ion could overlap with the highly abundant B_1^{1-} ion at *m/z* 306.08. The A2G2S2 glycan terminated with two Neu5Gc residues (Mr_{th} 2,254.77 Da), detected at very low abundance, was characterized based on the fragmentation of the $[M\text{-}2H]^{2-}$ molecular ion at *m/z* 1,126.38 **(Supplementary Figure 20C)**. The B_1^{1-} fragment detected at *m/z* 306.08 corresponded to the loss of the Neu5Gc residues. A shift of the glycosidic fragments B_6^{2-} and C_6^{2-}, and the cross-ring fragments $^{2,4}A_7^{2-}$ and $^{0,2}A_7^{2-}$, detected at *m/z* 1,015.83, 1,024.84, 1,045.84, and 1,075.85, respectively, could be observed, in accordance with one additional Neu5Gc. The fragment ion $^{0,4}A_2\text{-}CO_2^{1-}$ at *m/z* 322.12, diagnostic of an α-2,6 Neu5Gc linkage, was not detected in this case, possibly due to its very low abundance.

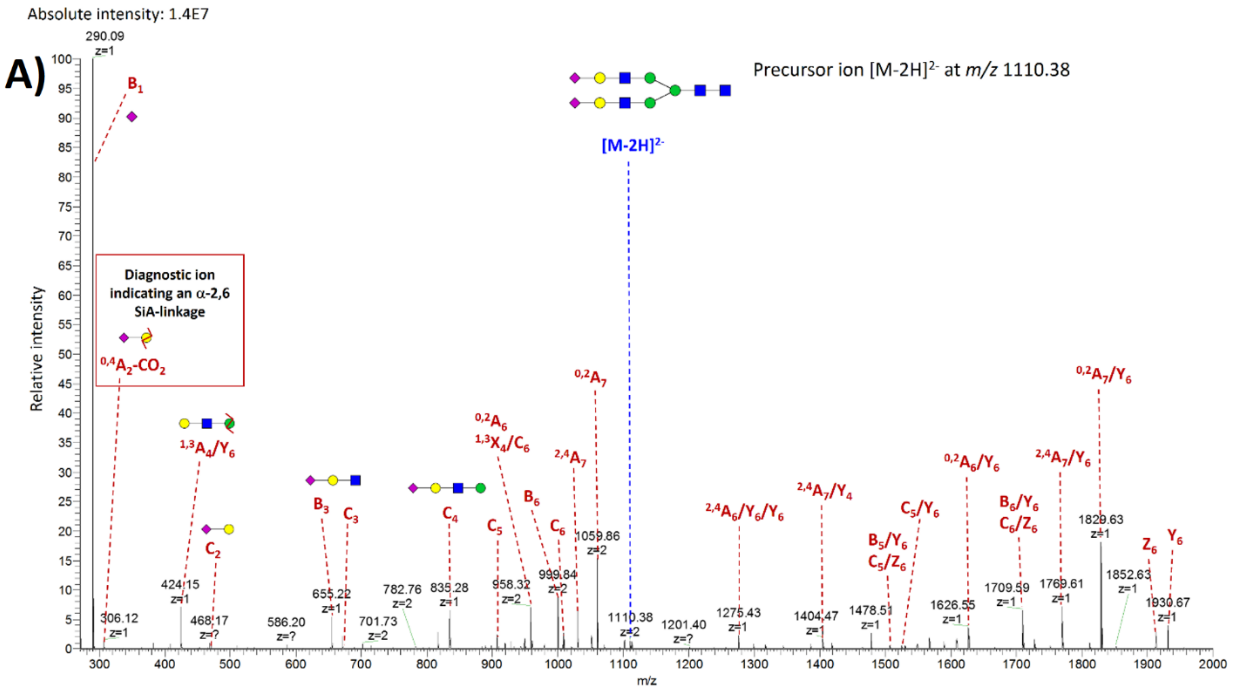
Absolute intensity: 1.4E7
A)
Precursor ion [M-2H]2- at m/z 1110.38
[M-2H]2-
Diagnostic ion indicating an α-2,6 SiA-linkage
0,4A2-CO2
B1
1,3A4/Y6
C2
B3
C3
C4
C5
0,2A6
1,3X4/C6
B6
C6
2,4A7
0,2A7
2,4A6/Y6/Y6
2,4A7/Y4
B5/Y6
C5/Z6
C5/Y6
0,2A6/Y6
B6/Y6
C6/Z6
2,4A7/Y6
0,2A7/Y6
Z6
Y6
Relative intensity
m/z
290.09 z=1
306.12 z=1
424.15 z=1
468.17 z=?
586.20 z=?
655.22 z=1
701.73 z=2
782.76 z=2
835.28 z=1
958.32 z=2
999.84 z=2
1059.86 z=2
1110.38 z=2
1201.40 z=?
1275.43 z=1
1404.47 z=1
1478.51 z=1
1626.55 z=1
1709.59 z=1
1769.61 z=1
1829.63 z=1
1852.63 z=1
1930.67 z=1

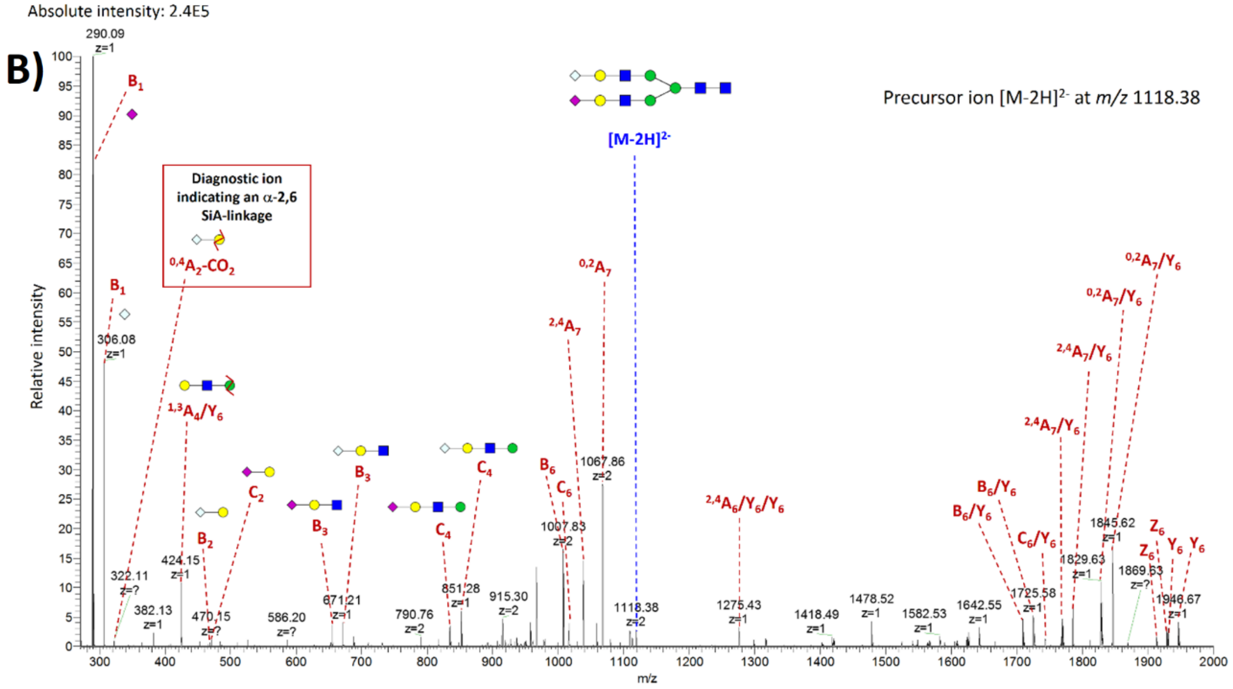
Absolute intensity: 2.4E5
B)
Precursor ion [M-2H]2- at m/z 1118.38
[M-2H]2-
Diagnostic ion indicating an α-2,6 SiA-linkage
0,4A2-CO2
B1
B1
1,3A4/Y6
B2
C2
B3
B3
C4
C4
0,2A7
2,4A7
B6
C6
2,4A6/Y6/Y6
B6/Y6
B6/Y6
C6/Y6
2,4A7/Y6
2,4A7/Y6
0,2A7/Y6
0,2A7/Y6
Z6
Z6
Y6
Y6
Relative intensity
m/z
290.09 z=1
306.08 z=1
322.11 z=?
382.13 z=1
424.15 z=1
470.15 z=?
586.20 z=?
671.21 z=1
790.76 z=2
851.28 z=1
915.30 z=2
1007.83 z=2
1067.86 z=2
1118.38 z=2
1275.43 z=1
1418.49 z=1
1478.52 z=1
1582.53 z=1
1642.55 z=1
1725.58 z=1
1829.63 z=1
1845.62 z=1
1869.63 z=?
1946.67 z=1

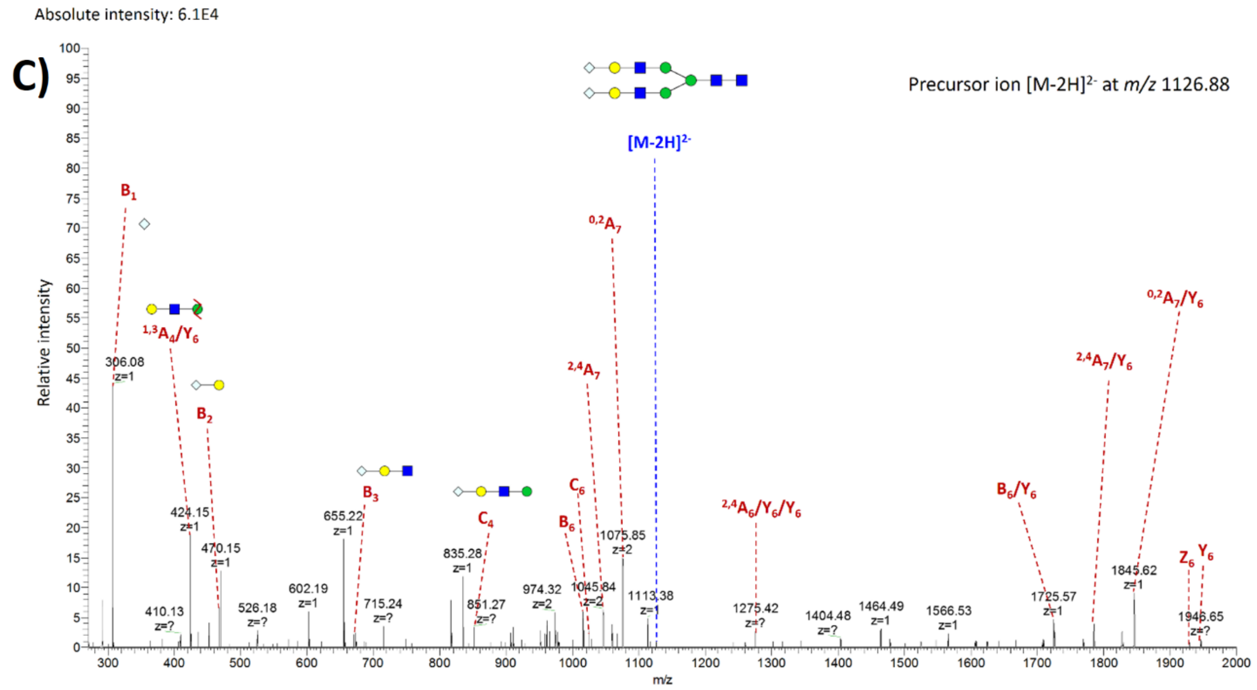

Supplementary Figure 20: Examples of characteristic MS^2 spectra of the disialylated N-glycan **A2G2S2** containing two Neu5Ac residues (A), one Neu5Ac and one Neu5Gc residues (B), and two Neu5Gc residues (C). The $[M-2H]^{2-}$ molecular ions at 1,110.38, 1,118.38, and 1,126.88, respectively, were selected as precursor ions. Purple diamond, Neu5Ac; light blue diamond, Neu5Gc.

A)

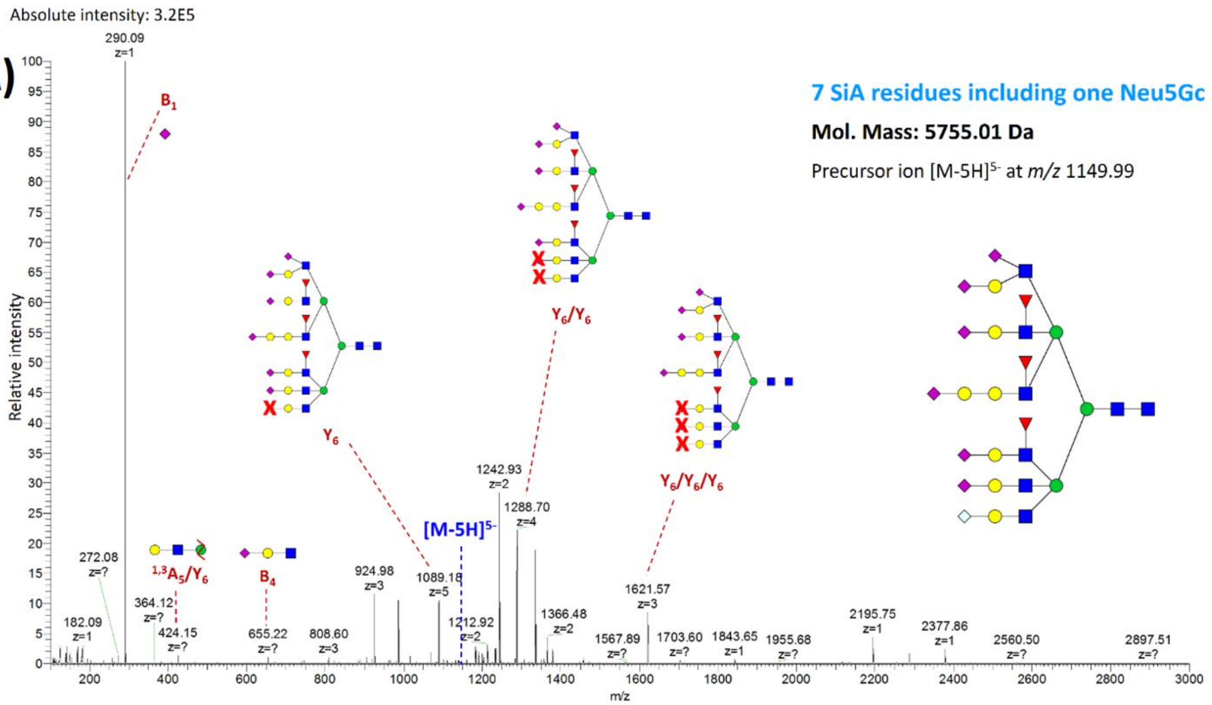
Absolute intensity: 3.2E5
7 SiA residues including one Neu5Gc
Mol. Mass: 5755.01 Da
Precursor ion [M-5H]5- at m/z 1149.99
Relative intensity
m/z
290.09 z=1
B1
272.08 z=?
182.09 z=1
364.12 z=?
424.15 z=?
1,3A5/Y6
B4
655.22 z=?
808.60 z=3
924.98 z=3
1089.18 z=5
Y6
[M-5H]5-
1212.92 z=2
1242.93 z=2
1288.70 z=4
Y6/Y6
1366.48 z=2
1567.89 z=?
1621.57 z=3
Y6/Y6/Y6
1703.60 z=?
1843.65 z=1
1955.68 z=?
2195.75 z=1
2377.86 z=1
2560.50 z=?
2897.51 z=?

B)

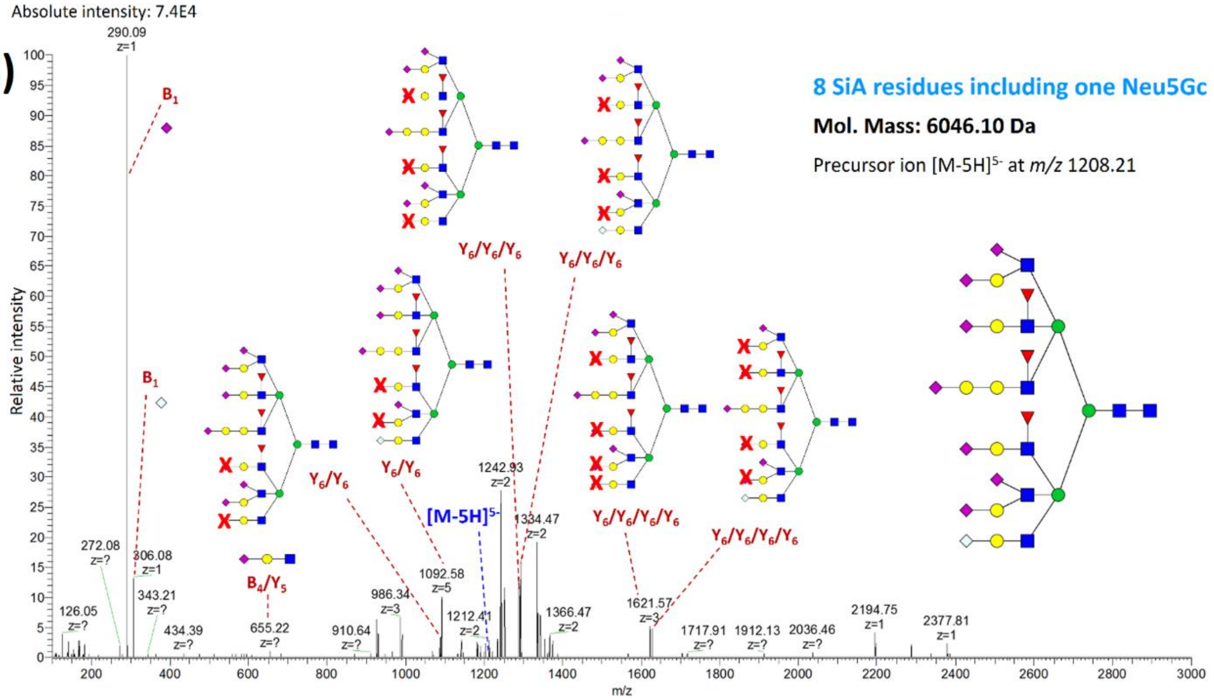
Absolute intensity: 7.4E4
8 SiA residues including one Neu5Gc
Mol. Mass: 6046.10 Da
Precursor ion [M-5H]5- at m/z 1208.21
Relative intensity
m/z
290.09 z=1
B1
B1
126.05 z=?
272.08 z=?
306.08 z=1
343.21 z=?
434.39 z=?
B4/Y5
655.22 z=?
910.64 z=?
986.34 z=3
1092.58 z=5
Y6/Y6
Y6/Y6
[M-5H]5-
1212.41 z=2
1242.93 z=2
1334.47 z=2
Y6/Y6/Y6
Y6/Y6/Y6
1366.47 z=2
Y6/Y6/Y6/Y6
Y6/Y6/Y6/Y6
1621.57 z=3
1717.91 z=?
1912.13 z=?
2036.46 z=?
2194.75 z=1
2377.81 z=1

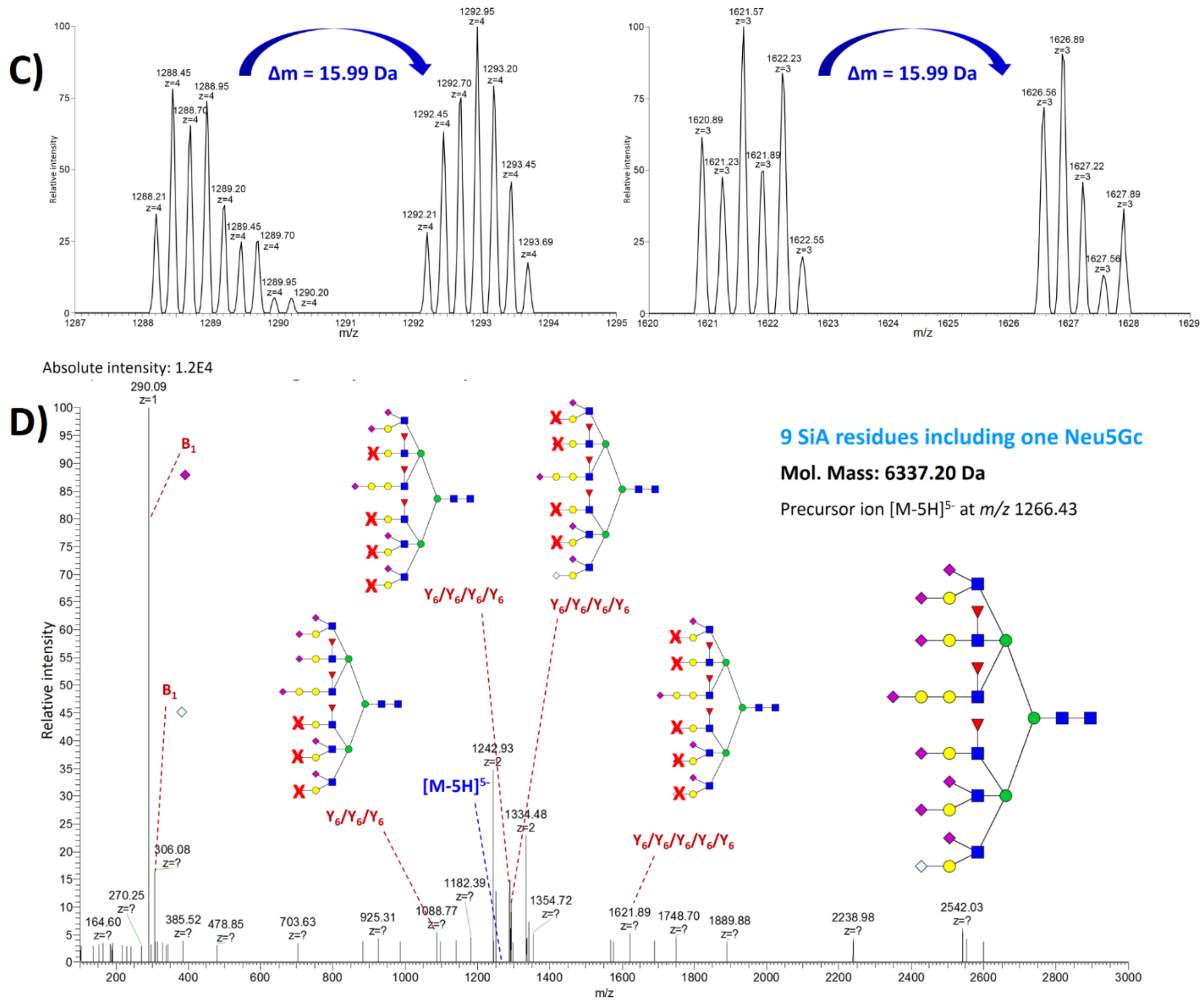

Supplementary Figure 21: Examples of characteristic MS^2 spectra in negative ion mode of **heavily sialylated N-glycans containing several Neu5Ac residues and one Neu5Gc residue**. Putative structures were proposed based on the fragmentation patterns of these large size glycans. For MS^2-based structural characterization of these sialylated N-glycans the quintuply-charged molecular ions at *m/z* 1,149.99 (A), 1,208.21 (B), and 1,266.43 (D), respectively, were selected as precursor ions. The panel C) is a zoom of panel B) in the *m/z* 1,287-1,295 and *m/z* 1,620-1,629 ranges to show the characteristic mass shift of 15.99 Da between the isotopic distributions of closely-related fragment ions, which differ only by one oxygen atom due to the presence of one Neu5Gc residue. Purple diamond, Neu5Ac; light blue diamond, Neu5Gc. The red cross symbol relates to the loss of the selected monosaccharide.

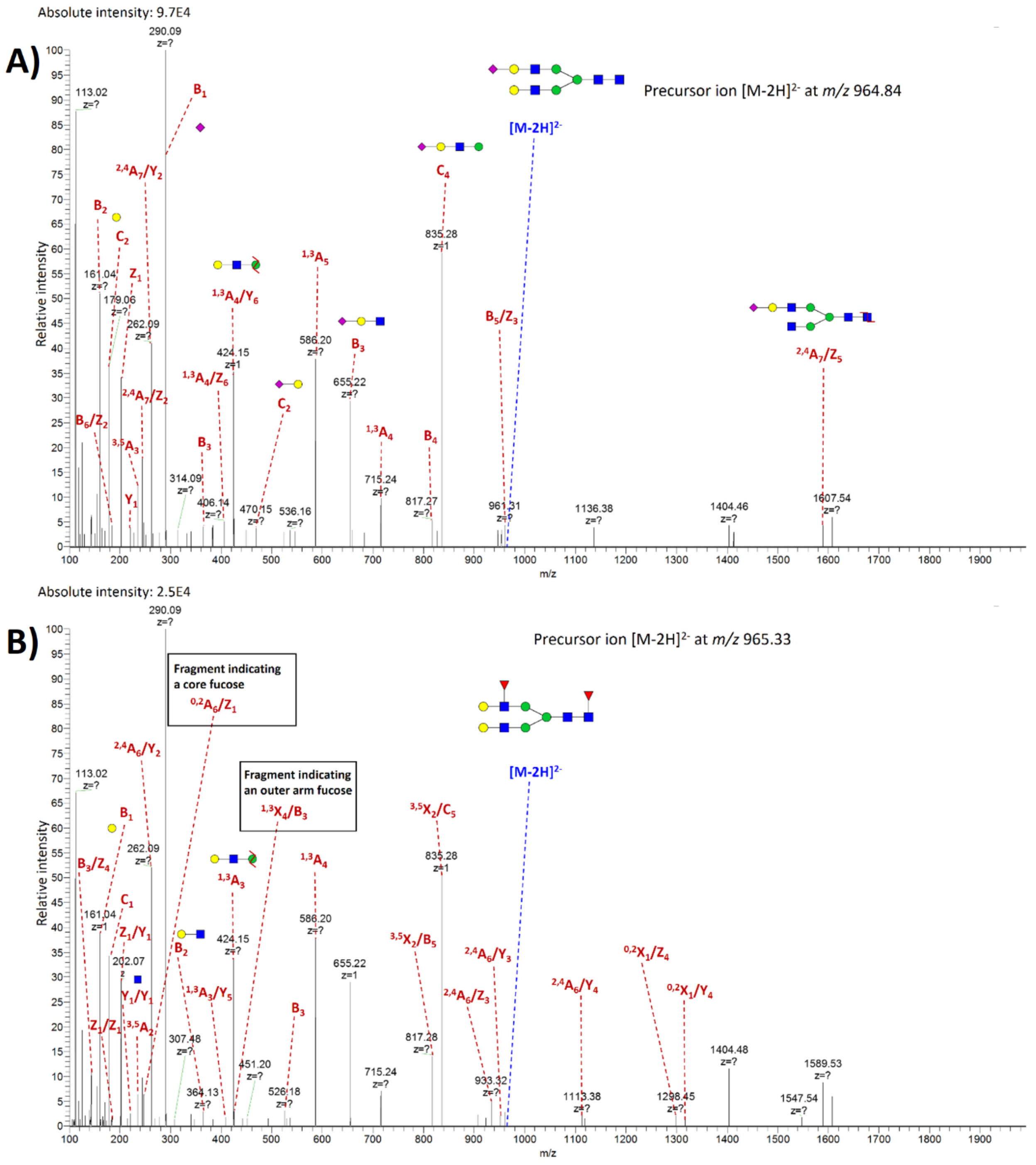

Supplementary Figure 22: Characteristic MS^2 spectra of the monosialylated N-glycan **A2G2S1** and the difucosylated N-glycan **FA2F1G2**. The doubly-charged molecular ions at m/z 964.84, and 965.33, respectively, were selected as precursor ions.

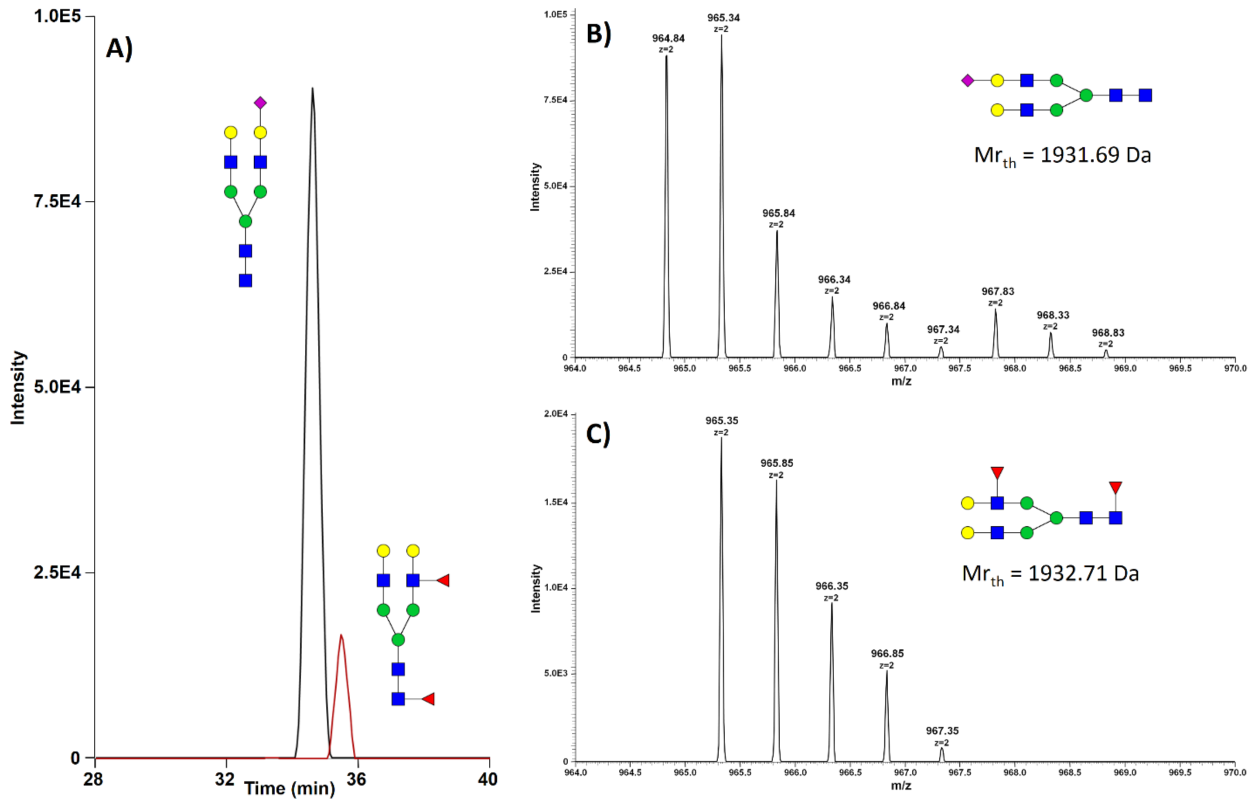

Supplementary Figure 23: An example of **separation of N-glycans with similar mass** is demonstrated for label-free CZE-MS analysis of N-glycans released from bovine serum fetuin. A) **EIEs of A2G2S1 and FA2F1G2** (at *m/z* 964.8360 ± 0.0019 and *m/z* 965.3462 ± 0.0019 (i.e., ± 2 ppm), respectively), the molecular mass of which differs by ~1.02 Da. B) and C) Mass spectra integrated across the separated peaks shown in panel A). The neutral difucosylated glycan migrates later than the monosialylated glycan (see Supplementary Figure 22 for the fragmentation patterns of these glycans).

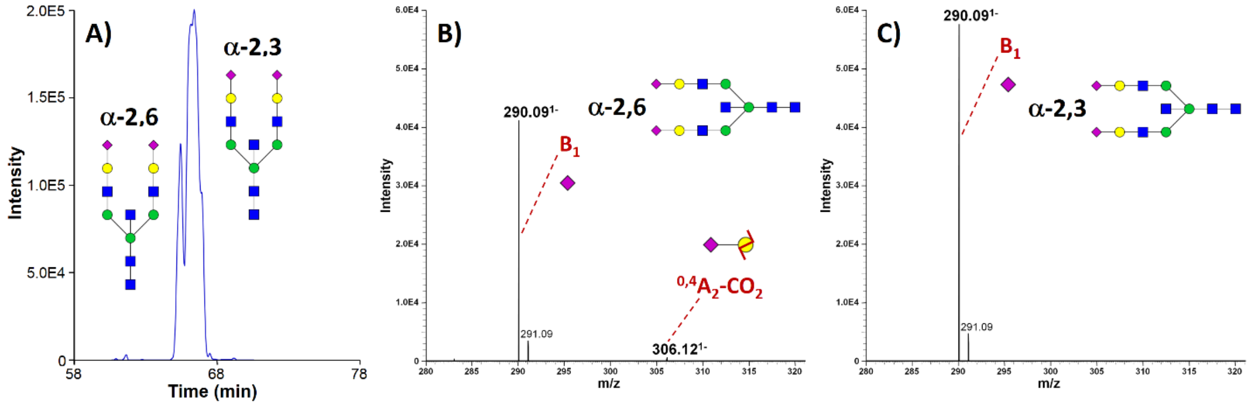

Supplementary Figure 24: CZE-MS-based **separation of A2BG2S2 sialic acid linkage isomers** released from the BSF isolate. A) EIE of A2BG2S2 glycan. B) and C) MS^2 spectra of α-2-6- and

α-2-3-linked sialic acid isomers, respectively, showing the presence or absence of the α-2-6 linkage diagnostic ion $^{0,4}A_2$-CO_2^{1-} at *m/z* 306.12.

Supplementary Note 9

The developed CZE-MS^2 technique made possible the detection and characterization of neutral N-glycans in the fetuin isolate, e.g., the bi-antennary fucosylated glycans **FA2G2** (see **Supplementary Figure 9B**) and **FA2G3**. **Supplementary Figure 25** depicts the fragmentation pattern of FA2G3 (Mr_{th} 1,948.70 Da). For this glycan, the $[M-2H]^{2-}$ molecular ion at *m/z* 973.34 was selected as a precursor ion. The diagnostic ion B_5/Z_3^{1-} (*m/z* 670.22), detected at high abundance, confirmed the composition of the 6-linked antenna, and its internal fragmentation yielded the predominant $B_5/Z_3/Y_4^{1-}$ (*m/z* 305.08) fragment ion.

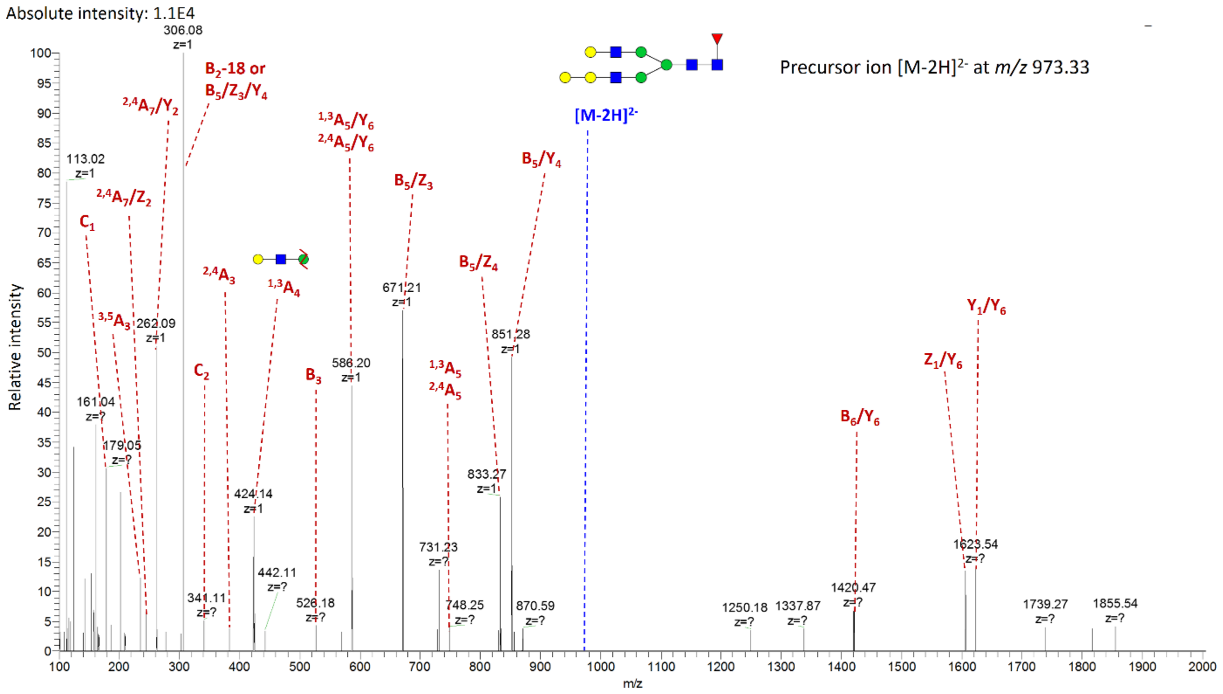

Supplementary Figure 25: Example of characteristic MS^2 spectrum of **FA2G3**. The doubly-charged molecular ion at *m/z* 973.33 was selected as precursor ion.

Supplementary Note 10

For the structural characterization of Man13, the $[M-2H]^{2-}$ molecular ion at *m/z* 1,264.42 was selected as a precursor ion (**Supplementary Figure 26**). The highest intensity fragment ions detected in the mass spectra of Man13 were the C_1^{1-} (*m/z* 179.05) and B_2^{1-} (*m/z* 323.09) ions, corresponding to the loss of one and two hexose residues from the non-reducing end, respectively [10]. The ion $^{2,4}A_7/Y_3^{1-}$ (*m/z* 869.26) was also detected at high abundance and indicated a cross-ring cleavage of one GlcNAc residue of the chitobiose core. Other fragment ions were supportive of a branched oligomannosyl structure, e.g., C_3^{1-} (*m/z* 503.15) and C_7/Y_3^{1-} (*m/z* 1031.31) ions.

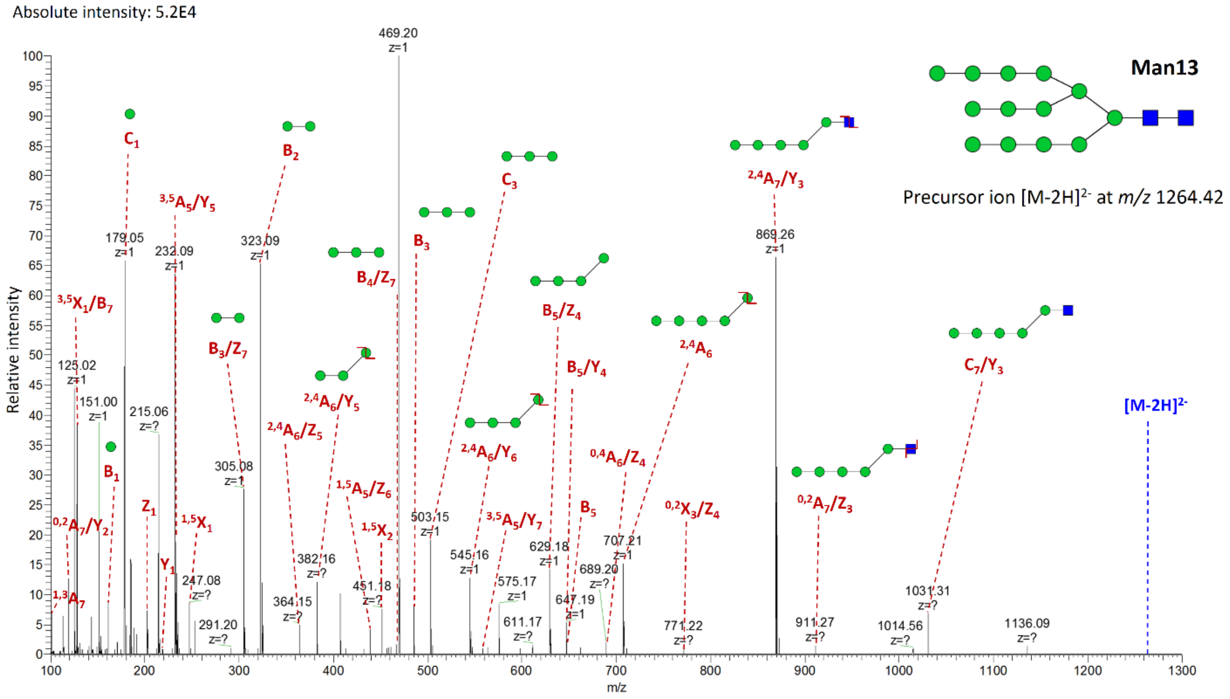

Supplementary Figure 26: Example of characteristic MS^2 spectrum of **Man13**. The doubly-charged molecular ion at *m/z* 1,264.42 was selected as precursor ion.

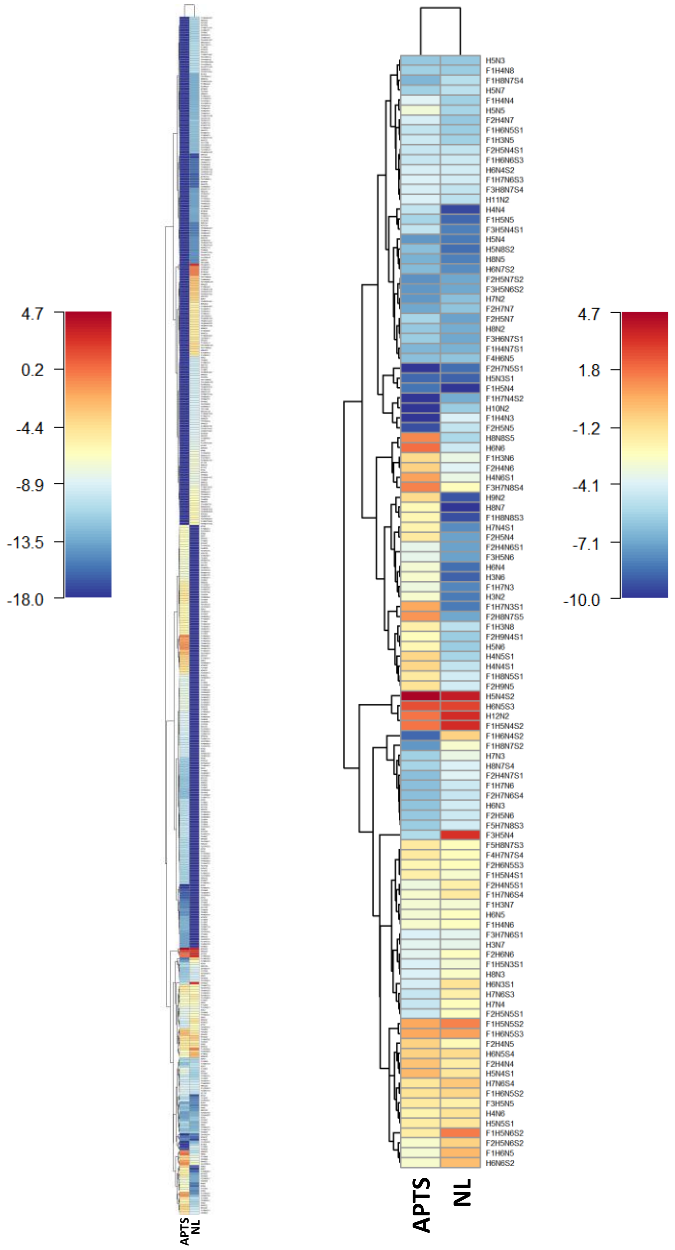

Supplementary Figure 27: Comparison of label-free and APTS-labeling approaches in the CZE-MS-based N-glycan profiling of human plasma EVs. Euclidean-based hierarchical clustering of quantitative profiles of N-glycan detected in human plasma EVs with the APTS-labeling and label-free CZE-MS methods using injected sample amounts equivalent to ~340 nL and ~185 nL of human plasma, respectively (NL stands for non-labeled). The glycans commonly detected using both strategies are shown on the right panel. Red, yellow, and light blue colors correspond to high, medium, and low relative abundances based on the N-glycan signal intensities.

Supplementary References:

1 Danielson, K. M. *et al.* Diurnal Variations of Circulating Extracellular Vesicles Measured by Nano Flow Cytometry. *PLoS One* **11**, e0144678, doi:10.1371/journal.pone.0144678 (2016).

2 Takov, K., Yellon, D. M. & Davidson, S. M. Comparison of small extracellular vesicles isolated from plasma by ultracentrifugation or size-exclusion chromatography: yield, purity and functional potential. *J Extracell Vesicles* **8**, 1560809, doi:10.1080/20013078.2018.1560809 (2019).

3 Marie, A. L. *et al.* High-Sensitivity Glycan Profiling of Blood-Derived Immunoglobulin G, Plasma, and Extracellular Vesicle Isolates with Capillary Zone Electrophoresis-Mass Spectrometry. *Anal Chem* **93**, 1991-2002, doi:10.1021/acs.analchem.0c03102 (2021).

4 Klein, J., Carvalho, L. & Zaia, J. Application of network smoothing to glycan LC-MS profiling. *Bioinformatics* **34**, 3511-3518, doi:10.1093/bioinformatics/bty397 (2018).

5 Harvey, D. J., Bateman, R. H. & Green, M. R. High-energy collision-induced fragmentation of complex oligosaccharides ionized by matrix-assisted laser desorption/ionization mass spectrometry. *J Mass Spectrom* **32**, 167-187, doi:10.1002/(SICI)1096-9888(199702)32:2<167::AID-JMS472>3.0.CO;2-Q (1997).

6 Lim, M. S. *et al.* Validation of Rapi-Fluor method for glycan profiling and application to commercial antibody drugs. *Talanta* **198**, 105-110, doi:10.1016/j.talanta.2019.01.093 (2019).

7 Mittermayr, S., Bones, J., Doherty, M., Guttman, A. & Rudd, P. M. Multiplexed analytical glycomics: rapid and confident IgG N-glycan structural elucidation. *J Proteome Res* **10**, 3820-3829, doi:10.1021/pr200371s (2011).

8 Sagi, D., Peter-Katalinic, J., Conradt, H. S. & Nimtz, M. Sequencing of tri- and tetraantennary N-glycans containing sialic acid by negative mode ESI QTOF tandem MS. *J Am Soc Mass Spectrom* **13**, 1138-1148, doi:10.1016/S1044-0305(02)00412-9 (2002).

9 Szabo, Z. *et al.* In-depth analyses of native N-linked glycans facilitated by high-performance anion exchange chromatography-pulsed amperometric detection coupled to mass spectrometry. *Anal Bioanal Chem* **409**, 3089-3101, doi:10.1007/s00216-017-0248-3 (2017).

10 Harvey, D. J. & Abrahams, J. L. Fragmentation and ion mobility properties of negative ions from N-linked carbohydrates: Part 7. Reduced glycans. *Rapid Commun Mass Spectrom* **30**, 627-634, doi:10.1002/rcm.7467 (2016).
