# Supplementary material for: Highly-sensitive label-free deep profiling of N-glycans released from biomedically-relevant samples
Source: Nat Commun. 2023 Mar 23;14:1618. doi: 10.1038/s41467-023-37365-4 (PMC10036494; doi:10.1038/s41467-023-37365-4)
Supplement: Supplementary file 2 — Description of Additional Supplementary Files [file 41467_2023_37365_MOESM2_ESM.docx]

**Description of Additional Supplementary Files**

File name: **Supplementary Data 1**

Description: Euclidean-based hierarchical clustering of quantitative profiles of N-glycan detected in human serum IgG with the APTS-labeling and label-free CZE-MS methods using injected sample amounts equivalent to ~3 nL of serum. The order of the glycans listed in Supplementary Data 1 corresponds to the circular heatmap in Supplementary Figure 4D.

File name: **Supplementary Data 2**

Description: List of the glycan structures characterized by MS^2^ in label-free CZE-MS^2^-based glycan profiling of IgG-derived N-glycans in an injected volume corresponding to ~3 nL of total human serum.

File name: **Supplementary Data 3**

Description: Experimental fragments detected for non-labeled **(A)** and APTS-labeled **(B)** FA2G2S2 glycan.

File name: **Supplementary Data 4**

Description: Experimental fragments detected for non-labeled A3G3S3 glycan.

File name: **Supplementary Data 5**

Description: Experimental fragments detected for non-labeled A3G3S5 glycan.

File name: **Supplementary Data 6**

Description: List of the glycan structures characterized by MS^2^ in label-free CZE-MS^2^-based glycan profiling of BSF-derived N-glycans in an injected volume corresponding to ~10 nL of total bovine serum.

File name: **Supplementary Data 7**

Description: List of the glycan structures characterized by MS^2^ in label-free CZE-MS^2^-based glycan profiling of RNase B-derived N-glycans in injected amounts equivalent to ~15 ng of total protein.

File name: **Supplementary Data 8**

Description: List of the glycan structures characterized by MS^2^ in label-free CZE-MS^2^-based glycan profiling of EV-derived N-glycans in an injected volume corresponding to ~185 nL of total human plasma.

File name: **Supplementary Data 9**

Description: Euclidean-based hierarchical clustering of quantitative profiles of N-glycan detected in human plasma EVs with the APTS-labeling and label-free CZE-MS methods using injected sample amounts equivalent to ~340 nL and ~185 nL of plasma. The order of the glycans listed in Supplementary Data 9 corresponds to the linear heatmap in Supplementary Figure 27.

File name: **Supplementary Data 10**

Description: Normalized signal intensities derived from the hierarchical clustering of quantitative profiles of N-glycans detected in human serum IgG, bovine serum fetuin, bovine pancreas ribonuclease B, human plasma EV, and total human plasma isolates (see Figure 2J). The order of the glycans listed in Supplementary Data 10 corresponds to the circular heatmap in Figure 2J. Red, yellow, and light blue colors correspond to high, medium, and low relative abundances based on the N-glycan signal intensities. N-glycans that are not detected in the samples are highlighted in dark blue. F, H, N, S, and G stand for fucose, hexose, HexNAc, Neu5Ac, and Neu5Gc, respectively.
