# Supplementary material for: Highly-sensitive label-free deep profiling of N-glycans released from biomedically-relevant samples
Source: Nat Commun. 2023 Mar 23;14:1618. doi: 10.1038/s41467-023-37365-4 (PMC10036494; doi:10.1038/s41467-023-37365-4)
Supplement: Supplementary file 4 — Supplementary Data 2 [file 41467_2023_37365_MOESM4_ESM.pdf]

Human serum IgG

Composition	Name	Structure	Mr_{th} (Da)
Hex3HexNAc3	**H3N3**	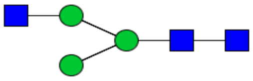	1113.4072
Hex3HexNAc3	**H3N3**	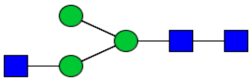	1113.4072
Hex3HexNAc3	**H3N3**	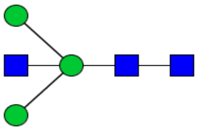	1113.4072
Hex4HexNAc3	**H4N3**	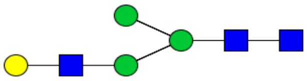	1275.4600
Hex3HexNAc4	**H3N4**	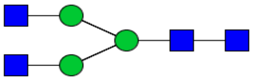	1316.4865
Hex4HexNAc4	**H4N4**	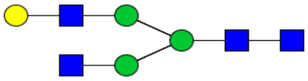	1478.5393
Hex4HexNAc4	**H4N4**	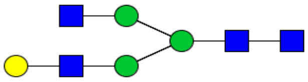	1478.5393
Hex4HexNAc4	**H4N4**	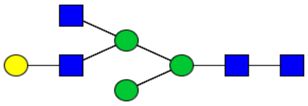	1478.5393
Hex3HexNAc5	**H3N5**	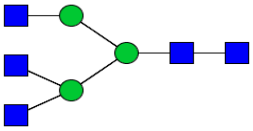	1519.5659
Hex5HexNAc4	**H5N4**	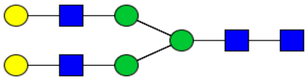	1640.5922

Hex5HexNAc4	**H5N4**	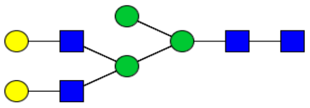	1640.5922
Hex4HexNAc5	**H4N5**	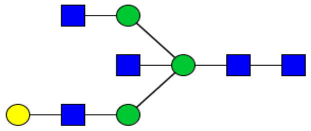	1681.6187
Hex3HexNAc6	**H3N6**	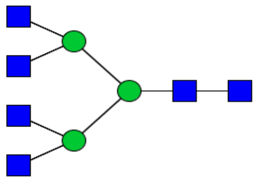	1722.6453
Hex6HexNAc4	**H6N4**	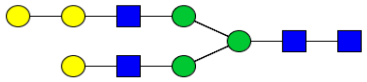	1802.6450
Hex5HexNAc5	**H5N5**	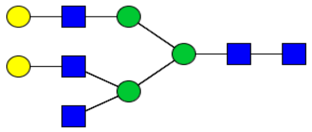	1843.6715
Hex5HexNAc5	**H5N5**	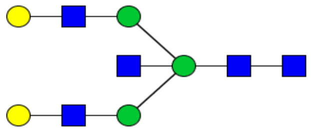	1843.6715
Hex3HexNAc7	**H3N7**	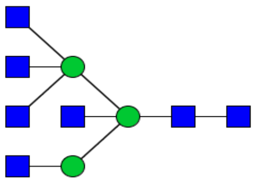	1925.7246
Hex5HexNAc6	**H5N6**	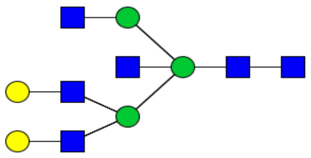	2046.7509

Hex4HexNAc7	**H4N7**	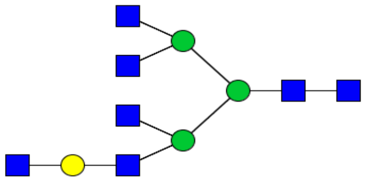	2087.7775
Hex6HexNAc6	**H6N6**	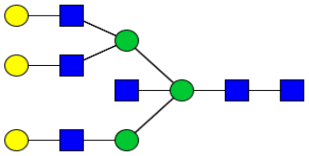	2208.8037
Hex4HexNAc8	**H4N8**	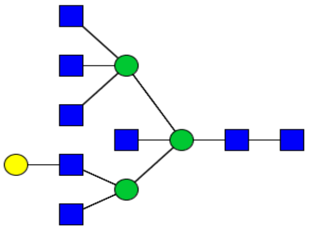	2290.8568
Hex8HexNAc5	**H8N5**	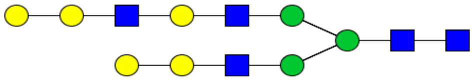	2329.8300
Hex7HexNAc6	**H7N6**	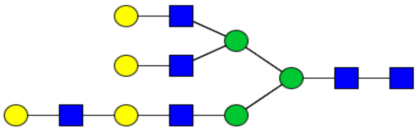	2370.8566
Hex5HexNAc8	**H5N8**	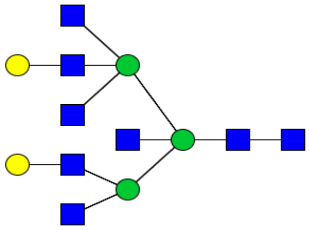	2452.9097
Hex9HexNAc5	**H9N5**	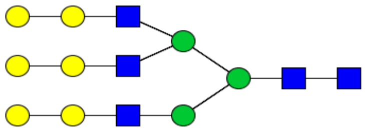	2491.8828

Hex7HexNAc7	**H7N7**	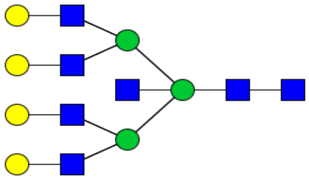	2573.9359
Hex7HexNAc8	**H7N8**	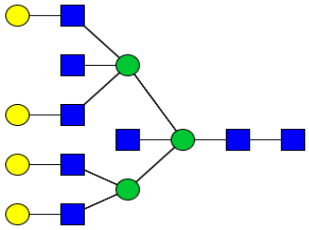	2777.0153
Hex4HexNAc4Neu5Ac1	**H4N4S1**	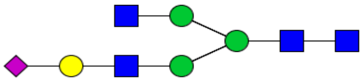	1769.6348
Hex5HexNAc4Neu5Ac1	**H5N4S1**	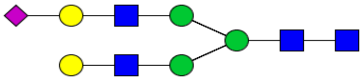	1931.6876
Hex6HexNAc4Neu5Ac1	**H6N4S1**	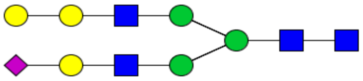	2093.7404
Hex5HexNAc5Neu5Ac1	**H5N5S1**	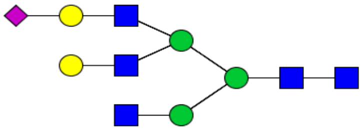	2134.7670
Hex6HexNAc5Neu5Ac1	**H6N5S1**	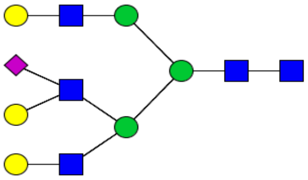	2296.8198
Hex7HexNAc6Neu5Ac1	**H7N6S1**	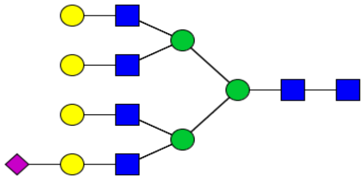	2661.9520

Fuc1Hex4HexNAc3Neu5Ac1	**F1H4N3S1**	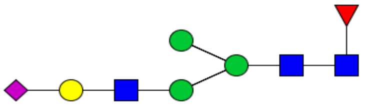	1712.6133
Fuc1Hex4HexNAc4Neu5Ac1	**F1H4N4S1**	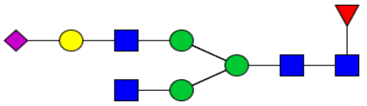	1915.6927
Fuc1Hex4HexNAc4Neu5Ac1	**F1H4N4S1**	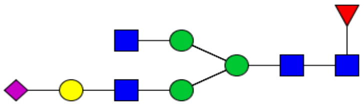	1915.6927
Fuc1Hex5HexNAc4Neu5Ac1	**F1H5N4S1**	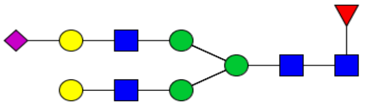	2077.7455
Fuc1Hex5HexNAc4Neu5Ac1	**F1H5N4S1**	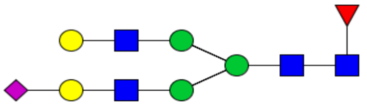	2077.7455
Fuc1Hex4HexNAc5Neu5Ac1	**F1H4N5S1**	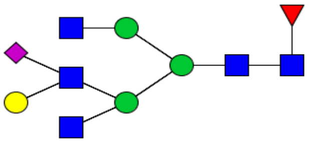	2118.7720
Fuc1Hex5HexNAc5Neu5Ac1	**F1H5N5S1**	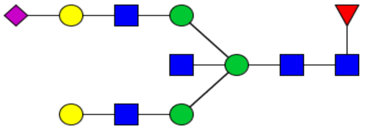	2280.8249
Fuc1Hex6HexNAc5Neu5Ac1	**F1H6N5S1**	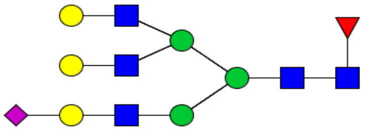	2442.8777
Fuc1Hex7HexNAc6Neu5Ac1	**F1H7N6S1**	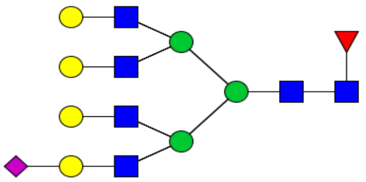	2808.0099

Fuc2Hex4HexNAc4Neu5Ac1	**F2H4N4S1**	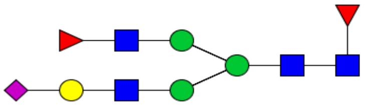	2061.7506
Fuc2Hex5HexNAc4Neu5Ac1	**F2H5N4S1**	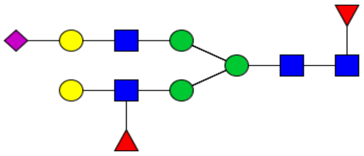	2223.8034
Fuc2Hex6HexNAc5Neu5Ac1	**F2H6N5S1**	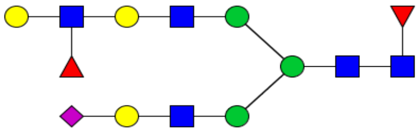	2588.9356
Fuc3Hex5HexNAc4Neu5Ac1	**F3H5N4S1**	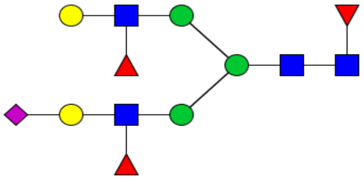	2369.8613
Fuc3Hex6HexNAc5Neu5Ac1	**F3H6N5S1**	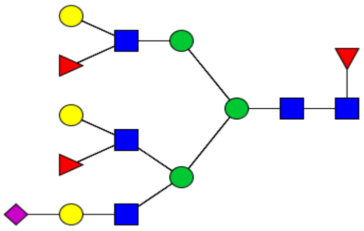	2734.9935
Hex4HexNAc4Neu5Ac2	**H4N4S2**	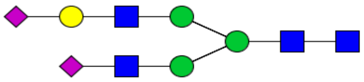	2060.7302
Hex5HexNAc4Neu5Ac2	**H5N4S2**	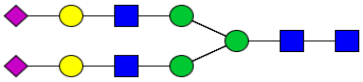	2222.7830
Hex6HexNAc4Neu5Ac2	**H6N4S2**	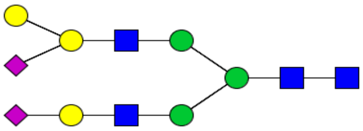	2384.8358
Hex5HexNAc5Neu5Ac2	**H5N5S2**	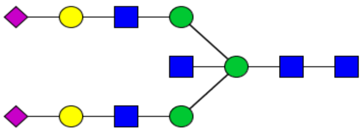	2425.8624

Hex6HexNAc5Neu5Ac2	**H6N5S2**	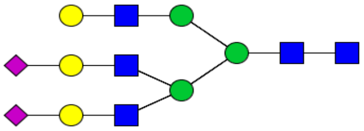	2587.9152
Hex6HexNAc5Neu5Ac2	**H6N5S2**	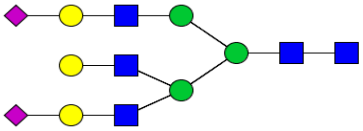	2587.9152
Hex6HexNAc6Neu5Ac2	**H6N6S2**	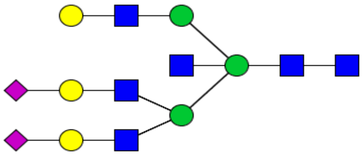	2790.9946
Fuc1Hex5HexNAc4Neu5Ac2	**F1H5N4S2**	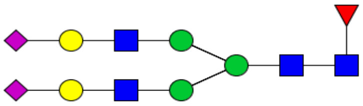	2368.8409
Fuc1Hex4HexNAc5Neu5Ac2	**F1H4N5S2**	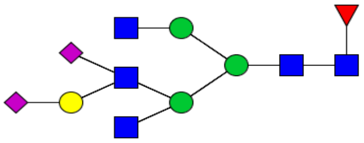	2409.8675
Fuc1Hex6HexNAc4Neu5Ac2	**F1H6N4S2**	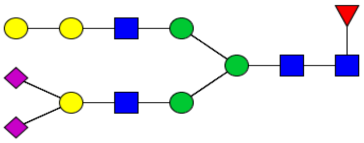	2530.8937
Fuc1Hex5HexNAc5Neu5Ac2	**F1H5N5S2**	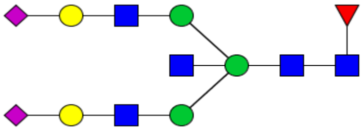	2571.9203
Fuc1Hex5HexNAc5Neu5Ac2	**F1H5N5S2**	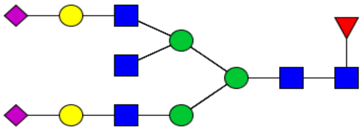	2571.9203
Fuc1Hex6HexNAc5Neu5Ac2	**F1H6N5S2**	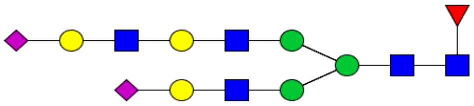	2733.9731

Fuc1Hex9HexNAc6Neu5Ac2	**F1H9N6S2**	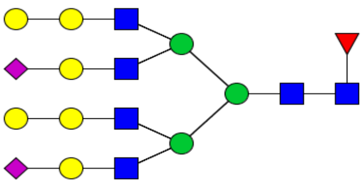	3423.2110
Fuc2Hex5HexNAc5Neu5Ac2	**F2H5N5S2**	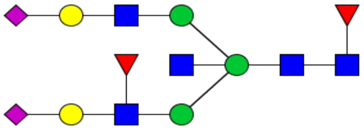	2717.9782
Fuc3Hex5HexNAc4Neu5Ac2	**F3H5N4S2**	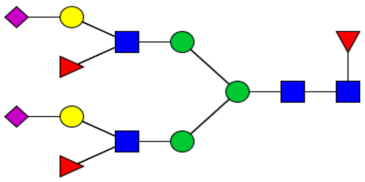	2660.9567
Hex5HexNAc4Neu5Ac3	**H5N4S3**	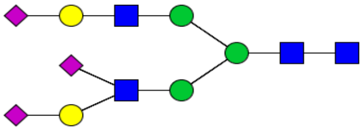	2513.8784
Hex6HexNAc5Neu5Ac3	**H6N5S3**	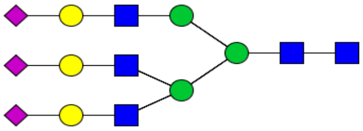	2879.0106
Fuc1Hex5HexNAc4Neu5Ac3	**F1H5N4S3**	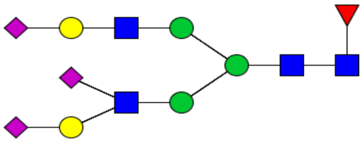	2659.9363
Fuc1Hex6HexNAc5Neu5Ac3	**F1H6N5S3**	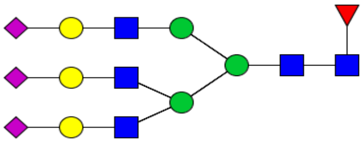	3025.0685
Fuc1Hex6HexNAc5Neu5Ac1	**F1H6N5S3**	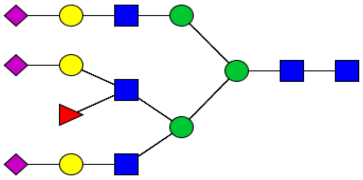	3025.0685

Fuc1Hex6HexNAc6Neu5Ac3	**F1H6H6S3**	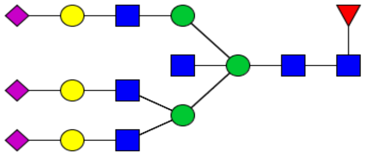	3228.1479
Fuc1Hex7HexNAc6Neu5Ac3	**F1H7H6S3**	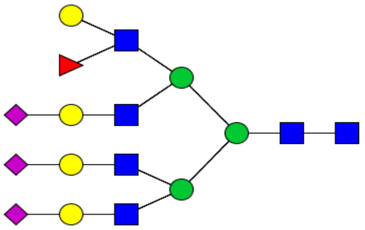	3390.2007
Fuc3Hex6HexNAc6Neu5Ac3	**F3H6N6S3**	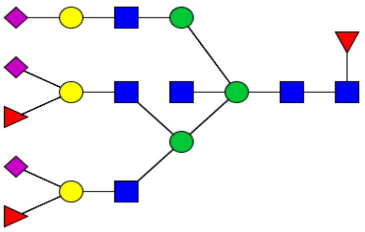	3520.2637
Hex6HexNAc5Neu5Ac4	**H6N5S4**	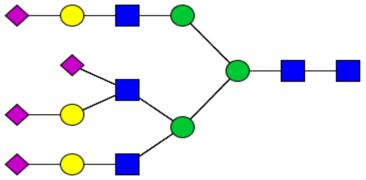	3170.1060
Hex7HexNAc6Neu5Ac4	**H7N6S4**	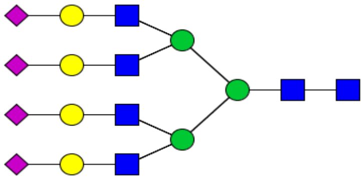	3535.2382
Hex10HexNAc9Neu5Ac4	**F1H10N9S4**	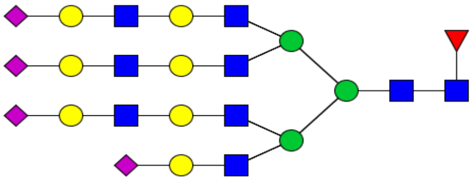	4776.6927
Fuc1Hex3HexNAc3	**F1H3N3**	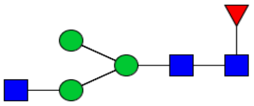	1259.4651

Fuc1Hex4HexNAc3	**F1H4N3**	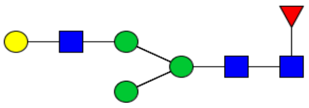	1421.5179
Fuc1Hex3HexNAc4	**F1H3N4**	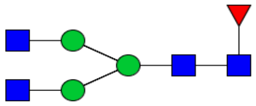	1462.5444
Fuc1Hex3HexNAc4	**F1H3N4**	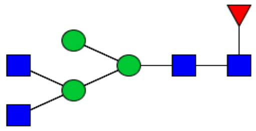	1462.5444
Fuc1Hex4HexNAc4	**F1H4N4**	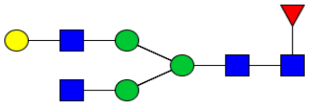	1624.5973
Fuc1Hex4HexNAc4	**F1H4N4**	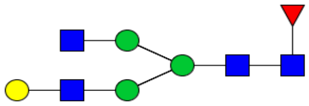	1624.5973
Fuc1Hex4HexNAc4	**F1H4N4**	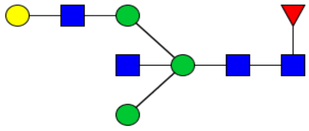	1624.5973
Fuc1Hex3HexNAc5	**F1H3N5**	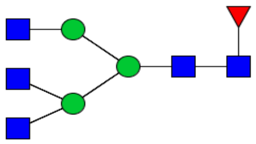	1665.6238
Fuc1Hex5HexNAc4	**F1H5N4**	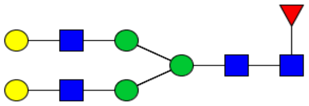	1786.6501
Fuc1Hex5HexNAc4	**F1H5N4**	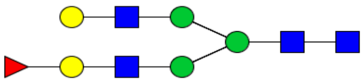	1786.6501
Fuc1Hex5HexNAc4	**F1H5N4**	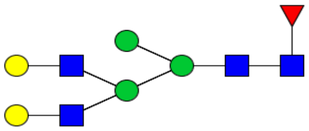	1786.6501

Fuc1Hex5HexNAc4	**F1H5N4**	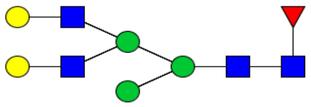	1786.6501
Fuc1Hex4HexNAc5	**F1H4N5**	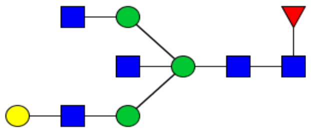	1827.6766
Fuc1Hex4HexNAc5	**F1H4N5**	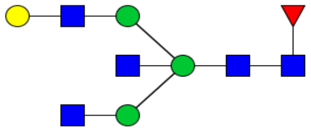	1827.6766
Fuc1Hex4HexNAc5	**F1H4N5**	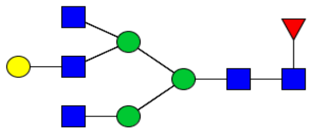	1827.6766
Fuc1Hex4HexNAc5	**F1H4N5**	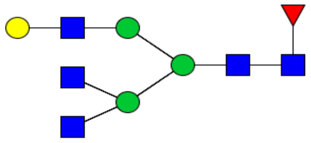	1827.6766
Fuc1Hex4HexNAc5	**F1H4N5**	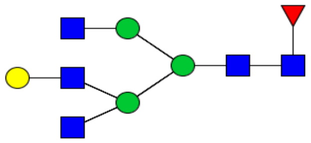	1827.6766
Fuc1Hex6HexNAc4	**F1H6N4**	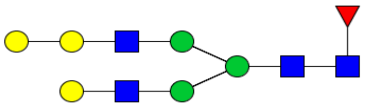	1948.7029
Fuc1Hex5HexNAc5	**F1H5N5**	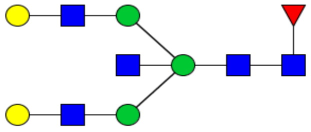	1989.7295
Fuc1Hex5HexNAc5	**F1H5N5**	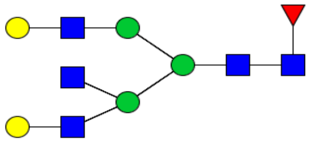	1989.7295

Fuc1Hex4HexNAc6	**F1H4N6**	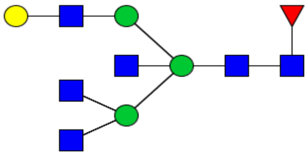	2030.7560
Fuc1Hex3HexNAc7	**F1H3N7**	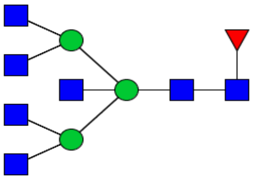	2071.7826
Fuc1Hex7HexNAc4	**F1H7N4**	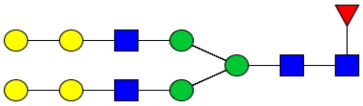	2110.7557
Fuc1Hex6HexNAc5	**F1H6N5**	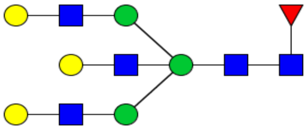	2151.7823
Fuc1Hex5HexNAc6	**F1H5N6**	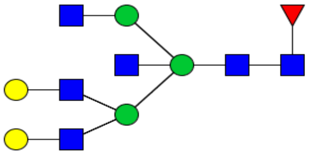	2192.8088
Fuc1Hex4HexNAc7	**F1H4N7**	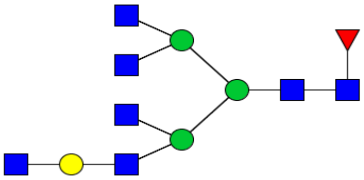	2233.8354
Fuc1Hex7HexNAc5	**F1H7N5**	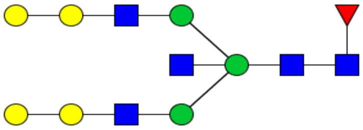	2313.8351
Fuc1Hex6HexNAc6	**F1H6N6**	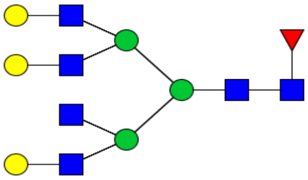	2354.8616

Fuc1Hex6HexNAc6	**F1H8N5**	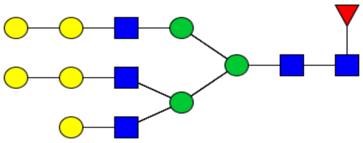	2475.8879
Fuc1Hex7HexNAc6	**F1H7N6**	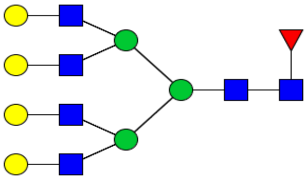	2516.9145
Fuc1Hex5HexNAc8	**F1H5N8**	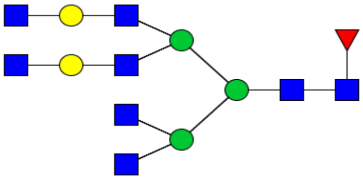	2598.9676
Fuc1Hex9HexNAc5	**F1H9N5**	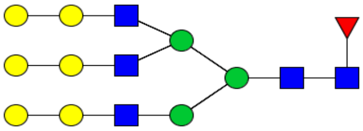	2637.9407
Fuc1Hex8HexNAc6	**F1H8N6**	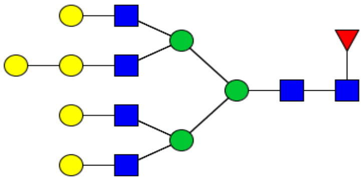	2678.9673
Fuc1Hex9HexNAc6	**F1H9N6**	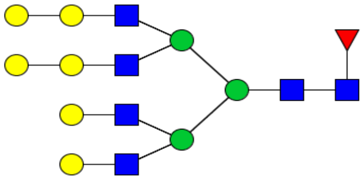	2841.0201
Fuc1Hex8HexNAc7	**F1H8N7**	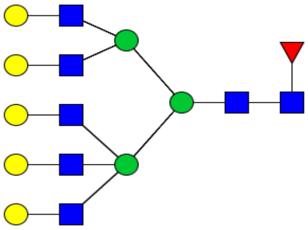	2882.0467

Composition	Short name	Structure	Mass
Fuc1Hex8HexNAc8	**F1H8N8**	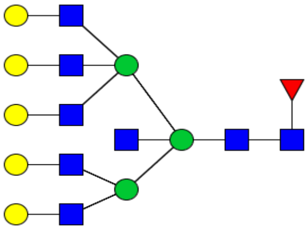	3085.1260
Fuc1Hex9HexNAc8	**F1H9N8**	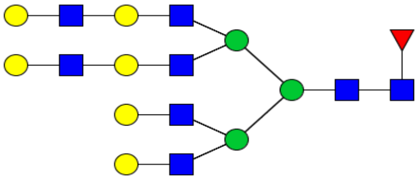	3247.1789
Fuc1Hex11HexNAc8	**F1H11N8**	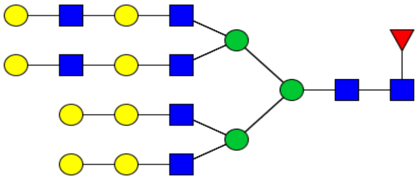	3571.2845
Fuc2Hex3HexNAc3	**F2H3N3**	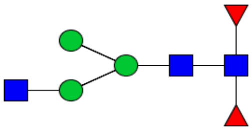	1405.5230
Fuc2Hex3HexNAc4	**F2H3N4**	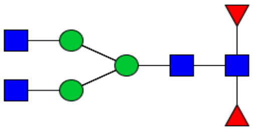	1608.6023
Fuc2Hex4HexNAc3	**F2H4N3**	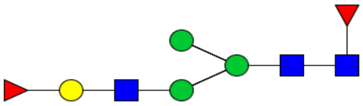	1567.5758
Fuc2Hex5HexNAc4	**F2H5N4**	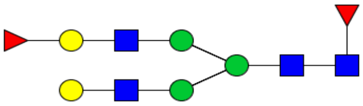	1932.7080
Fuc2Hex5HexNAc4	**F2H5N4**	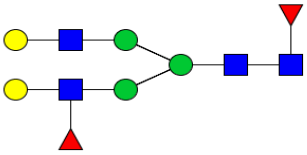	1932.7080

Fuc2Hex4HexNAc5	**F2H4N5**	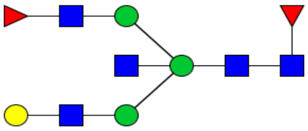	1973.7345
Fuc2Hex6HexNAc4	**F2H6N4**	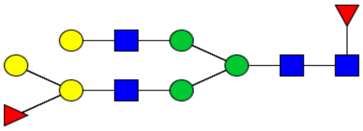	2094.7608
Fuc2Hex5HexNAc5	**F2H5N5**	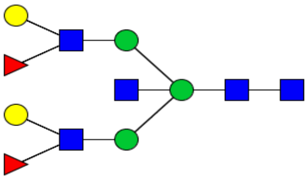	2135.7874
Fuc2Hex4HexNAc6	**F2H4N6**	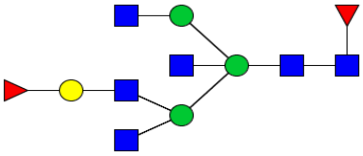	2176.8139
Fuc2Hex6HexNAc5	**F2H6N5**	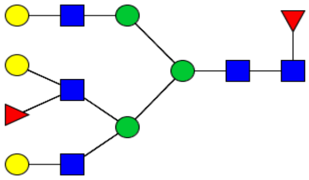	2297.8402
Fuc2Hex4HexNAc7	**F2H4N7**	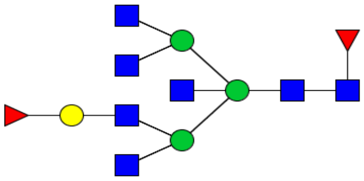	2379.8933
Fuc2Hex7HexNAc6	**F2H7N6**	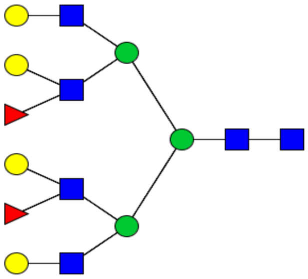	2662.9724

Fuc3Hex5HexNAc4	**F3H5N4**	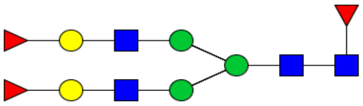	2078.7659
Fuc3Hex5HexNAc5	**F3H5N5**	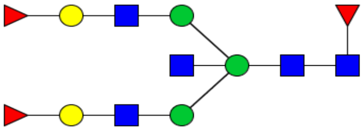	2281.8453
Fuc3Hex6HexNAc4	**F3H6N4**	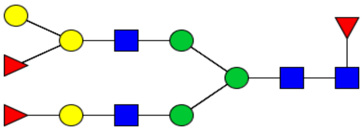	2240.8187
Fuc3Hex6HexNAc5	**F3H6N5**	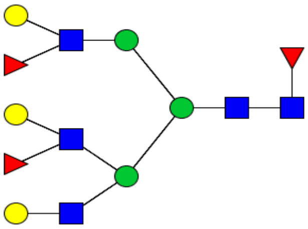	2443.8981
Fuc3Hex9HexNAc8	**F3H9N8**	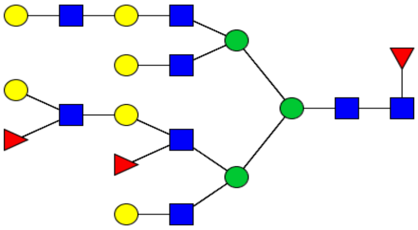	3539.2947
Fuc4Hex6HexNAc5	**F4H6N5**	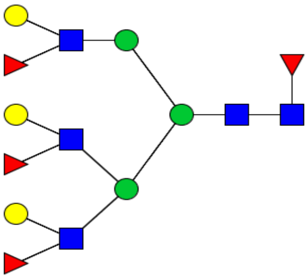	2589.9560
Fuc4Hex8HexNAc5	**F4H8N5**	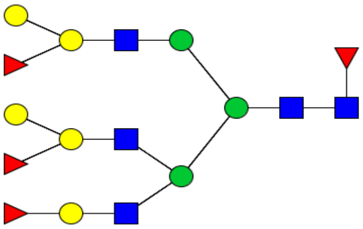	2914.0617

Hex4HexNAc2	**H4N2**	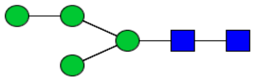	1072.3806
Hex5HexNAc2	**H5N2**	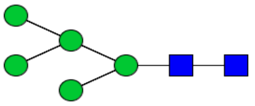	1234.4334
Hex5HexNAc2	**H5N2**	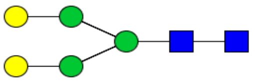	1234.4334
Hex6HexNAc2	**H6N2**	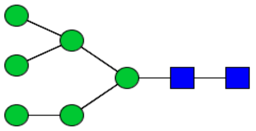	1396.4863
Hex6HexNAc2	**H6N2**	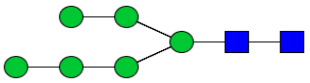	1396.4863
Hex7HexNAc2	**H7N2**	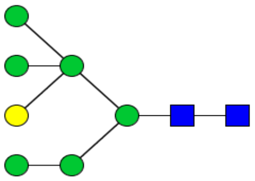	1558.5391
Hex8HexNAc2	**H8N2**	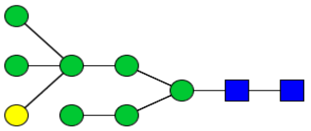	1720.5919
Hex9HexNAc2	**H9N2**	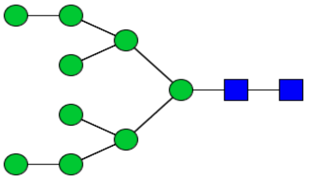	1882.6447
Hex10HexNAc2	**H10N2**	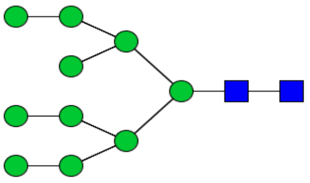	2044.6975

Hex11HexNAc2	**H11N2**	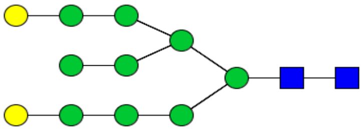	2206.7504
Fuc1Hex9HexNAc2	**F1H9N2**	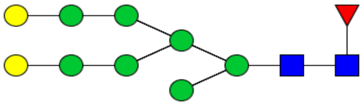	2028.7026
Hex5HexNAc3	**H5N3**	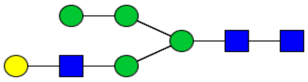	1437.5128
Hex4HexNAc4	**H4N4**	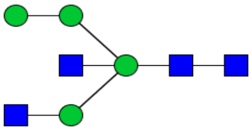	1478.5393
Hex6HexNAc3	**H6N3**	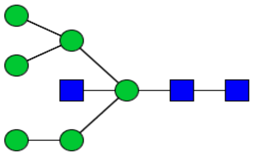	1599.5656
Hex5HexNAc4	**H5N4**	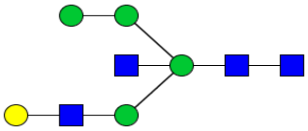	1640.5922
Hex4HexNAc6	**H4N6**	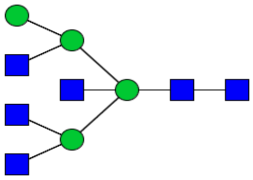	1884.6981
Hex6HexNAc5	**H6N5**	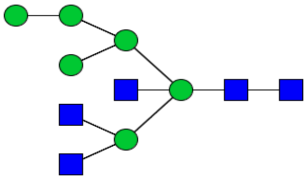	2005.7244
Hex9HexNAc3	**H9N3**	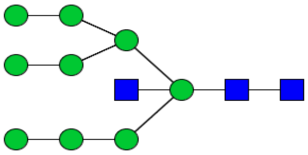	2085.7241

Hex7HexNAc5	**H7N5**	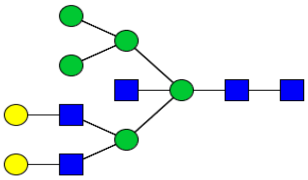	2167.7772
Hex5HexNAc4Neu5Ac1	**H5N4S1**	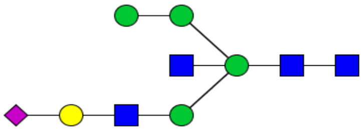	1728.6082
Hex6HexNAc3Neu5Ac1	**H6N3S1**	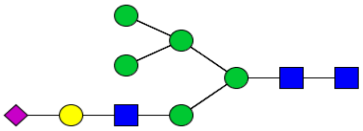	1890.6610
Fuc1Hex6HexNAc3Neu5Ac1	**F1H6N3S1**	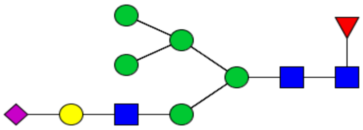	2036.7189
Fuc1Hex6HexNAc4Neu5Ac1	**F1H6N4S1**	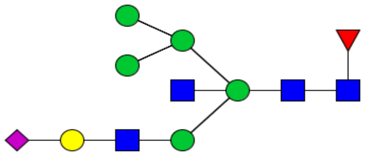	2239.7983
Fuc1Hex5HexNAc3	**F1H5N3**	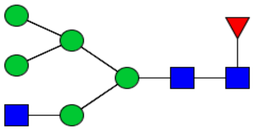	1583.5707
Fuc1Hex6HexNAc3	**F1H6N3**	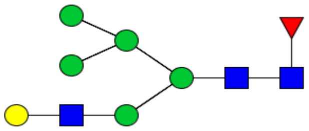	1745.6235
