# Supplementary material for: Highly-sensitive label-free deep profiling of N-glycans released from biomedically-relevant samples
Source: Nat Commun. 2023 Mar 23;14:1618. doi: 10.1038/s41467-023-37365-4 (PMC10036494; doi:10.1038/s41467-023-37365-4)
Supplement: Supplementary file 8 — Supplementary Data 6 [file 41467_2023_37365_MOESM8_ESM.pdf]

Bovine serum Fetuin

Composition	Name	Structure	Mr_{th} (Da)
Fuc3Hex10HexNAc8Neu5Ac10	**F3H10N8S10**	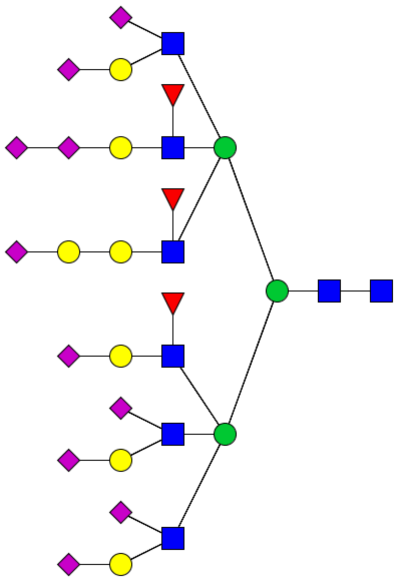	6612.3017
Fuc3Hex10HexNAc8Neu5Ac9	**F3H10N8S9**	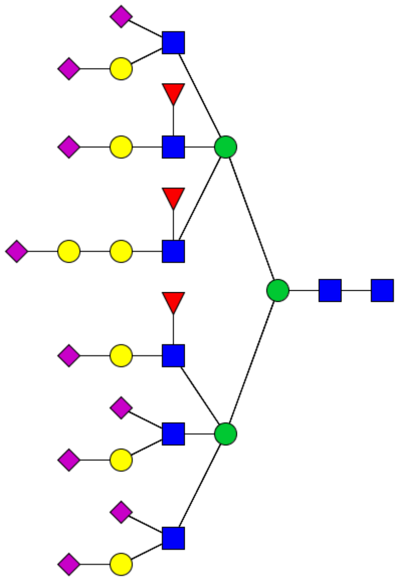	6321.2063
Fuc3Hex10HexNAc8Neu5Ac8	**F3H10N8S8**	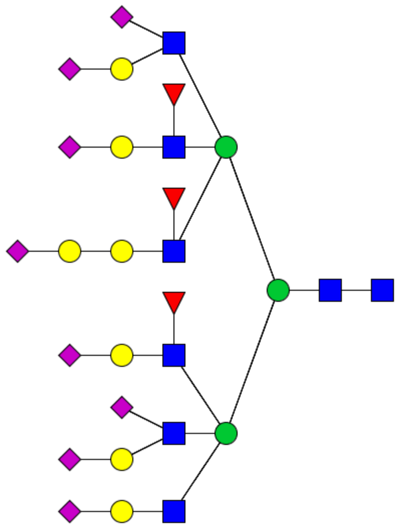	6030.1108

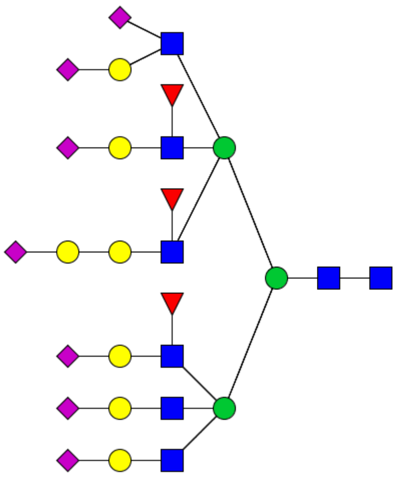

Fuc3Hex10HexNAc8Neu5Ac7 **F3H10N8S7** 5739.0154

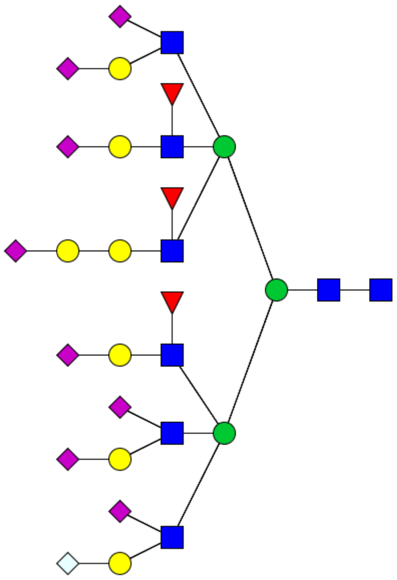

Fuc3Hex10HexNAc8Neu5Ac8Neu5Gc1 **F3H10N8S9** 6337.2012

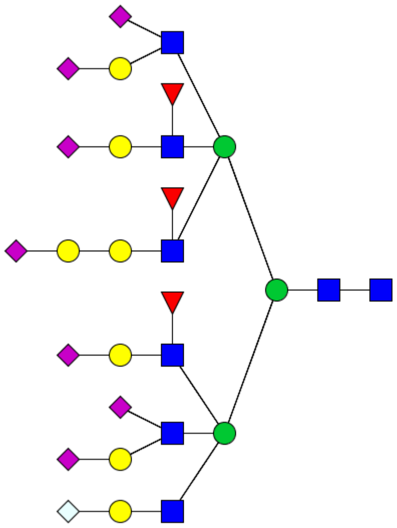

Fuc3Hex10HexNAc8Neu5Ac7Neu5Gc1 **F3H10N8S8** 6046.1058

Fuc3Hex10HexNAc8Neu5Ac6Neu5Gc1	**F3H10N8S7**	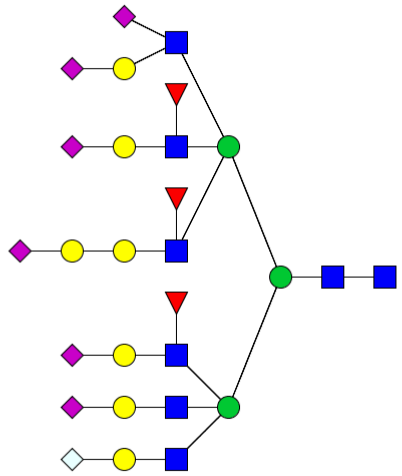	5755.0103
Hex6HexNAc5Neu5Ac6	**H6N5S6**	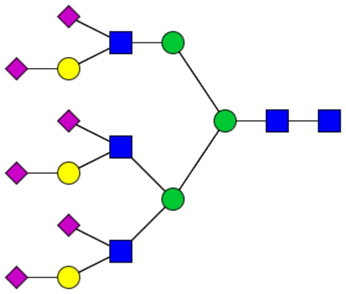	3752.2969
Hex6HexNAc5Neu5Ac5	**H6N5S5**	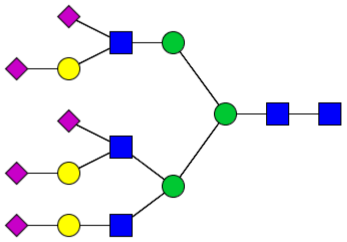	3461.2015
Fuc1Hex6HexNAc5Neu5Ac5	**F1H6N5S5**	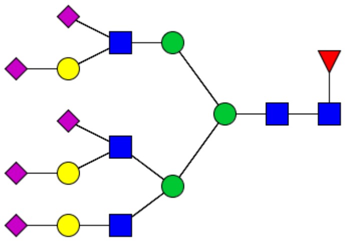	3607.2594
Hex5HexNAc4Neu5Ac4	**H5N4S4**	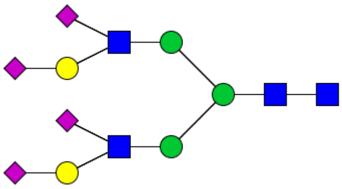	2804.9738

Hex6HexNAc5Neu5Ac4	**H6N5S4**	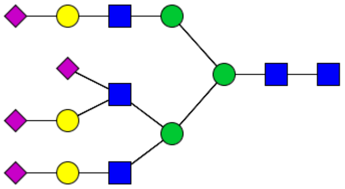	3170.1060
Hex6HexNAc5Neu5Ac4	**H6N5S4**	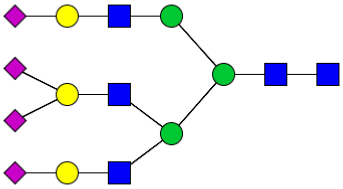	3170.1060
Hex6HexNAc5Neu5Ac4	**H6N5S4**	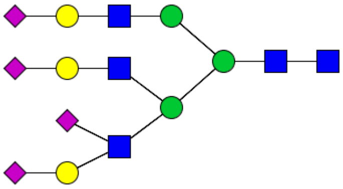	3170.1060
Hex7HexNAc6Neu5Ac4	**H7N6S4**	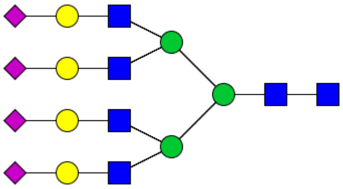	3535.2382
Fuc1Hex5HexNAc4Neu5Ac4	**F1H5N4S4**	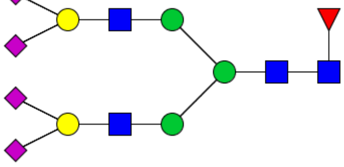	2951.0317
Fuc1Hex6HexNAc5Neu5Ac4	**F1H6N5S4**	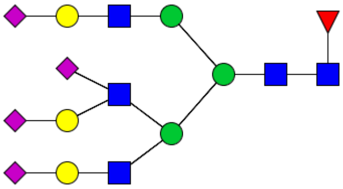	3316.1639
Fuc1Hex7HexNAc6Neu5Ac4	**F1H7N6S4**	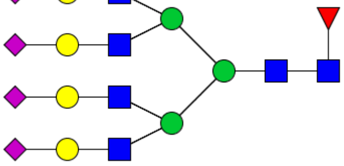	3681.2961

Fuc1Hex8HexNAc7Neu5Ac4	**F1H8N7S4**	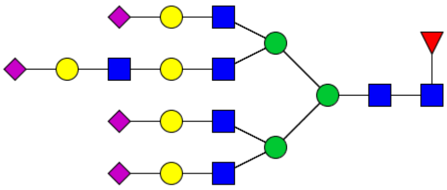	4046.4283
Fuc2Hex7HexNAc6Neu5Ac4	**F2H7N6S4**	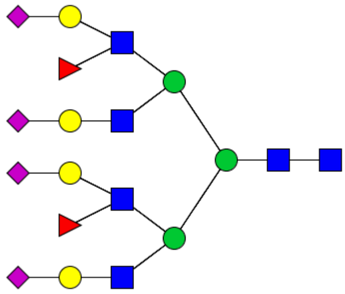	3827.3540
Fuc1Hex7HexNAc6Neu5Ac3Neu5Gc1	**F1H7N6S4**	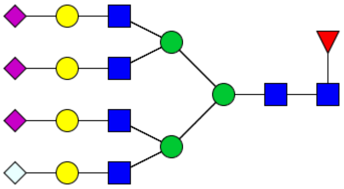	3697.2911
Hex5HexNAc4Neu5Ac3	**H5N4S3**	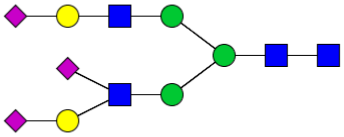	2513.8784
Hex6HexNAc5Neu5Ac3	**H6N5S3**	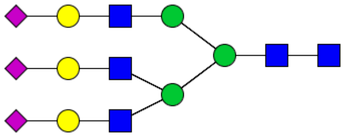	2879.0106
Hex7HexNAc6Neu5Ac3	**H7N6S3**	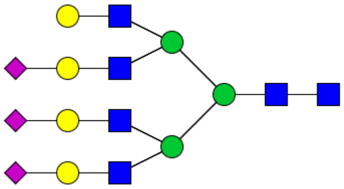	3244.1428
Fuc1Hex5HexNAc4Neu5Ac3	**F1H5N4S3**	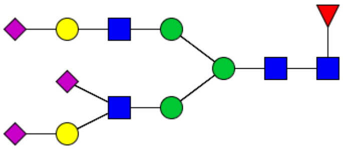	2659.9363
Fuc1Hex6HexNAc5Neu5Ac3	**F1H6N5S3**	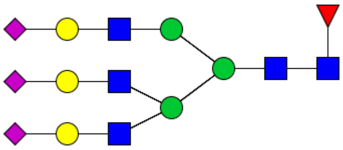	3025.0685

Fuc1Hex6HexNAc5Neu5Ac3	**F1H6N5S3**	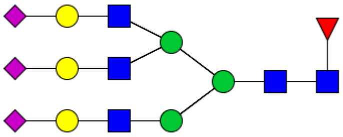	3025.0685
Fuc1Hex6HexNAc5Neu5Ac3	**F1H6N5S3**	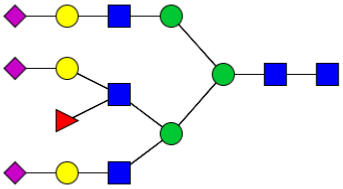	3025.0685
Fuc1Hex6HexNAc6Neu5Ac3	**F1H6N6S3**	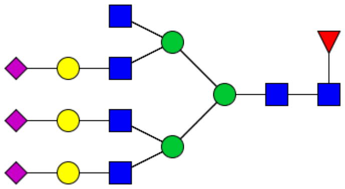	3228.1479
Fuc1Hex6HexNAc6Neu5Ac3	**F1H6N6S3**	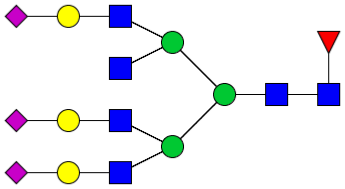	3228.1479
Fuc1Hex7HexNAc6Neu5Ac3	**F1H7N6S3**	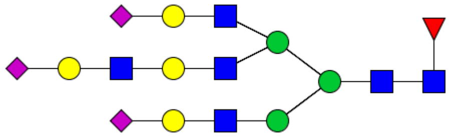	3390.2007
Fuc1Hex6HexNAc5Neu5Ac3	**F1H7N7S3**	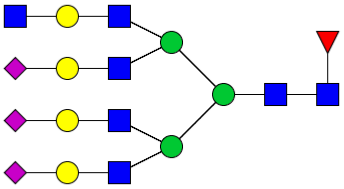	3593.2801
Fuc1Hex8HexNAc7Neu5Ac3	**F1H8N7S3**	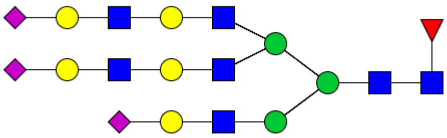	3755.3329
Fuc3Hex6HexNAc6Neu5Ac3	**F3H6N6S3**	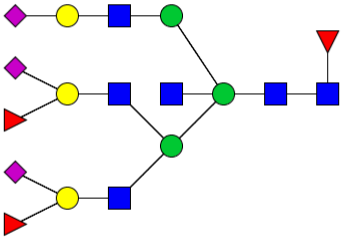	3520.2637

Fuc1Hex6HexNAc5Neu5Ac2Neu5Gc1	**F1H6N5S3**	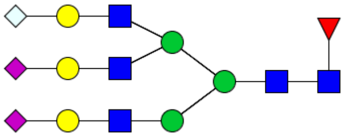	3041.0634
Fuc1Hex6HexNAc5Neu5Gc3	**F1H6N5S3**	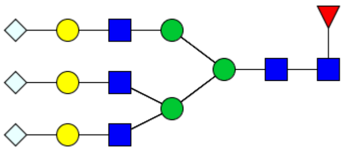	3073.0533
Hex4HexNAc4Neu5Ac2	**H4N4S2**	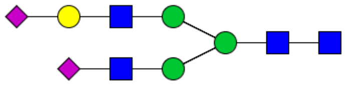	2060.7302
Hex5HexNAc4Neu5Ac2	**H5N4S2**	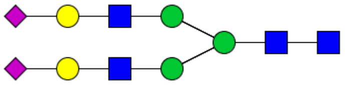	2222.7830
Hex6HexNAc4Neu5Ac2	**H6N4S2**	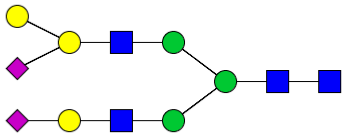	2384.8358
Hex5HexNAc5Neu5Ac2	**H5N5S2**	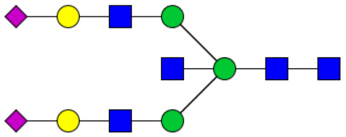	2425.8624
Hex7HexNAc4Neu5Ac2	**H7N4S2**	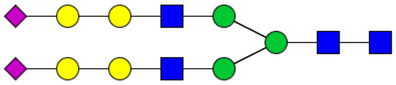	2546.8887
Hex6HexNAc5Neu5Ac2	**H6N5S2**	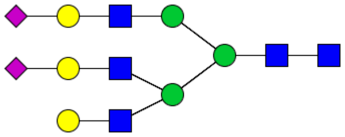	2587.9152
Hex6HexNAc5Neu5Ac2	**H6N5S2**	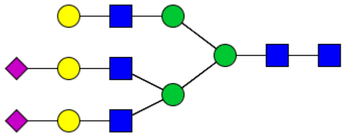	2587.9152
Hex6HexNAc5Neu5Ac2	**H6N5S2**	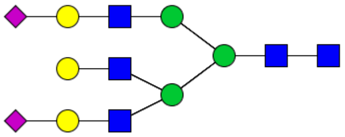	2587.9152

Hex6HexNAc6Neu5Ac2	**H6N6S2**	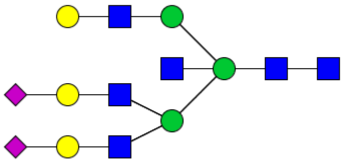	2790.9946
Hex7HexNAc6Neu5Ac2	**H7N6S2**	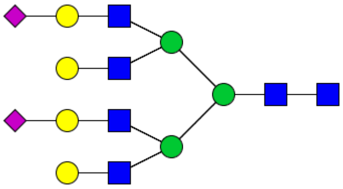	2953.0474
Fuc1Hex5HexNAc4Neu5Ac2	**F1H5N4S2**	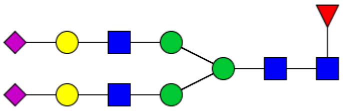	2368.8409
Fuc1Hex5HexNAc4Neu5Ac2	**F1H5N4S2**	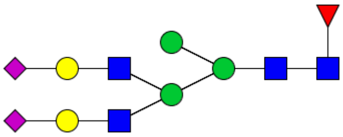	2368.8409
Fuc1Hex6HexNAc4Neu5Ac2	**F1H6N4S2**	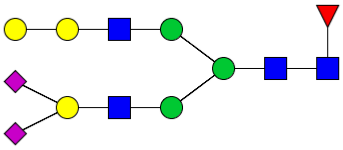	2530.8937
Fuc1Hex5HexNAc5Neu5Ac2	**F1H5N5S2**	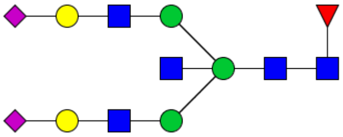	2571.9203
Fuc1Hex6HexNAc5Neu5Ac2	**F1H6N5S2**	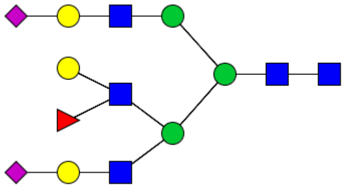	2733.9731
Fuc1Hex7HexNAc5Neu5Ac2	**F1H7N5S2**	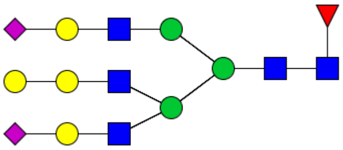	2896.0259
Fuc1Hex7HexNAc6Neu5Ac2	**F1H7N6S2**	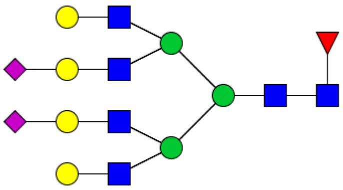	3099.1053

Fuc1Hex9HexNAc6Neu5Ac2	**F1H9N6S2**	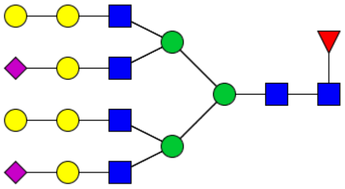	3423.2110
Fuc1Hex8HexNAc7Neu5Ac2	**F1H8N7S2**	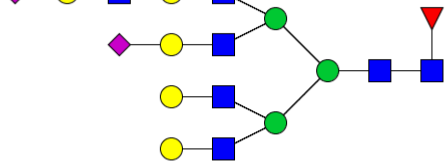	3464.2375
Fuc1Hex11HexNAc8Neu5Ac2	**F1H11N8S2**	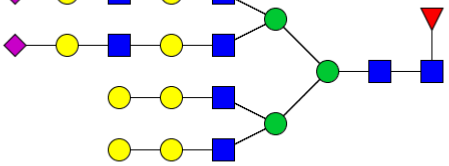	4153.4753
Fuc2Hex5HexNAc5Neu5Ac2	**F2H5N5S2**	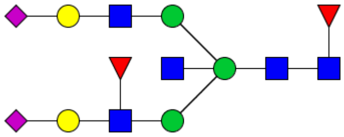	2717.9782
Hex5HexNAc4Neu5Ac1Neu5Gc1	**H5N4S2**	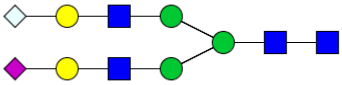	2238.7779
Hex5HexNAc4Neu5Gc2	**H5N4S2**	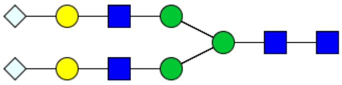	2254.7728
Fuc1Hex5HexNAc4Neu5Ac1Neu5Gc1	**F1H5N4S2**	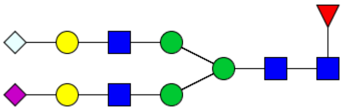	2384.8358
Fuc1Hex5HexNAc4Neu5Gc2	**F1H5N4S2**	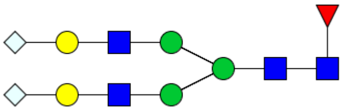	2400.8307
Hex4HexNAc3Neu5Ac1	**H4N3S1**	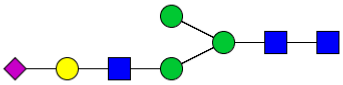	1566.5554
Hex5HexNAc4Neu5Ac1	**H5N4S1**	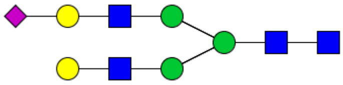	1931.6876

Hex6HexNAc4Neu5Ac1	**H6N4S1**	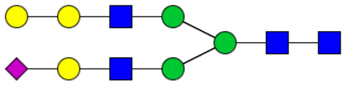	2093.7404
Hex6HexNAc5Neu5Ac1	**H6N5S1**	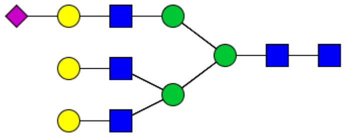	2296.8198
Hex6HexNAc5Neu5Ac1	**H6N5S1**	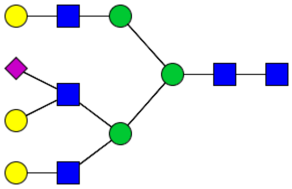	2296.8198
Fuc1Hex5HexNAc4Neu5Ac1	**F1H5N4S1**	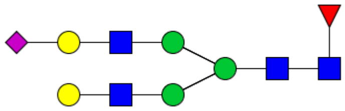	2077.7455
Fuc1Hex6HexNAc5Neu5Ac1	**F1H6N5S1**	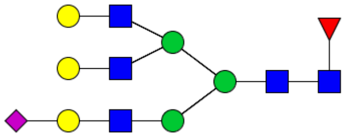	2442.8777
Fuc1Hex10HexNAc6Neu5Ac1	**F1H10N6S1**	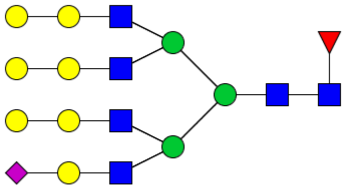	3294.1684
Fuc2Hex4HexNAc4Neu5Ac1	**F2H4N4S1**	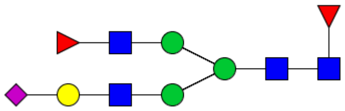	2061.7506
Fuc2Hex5HexNAc4Neu5Ac1	**F2H5N4S1**	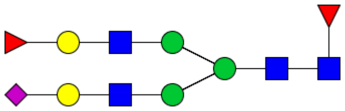	2223.8034
Fuc2Hex6HexNAc5Neu5Ac1	**F2H6N5S1**	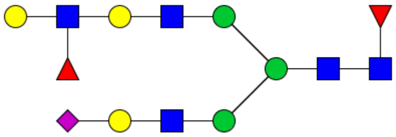	2588.9356

Fuc3Hex5HexNAc4Neu5Ac1	**F3H5N4S1**	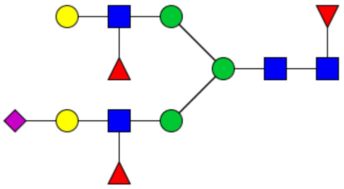	2369.8613
Fuc3Hex6HexNAc5Neu5Ac1	**F3H6N5S1**	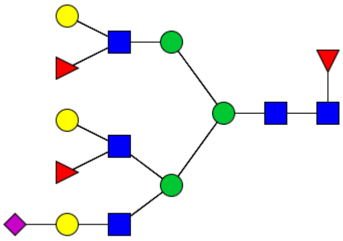	2734.9935
Fuc4Hex7HexNAc6Neu5Ac1	**F4H7N6S1**	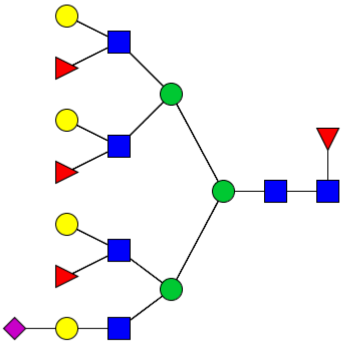	3246.1836
Hex5HexNAc4Neu5Gc1	**H5N4S1**	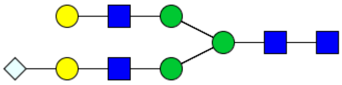	1947.6825
Fuc1Hex6HexNAc4Neu5Gc1	**F1H6N4S1**	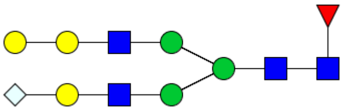	2255.7932
Hex4HexNAc3	**H4N3**	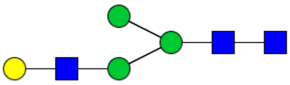	1275.4600
Hex5HexNAc4	**H5N4**	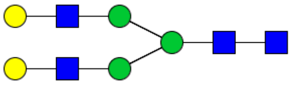	1640.5922
Hex4HexNAc5	**H4N5**	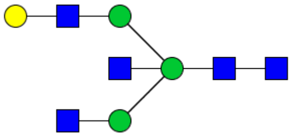	1681.6187
Hex7HexNAc4	**H7N4**	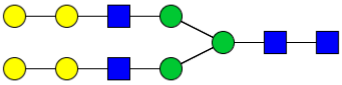	1964.6978

Hex6HexNAc5	**H6N5**	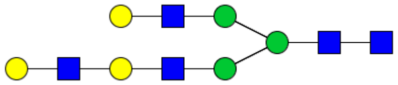	2005.7244
Hex7HexNAc5	**H7N5**	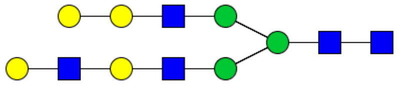	2167.7772
Hex8HexNAc5	**H8N5**	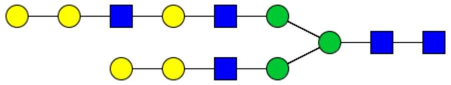	2329.8300
Hex7HexNAc6	**H7N6**	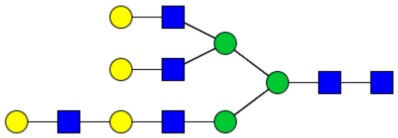	2370.8566
Hex7HexNAc7	**H7N7**	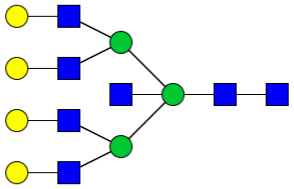	2573.9359
Hex6HexNAc9	**H6N9**	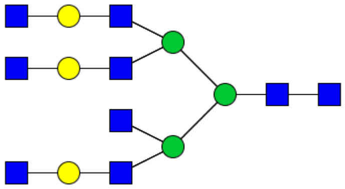	2818.0419
Fuc1Hex3HexNAc2	**F1H3N2**	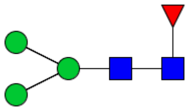	1056.3857
Fuc1Hex4HexNAc4	**F1H4N4**	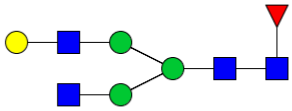	1624.5973
Fuc1Hex5HexNAc4	**F1H5N4**	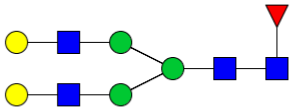	1786.6501
Fuc1Hex6HexNAc4	**F1H6N4**	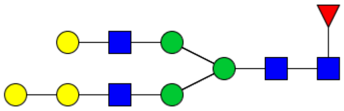	1948.7029

Fuc1Hex7HexNAc4	**F1H7N4**	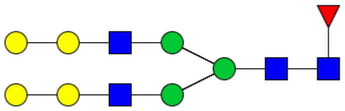	2110.7557
Fuc1Hex6HexNAc5	**F1H6N5**	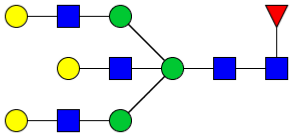	2151.7823
Fuc1Hex7HexNAc5	**F1H7N5**	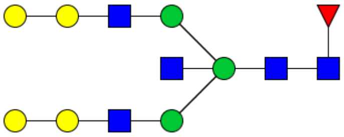	2313.8351
Fuc1Hex6HexNAc6	**F1H6N6**	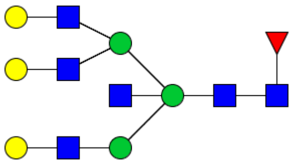	2354.8616
Fuc1Hex9HexNAc4	**F1H9N4**	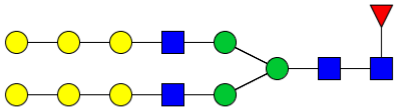	2434.8614
Fuc1Hex7HexNAc6	**F1H7N6**	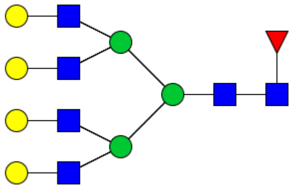	2516.9145
Fuc1Hex9HexNAc5	**F1H9N5**	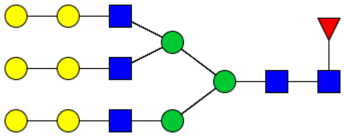	2637.9407
Fuc1Hex7HexNAc7	**F1H7N7**	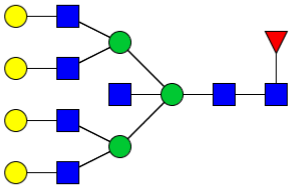	2719.9938

Fuc1Hex8HexNAc8	**F1H8N8**	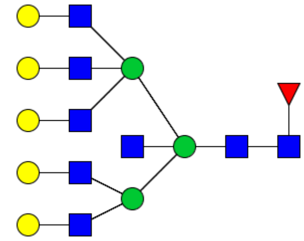	3085.1260
Fuc1Hex11HexNAc8	**F1H11N8**	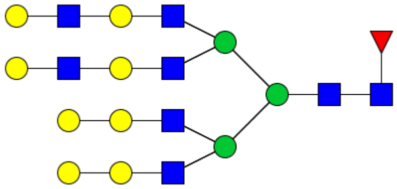	3571.2845
Fuc2Hex4HexNAc3	**F2H4N3**	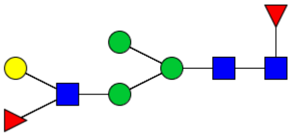	1567.5758
Fuc2Hex5HexNAc4	**F2H5N4**	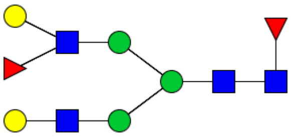	1932.7080
Fuc2Hex6HexNAc6	**F2H6N6**	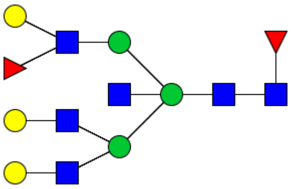	2500.9196
Fuc3Hex6HexNAc4	**F3H6N4**	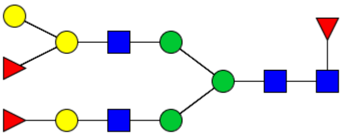	2240.8187
Fuc4Hex6HexNAc5	**F4H6N5**	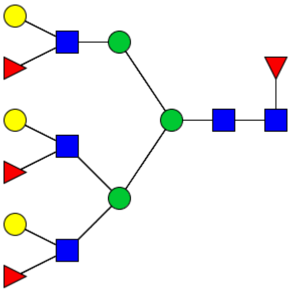	2589.9560

Fuc4Hex6HexNAc6	**F4H6N6**	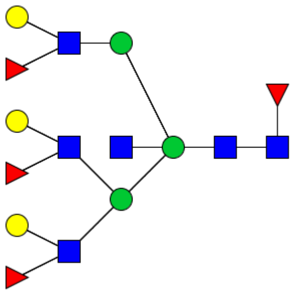	2793.0354
Fuc4Hex7HexNAc6	**F4H7N6**	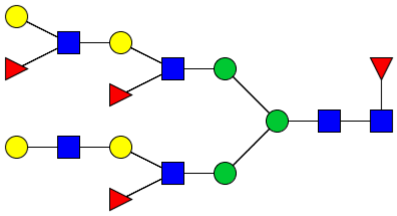	2955.0882
Hex4HexNAc2	**H4N2**	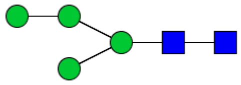	1072.3806
Hex5HexNAc2	**H5N2**	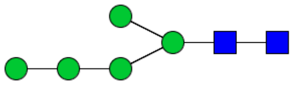	1234.4334
Hex6HexNAc2	**H6N2**	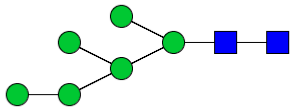	1396.4863
Hex6HexNAc2	**H6N2**	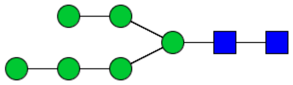	1396.4863
Hex11HexNAc2	**H11N2**	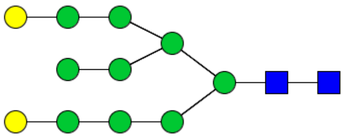	2206.7504
Fuc1Hex5HexNAc2	**F1H5N2**	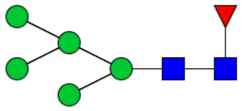	1380.4913
Hex5HexNAc3	**H5N3**	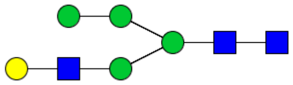	1437.5128

Hex6HexNAc3	**H6N3**	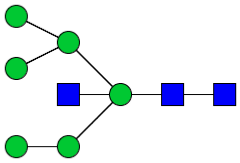	1599.5656
Hex9HexNAc3	**H9N3**	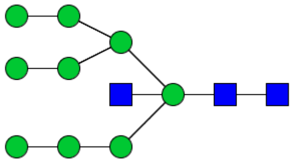	2085.7241
Fuc1Hex5HexNAc3	**F1H5N3**	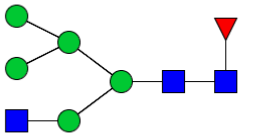	1583.5707
Fuc1Hex6HexNAc3	**F1H6N3**	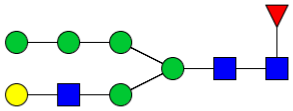	1745.6235
Hex5HexNAc3Neu5Ac1	**H5N3S1**	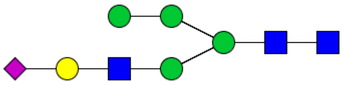	1728.6082
Hex6HexNAc3Neu5Ac1	**H6N3S1**	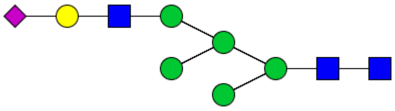	1890.6610
Fuc1Hex6HexNAc3Neu5Ac1	**F1H6N3S1**	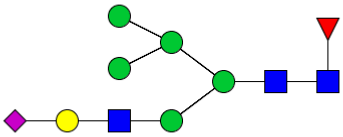	2036.7189
Hex9HexNAc5Neu5Ac2	**H9N5S2**	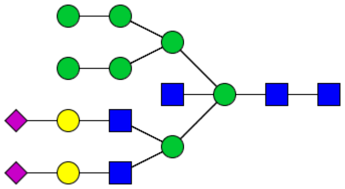	3074.0737
