# Supplementary material for: Highly-sensitive label-free deep profiling of N-glycans released from biomedically-relevant samples
Source: Nat Commun. 2023 Mar 23;14:1618. doi: 10.1038/s41467-023-37365-4 (PMC10036494; doi:10.1038/s41467-023-37365-4)
Supplement: Supplementary file 9 — Supplementary Data 7 [file 41467_2023_37365_MOESM9_ESM.pdf]

Bovine pancreas RNase B

Composition	Name	Structure	Mr_{th} (Da)
Hex6HexNAc5Neu5Ac3	**H6N5S3**	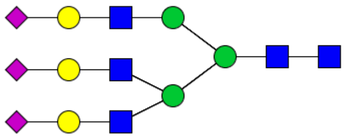	2879.0106
Hex6HexNAc5Neu5Ac2Neu5Gc1	**H6N5S3**	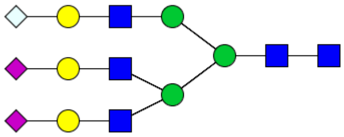	2895.0055
Fuc1Hex6HexNAc5Neu5Ac3	**F1H6N5S3**	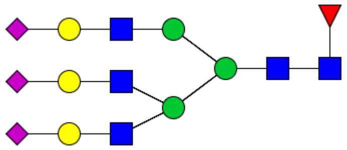	3025.0685
Fuc1Hex6HexNAc5Neu5Ac1Neu5Gc2	**F1H6N5S3**	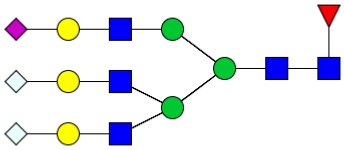	3057.0584
Hex6HexNAc6Neu5Ac3	**H6N6S3**	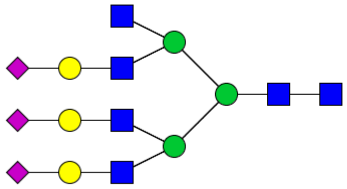	3082.0900
Fuc2Hex6HexNAc5Neu5Ac3	**F2H6N5S3**	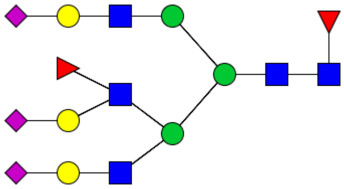	3171.1264
Hex5HexNAc4Neu5Ac2	**H5N4S2**	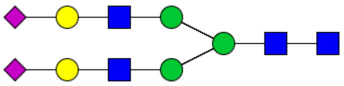	2222.7830
Hex5HexNAc4Neu5Ac1Neu5Gc1	**H5N4S2**	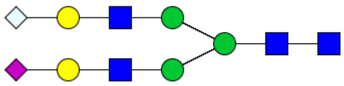	2238.7779
Hex5HexNAc4Neu5Gc2	**H5N4S2**	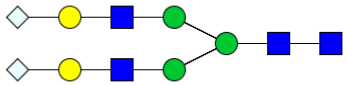	2254.7728

Fuc1Hex5HexNAc4Neu5Ac1Neu5Gc1	**F1H5N4S2**	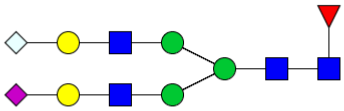	2384.8358
Fuc1Hex5HexNAc4Neu5Gc2	**F1H5N4S2**	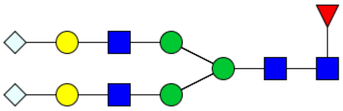	2400.8307
Hex6HexNAc4Neu5Ac2	**H6N4S2**	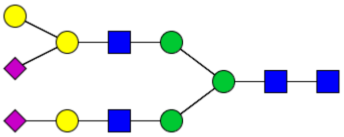	2384.8358
Hex5HexNAc4Neu5Ac1	**H5N4S1**	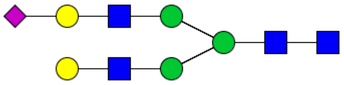	1931.6876
Hex5HexNAc4Neu5Gc1	**H5N4S1**	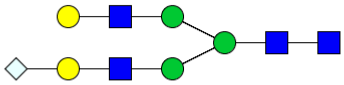	1947.6825
Fuc1Hex5HexNAc4Neu5Ac1	**F1H5N4S1**	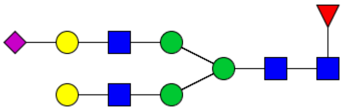	2077.7455
Fuc1Hex5HexNAc4Neu5Gc1	**F1H5N4S1**	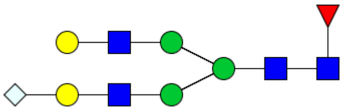	2093.7404
Fuc1Hex6HexNAc4Neu5Gc1	**F1H6N4S1**	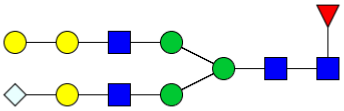	2255.7932
Hex5HexNAc4	**H5N4**	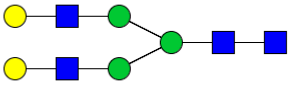	1640.5922
Fuc1Hex5HexNAc4	**F1H5N4**	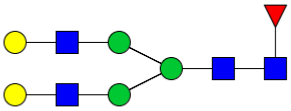	1786.6501
Fuc1Hex5HexNAc4	**F1H5N4**	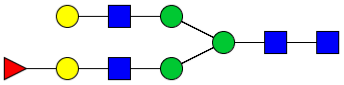	1786.6501

Hex3HexNAc2	**H3N2**	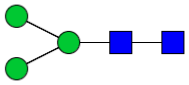	910.3278
Hex4HexNAc2	**H4N2**	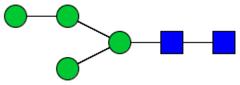	1072.3806
Hex5HexNAc2	**H5N2**	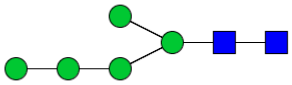	1234.4334
Hex6HexNAc2	**H6N2**	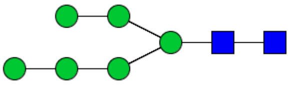	1396.4863
Hex7HexNAc2	**H7N2**	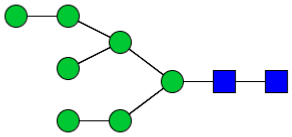	1558.5391
Hex8HexNAc2	**H8N2**	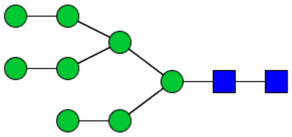	1720.5919
Hex9HexNAc2	**H9N2**	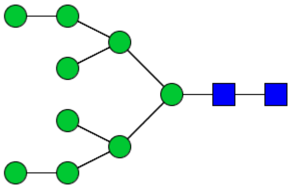	1882.6447
Hex10HexNAc2	**H10N2**	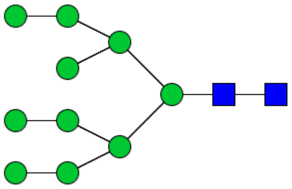	2044.6975
Hex11HexNAc2	**H11N2**	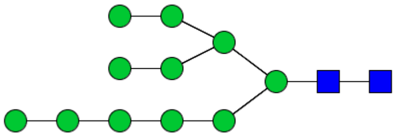	2206.7504
Hex12HexNAc2	**H12N2**	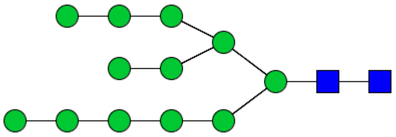	2368.8032

Hex13HexNAc2	**H13N2**	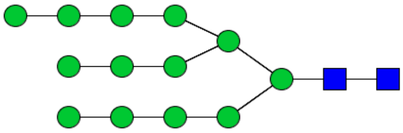	2530.8560
Fuc2Hex3HexNAc2	**F2H3N2**	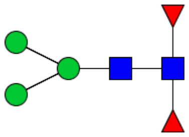	1202.4436
Fuc1Hex6HexNAc3	**F1H6N3**	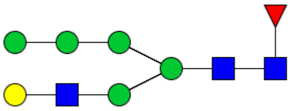	1745.6235
Fuc1Hex5HexNAc4	**F1H5N4**	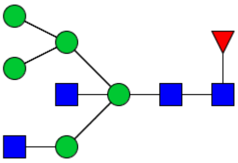	1786.6501
Fuc1Hex6HexNAc3	**F1H6N3**	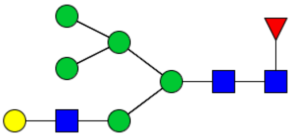	1745.6235
Hex6HexNAc3Neu5Ac1	**H6N3S1**	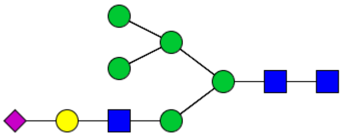	1890.6610
