# Supplementary material for: Highly-sensitive label-free deep profiling of N-glycans released from biomedically-relevant samples
Source: Nat Commun. 2023 Mar 23;14:1618. doi: 10.1038/s41467-023-37365-4 (PMC10036494; doi:10.1038/s41467-023-37365-4)
Supplement: Supplementary file 10 — Supplementary Data 8 [file 41467_2023_37365_MOESM10_ESM.pdf]

Human plasma EVs

Composition	Name	Structure	Mr_{th} (Da)
Fuc1Hex6HexNAc5	**F1H6N5**	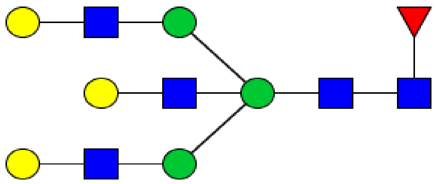	2151.7823
Hex5HexNAc4Neu5Ac1	**H5N4S1**	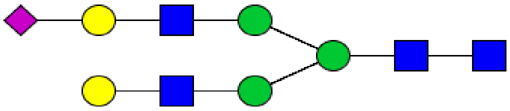	1931.6876
Hex6HexNAc5Neu5Ac1	**H6N5S1**	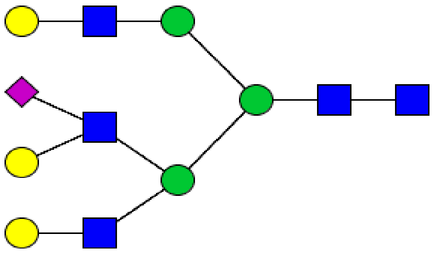	2296.8198
Hex6HexNAc5Neu5Ac1	**H6N5S1**	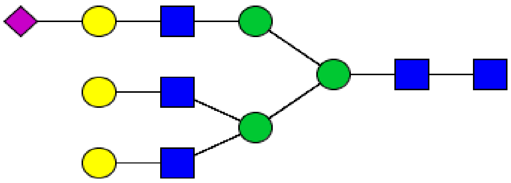	2296.8198
Fuc1Hex5HexNAc4Neu5Ac1	**F1H5N4S1**	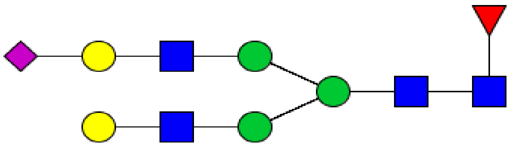	2077.7455
Fuc1Hex5HexNAc5Neu5Ac1	**F1H5N5S1**	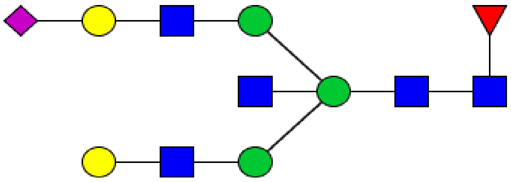	2280.8249

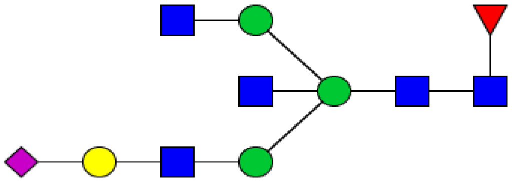

Fuc1Hex4HexNAc5Neu5Ac1 **F1H4N5S1** 2118.7720

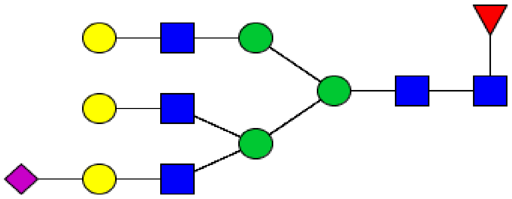

Fuc1Hex6HexNAc5Neu5Ac1 **F1H6N5S1** 2442.8777

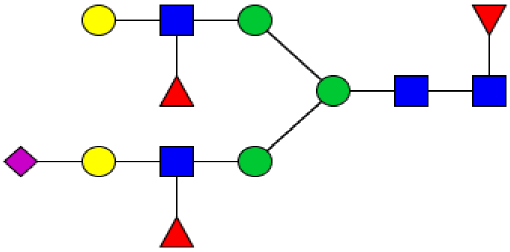

Fuc3Hex5HexNAc4Neu5Ac1 **F3H5N4S1** 2369.8613

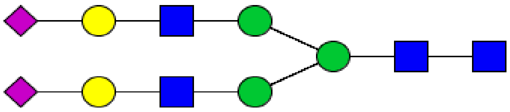

Hex5HexNAc4Neu5Ac2 **H5N4S2** 2222.7830

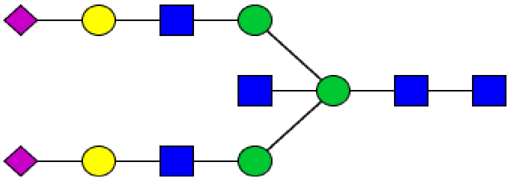

Hex5HexNAc5Neu5Ac2 **H5N5S2** 2425.8624

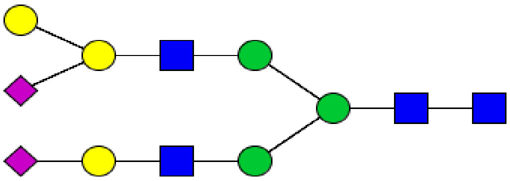

Hex6HexNAc4Neu5Ac2 **H6N4S2** 2384.8358

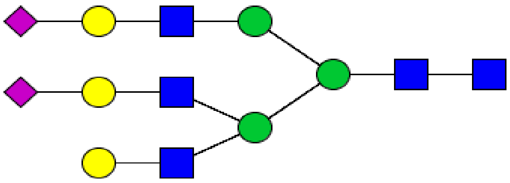

Hex6HexNAc5Neu5Ac2 **H6N5S2** 2587.9152

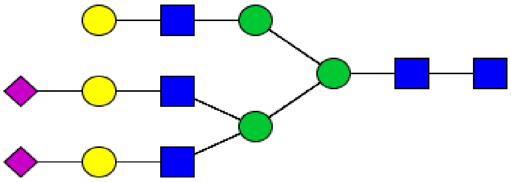

Hex6HexNAc5Neu5Ac2 **H6N5S2** 2587.9152

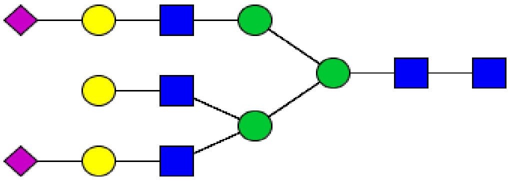

Hex6HexNAc5Neu5Ac2 **H6N5S2** 2587.9152

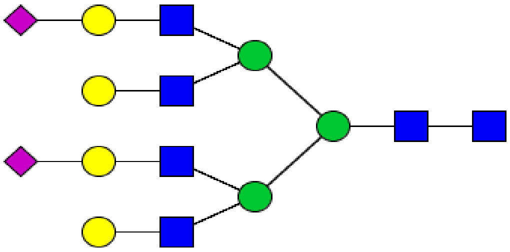

Hex7HexNAc6Neu5Ac2 **H7N6S2** 2953.0474

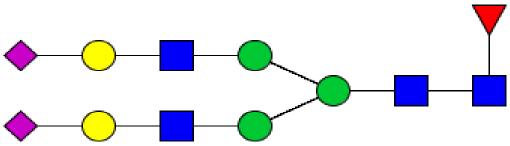

Fuc1Hex5HexNAc4Neu5Ac2 **F1H5N4S2** 2368.8409

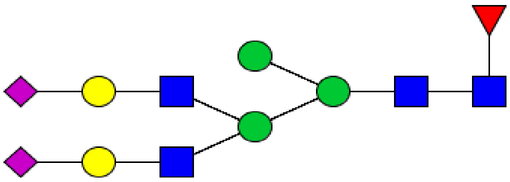

Fuc1Hex5HexNAc4Neu5Ac2 **F1H5N4S2** 2368.8409

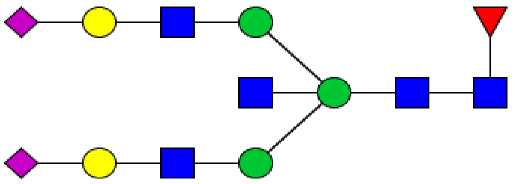

Fuc1Hex5HexNAc5Neu5Ac2 **F1H5N5S2** 2571.9203

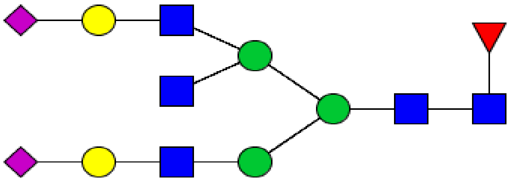

Fuc1Hex5HexNAc5Neu5Ac2 **F1H5N5S2** 2571.9203

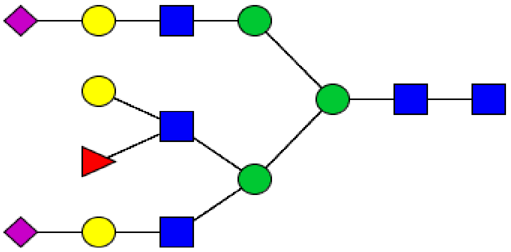

Fuc1Hex6HexNAc5Neu5Ac2 **F1H6N5S2** 2733.9731

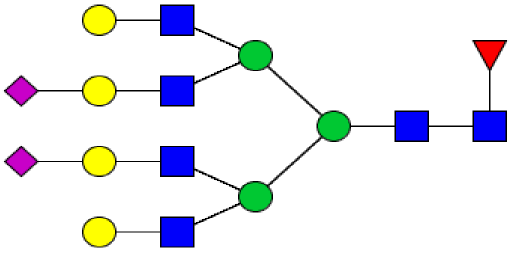

Fuc1Hex7HexNAc6Neu5Ac2 **F1H7N6S2** 3099.1053

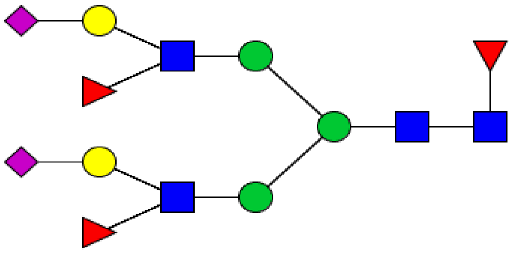

Fuc3Hex5HexNAc4Neu5Ac2 **F3H5N4S2** 2660.9567

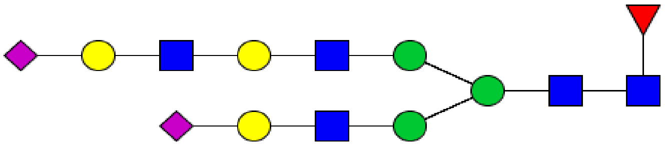

Fuc1Hex6HexNAc5Neu5Ac2 **F1H6N5S2** 2733.9731

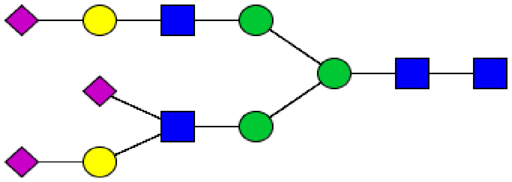

Hex5HexNAc4Neu5Ac3 **H5N4S3** 2513.8784

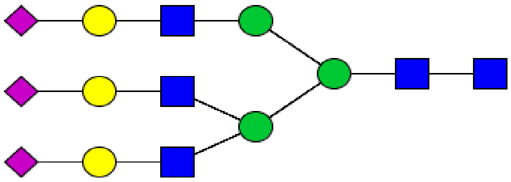

Hex6HexNAc5Neu5Ac3 **H6N5S3** 2879.0106

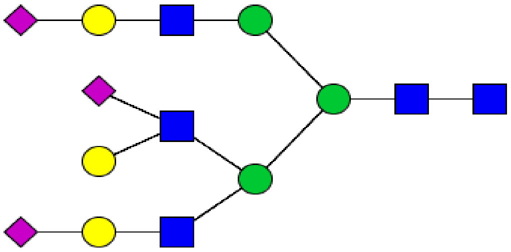

Hex6HexNAc5Neu5Ac3 **H6N5S3** 2879.0106

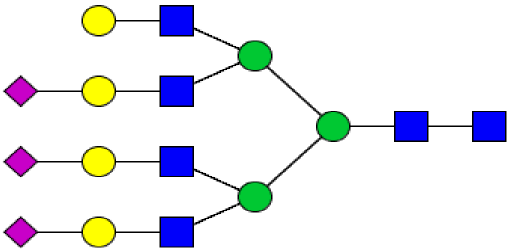

Hex7HexNAc6Neu5Ac3 **H7N6S3** 3244.1428

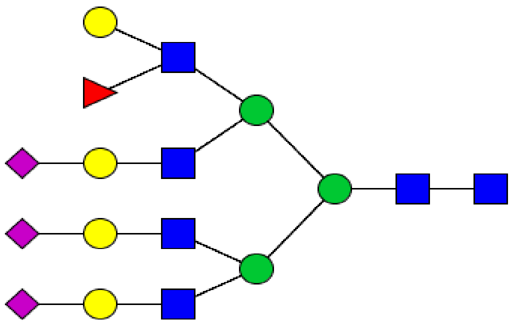

Fuc1Hex7HexNAc6Neu5Ac3 **F1H7N6S3** 3390.2007

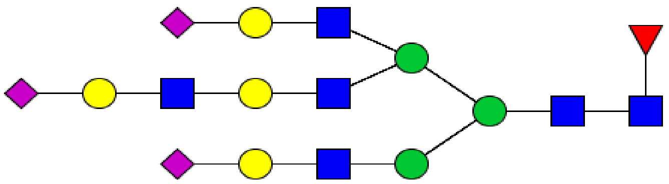

Fuc1Hex7HexNAc6Neu5Ac3 **F1H7N6S3** 3390.2007

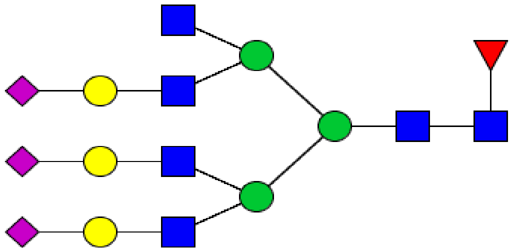

Fuc1Hex6HexNAc6Neu5Ac3 **F1H6N6S3** 3228.1479

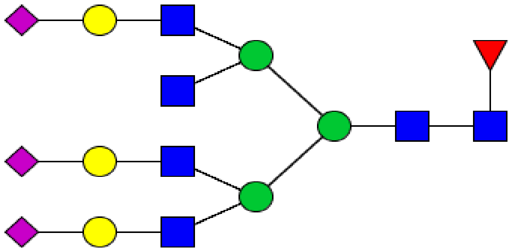

Fuc1Hex6HexNAc6Neu5Ac3 **F1H6N6S3** 3228.1479

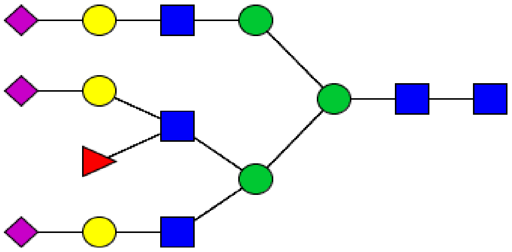

Fuc1Hex6HexNAc5Neu5Ac3 **F1H6N5S3** 3025.0685

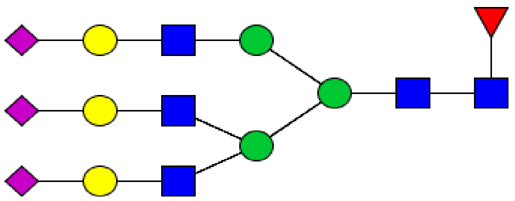

Fuc1Hex6HexNAc5Neu5Ac3 **F1H6N5S3** 3025.0685

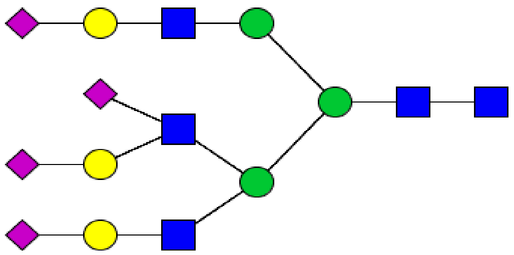

Hex6HexNAc5Neu5Ac4 **H6N5S4** 3170.1060

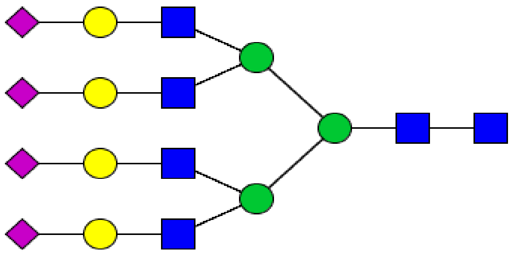

Hex7HexNAc6Neu5Ac4 **H7N6S4** 3535.2382

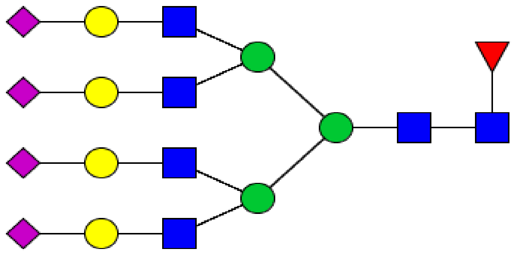

Fuc1Hex7HexNAc6Neu5Ac4 **F1H7N6S4** 3681.2961

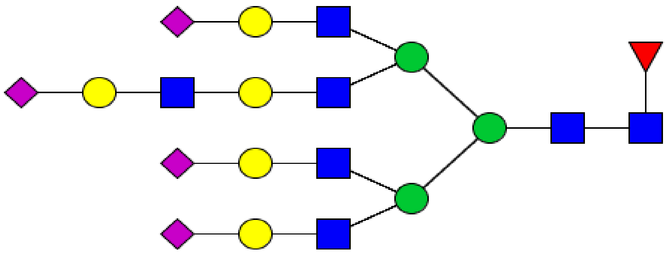

Fuc1Hex8HexNAc7Neu5Ac4 **F1H8N7S4** 4046.4283

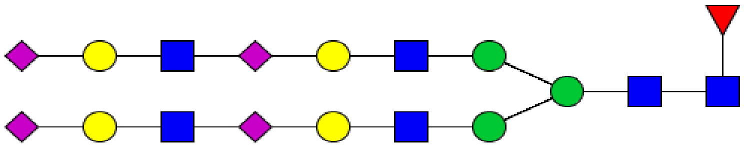

Fuc1Hex7HexNAc6Neu5Ac4 **F1H7N6S4** 3681.2961

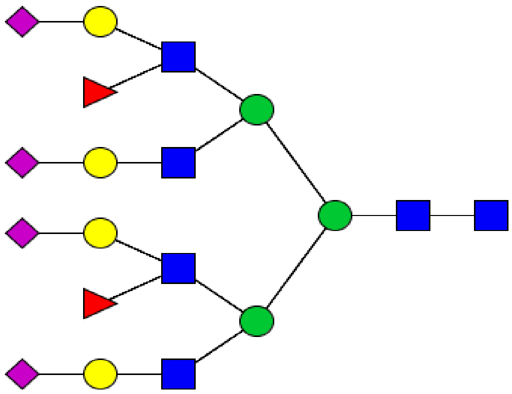

Fuc2Hex7HexNAc6Neu5Ac4 **F2H7N6S4** 3827.3540

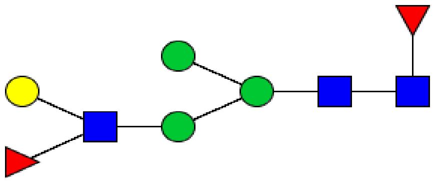

Fuc2Hex4HexNAc3 **F2H4N3** 1567.5758

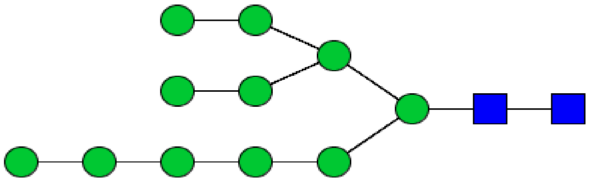

Hex11HexNAc2 **H11N2** 2206.7504

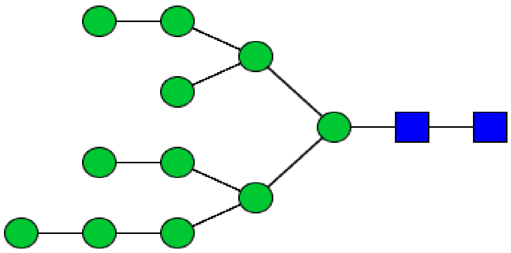

Hex11HexNAc2 **H11N2** 2206.7504

Fuc1Hex9HexNAc2	**F1H9N2**	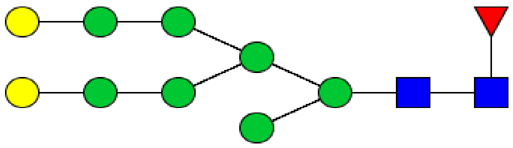	2028.7026
Hex10HexNAc2	**H10N2**	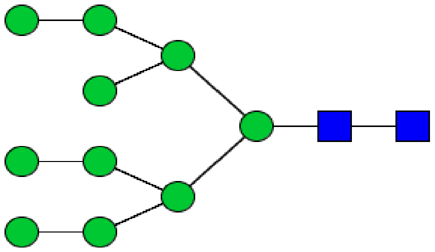	2044.6975
Hex5HexNAc3Neu5Ac1	**H5N3S1**	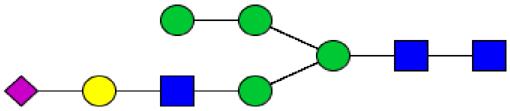	1728.6082
Fuc1Hex6HexNAc3Neu5Ac1	**F1H6N3S1**	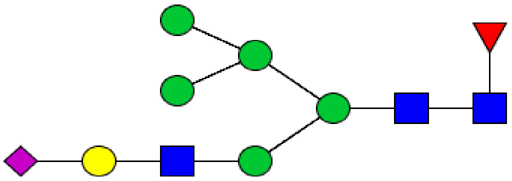	2036.7189
Hex5HexNAc4Neu5Ac1Neu5Gc1	**H5N4S2**	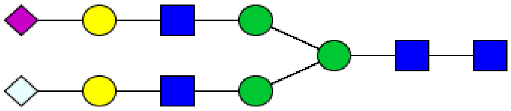	2238.7779
Hex5HexNAc4Neu5Gc2	**H5N4S2**	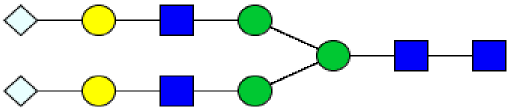	2254.7728
Fuc1Hex5HexNAc4Neu5Gc2	**F1H5N4S2**	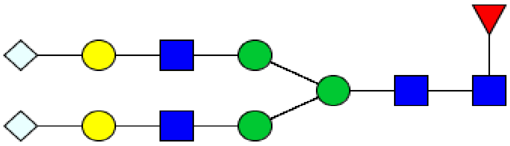	2400.8307
