# Supplementary material for: Highly-sensitive label-free deep profiling of N-glycans released from biomedically-relevant samples
Source: Nat Commun. 2023 Mar 23;14:1618. doi: 10.1038/s41467-023-37365-4 (PMC10036494; doi:10.1038/s41467-023-37365-4)
Supplement: Supplementary file 13 — Reporting Summary [file 41467_2023_37365_MOESM13_ESM.pdf]

nature portfolio

Corresponding author(s): Alexander R. Ivanov

Last updated by author(s): 02/19/2023

Reporting Summary

Nature Portfolio wishes to improve the reproducibility of the work that we publish. This form provides structure for consistency and transparency in reporting. For further information on Nature Portfolio policies, see our Editorial Policies and the Editorial Policy Checklist.

Statistics

For all statistical analyses, confirm that the following items are present in the figure legend, table legend, main text, or Methods section.

n/a	Confirmed	
[x]	[x]	The exact sample size (*n*) for each experimental group/condition, given as a discrete number and unit of measurement
[x]	[ ]	A statement on whether measurements were taken from distinct samples or whether the same sample was measured repeatedly
[x]	[ ]	The statistical test(s) used AND whether they are one- or two-sided<br>*Only common tests should be described solely by name; describe more complex techniques in the Methods section.*
[x]	[ ]	A description of all covariates tested
[x]	[ ]	A description of any assumptions or corrections, such as tests of normality and adjustment for multiple comparisons
[ ]	[x]	A full description of the statistical parameters including central tendency (e.g. means) or other basic estimates (e.g. regression coefficient) AND variation (e.g. standard deviation) or associated estimates of uncertainty (e.g. confidence intervals)
[x]	[ ]	For null hypothesis testing, the test statistic (e.g. *F*, *t*, *r*) with confidence intervals, effect sizes, degrees of freedom and *P* value noted<br>*Give P values as exact values whenever suitable.*
[x]	[ ]	For Bayesian analysis, information on the choice of priors and Markov chain Monte Carlo settings
[x]	[ ]	For hierarchical and complex designs, identification of the appropriate level for tests and full reporting of outcomes
[x]	[ ]	Estimates of effect sizes (e.g. Cohen's *d*, Pearson's *r*), indicating how they were calculated

Our web collection on statistics for biologists contains articles on many of the points above.

Software and code

Policy information about availability of computer code

Data collection	For data acquisition, Xcalibur (v. 2.8) software (Thermo Fisher Scientific, Bremen, Germany) was used.
Data analysis	For data processing, Xcalibur (v. 2.8) software (Thermo Fisher Scientific, Bremen, Germany) was used. CZE-MS data were processed with GlycReSoft (v. 3.10) software (Boston University, Boston, MA, USA). Analyses of CZE-MS2 data were performed with SimGlycan (v. 5.91) software (Premier Biosoft, Palo Alto, CA, USA). The R (version 4.05) packages “pheatmap” (version 1.0.12) and “circlize” (version 0.4.14) were used respectively to generate linear and circular clustered heatmaps. The bar charts with individual data points, mean values and error bars were plotted with R statistical analysis software.

For manuscripts utilizing custom algorithms or software that are central to the research but not yet described in published literature, software must be made available to editors and reviewers. We strongly encourage code deposition in a community repository (e.g. GitHub). See the Nature Portfolio guidelines for submitting code & software for further information.

Data

Policy information about availability of data

All manuscripts must include a data availability statement. This statement should provide the following information, where applicable:

- Accession codes, unique identifiers, or web links for publicly available datasets
- A description of any restrictions on data availability
- For clinical datasets or third party data, please ensure that the statement adheres to our policy

All data required to evaluate the results and conclusion discussed in the manuscript are present in the main manuscript and in the Supplementary Information and Source Data file provided with this paper. The raw data generated in this study have been deposited in GlycoPOST (GPST000122 and GPST000281) (https://glycopost.glycosmos.org).

Human research participants

Policy information about studies involving human research participants and Sex and Gender in Research.

Reporting on sex and gender	Male donors
Population characteristics	Self-declared healthy male donors of the age 23 - 67 years old.
Recruitment	Twelve self-declared healthy male donors were selected and informed consent was obtained for each participant.
Ethics oversight	The research study involving human subjects was reviewed by the respective authorized Institutional Review Boards (IRB) with approvals IRB#2001P000S91 (BIDMC) and IRB#17-12-14 (NU)

Note that full information on the approval of the study protocol must also be provided in the manuscript.

Field-specific reporting

Please select the one below that is the best fit for your research. If you are not sure, read the appropriate sections before making your selection.

[x] Life sciences [ ] Behavioural & social sciences [ ] Ecological, evolutionary & environmental sciences

For a reference copy of the document with all sections, see nature.com/documents/nr-reporting-summary-flat.pdf

Life sciences study design

All studies must disclose on these points even when the disclosure is negative.

Sample size	N/A No sample size calculations were performed since the selected number of replicate experiments was chosen to reliably determine the reproducibility and standard deviation.
Data exclusions	Infrequent failures in experiments were caused by hardware malfunction (mechanical capillary damage). Such failures led to unsuccessful measurements that were excluded from this study.
Replication	The run-to-run and day-to-day reproducibility was assessed using at least three replicates. Infrequent failures in experiments were caused by hardware malfunction (mechanical capillary damage). Such failures led to unsuccessful measurements that were excluded from this study.
Randomization	N/A Different types of model samples were selected for evaluation of the developed approach. Since the comparative analysis of the selected samples was not the goal of this study, randomization was not appropriate in this study.
Blinding	N/A Different types of model samples were selected for evaluation of the developed approach. Since the comparative analysis of the selected samples was not the goal of this study, blinding was not appropriate in this study.

Reporting for specific materials, systems and methods

We require information from authors about some types of materials, experimental systems and methods used in many studies. Here, indicate whether each material, system or method listed is relevant to your study. If you are not sure if a list item applies to your research, read the appropriate section before selecting a response.

Materials & experimental systems

n/a	Involved in the study	
[x]	[ ]	Antibodies
[x]	[ ]	Eukaryotic cell lines
[x]	[ ]	Palaeontology and archaeology
[x]	[ ]	Animals and other organisms
[x]	[ ]	Clinical data
[x]	[ ]	Dual use research of concern

Methods

n/a	Involved in the study	
[x]	[ ]	ChIP-seq
[x]	[ ]	Flow cytometry
[x]	[ ]	MRI-based neuroimaging
